# Imidazole-Based AT_1_ Receptor Ligands: Design, Synthesis and Pharmacological Evaluation

**DOI:** 10.3390/molecules31111971

**Published:** 2026-06-05

**Authors:** Florian Descamps, Marouane Rami, Jean-François Goossens, Patricia Melnyk, Maxime Liberelle, Saïd Yous

**Affiliations:** 1 Univ. Lille, Inserm, CHU Lille, U1172—LilNCog—Lille Neuroscience & Cognition, 59000 Lille, France; florian.descamps@univ-lille.fr (F.D.); patricia.melnyk@univ-lille.fr (P.M.); 2 University Lille, CHU Lille, ULR 7365 GRITA—Groupe de Recherche sur les Formes Injectables et Technologies Associées, 59000 Lille, France

**Keywords:** angiotensin II type 1 receptor, imidazole-based antagonists, structure–activity relationships, GPCR drug design

## Abstract

The angiotensin II type 1 (AT_1_) receptor is a key component of the renin–angiotensin system (RAS) and a validated target for cardiovascular and renal disorders. Developing small molecules with defined AT_1_ versus AT_2_ binding profiles remains important for both therapeutic and mechanistic studies. Here, a series of novel imidazole-based compounds was synthesized and evaluated for their binding affinities toward angiotensin II type 1 (AT_1_) and type 2 (AT_2_) receptors. Binding studies were conducted by measuring the displacement of radiolabeled [^3^H]-angiotensin II ([^3^H]-AII) in PLC-PRF-5 human hepatoma cells for AT_1_ receptors and calf cerebellum membranes for AT_2_ receptors. Structure–activity relationship (SAR) analysis revealed that sulfonamide substitution significantly enhanced AT_1_ receptor affinity, whereas sterically hindered derivatives and ester-containing compounds were less active. Molecular docking studies using the AT_1_ receptor crystal structure (PDB: 8TH4) rationalized the observed activity trends. The most active compound showed high AT_1_ affinity (Ki = 5 nM), comparable to losartan, and all compounds displayed preferential binding for AT_1_ over AT_2_ receptors.

## 1. Introduction

The renin–angiotensin system (RAS) plays a central role in the regulation of cardiovascular homeostasis and blood pressure. Angiotensin II, the main effector peptide of the RAS, exerts its physiological and pathological effects primarily through activation of the angiotensin II type 1 (AT_1_) receptor, which mediates vasoconstriction, aldosterone secretion, sympathetic activation, and vascular remodeling [1,2]. In contrast, the AT_2_ receptor is generally associated with counter-regulatory and protective effects [3]. Consequently, selective AT_1_ receptor antagonists (angiotensin receptor blockers, ARBs) have become major therapeutic agents in the management of hypertension and related cardiovascular disorders [4].

**Figure 1:**
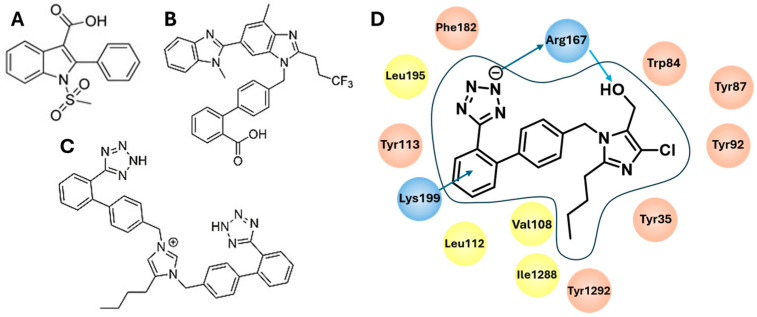
Recently published nonpeptide AT1 antagonists with examples of non-sartan (**A**), sartan (**B**) and bisartan (**C**) derivatives [5]. (**D**) Binding map for sartan derivatives, showing losartan and the main amino acids from AT_1_ from PDB 8TH4.

After nearly a decade with very few newly described inhibitors, novel nonpeptide AT1 antagonists have recently begun to emerge and can be broadly classified into three main categories: sartan derivatives, non-sartan derivatives, and bisartan derivatives [5] (Figure 1). Sartan derivatives, mainly inspired by losartan, are generally built around a biphenyl scaffold bearing an acidic or acidic-mimetic moiety that anchors the ligand within the orthosteric pocket; small changes around this pharmacophore strongly modulate potency and selectivity [6,7]. Medicinal chemistry and review articles summarize how substitutions at the biphenyl 2′-position and adjacent R_1_/R_2_ sites, as well as bioisosteric replacement of the acidic function, influence receptor recognition and pharmacokinetic properties [8,9,10].

The selection of a losartan-inspired scaffold was based on the well-established structure–activity relationships of nonpeptide AT_1_ receptor antagonists. Previous studies demonstrated that the biphenyl-tetrazole moiety plays a crucial role in anchoring the ligand within the receptor binding pocket, while the imidazole nucleus tolerates structural modifications at the C4 and C5 positions that can significantly influence receptor affinity and pharmacokinetic properties. In particular, substitutions at the biphenyl acidic function and at the imidazole ring were shown to modulate AT_1_/AT_2_ selectivity, duration of action and oral bioavailability. Furthermore, hydrophobic interactions involving the biphenyl system together with ionic interactions of the acidic group are considered essential for effective AT1 receptor recognition (Figure 1). These observations provided the rationale for the design of the present losartan-derived imidazole analogs. Building on this established pharmacophore, we designed a series of imidazole-based biphenyl derivatives to systematically explore key structural determinants of AT_1_ receptor binding. The study was organized around three main parameters, examined sequentially: (i) the nature of the ionizable acidic or acidic-mimetic group at the biphenyl 2′-position from tetrazole to ester, carboxylic acid, sulfonamide and N-acyl sulfonamide; (ii) the oxidation state of the benzylic linker connecting the imidazole and biphenyl units (secondary alcohol, methylene, ketone); and (iii) substitution patterns at aromatic R_2_ position to probe steric and electronic effects within the binding pocket. These variations were designed to evaluate how ionic anchoring interactions, particularly with the conserved Lys199 residue together with hydrophobic and aromatic contacts in the orthosteric pocket, govern receptor affinity (Figure 1). In parallel, the series enabled assessment of how polarity, conformational preference, and steric constraints modulate the ability of non-classical acidic motifs, such as N-acyl sulfonamides, to mimic tetrazole or carboxylate anchors [6,8,11].

In this work, we report the synthesis and biological evaluation of this series. Binding affinities were determined by displacement of radiolabeled [^3^H]-angiotensin II at human AT_1_ (PLC-PRF-5 cells) and at AT_2_ (calf cerebellum membranes). To rationalize experimental SAR trends, retrospective molecular docking was performed using the antagonist-bound AT_1_ crystal structure (PDB: 8TH4). The combined experimental and computational analyses identify key structural determinants: linker oxidation state, sulfonamide substitution, and presence of an ionizable anchor that govern AT_1_ affinity and selectivity in these new losartan-inspired imidazole derivatives.

## 2. Results and Discussion

### 2.1. Chemical Design

A novel series of imidazole-based derivatives was designed by incorporating either a biphenylmethylene or a benzophenone linker, two structural motifs commonly encountered in clinically validated angiotensin II type 1 (AT_1_) receptor antagonists. Structural diversification focused on substituents at the biphenyl 2′-position (R_1_) and at the 2-alkoxyphenyl position (R_2_) (Table 1).

A butyl chain was deliberately conserved at the imidazole 2-position, consistent with pharmacophoric features identified in losartan-like AT_1_ antagonists. This substitution was maintained to preserve key hydrophobic interactions and ensure comparability within the series.

The present work builds on earlier key contributions from our laboratory, which reported pyrazolidine-3,5-dione derivatives as potent and selective AT_1_ receptor antagonists capable of replacing classical imidazole cores. Those investigations demonstrated that scaffold rigidity and the precise spatial orientation of acidic moieties are critical determinants for receptor recognition and antagonist potency [6,7].

Expanding on this framework, we explored systematic variations at R_1_ and R_2_. Sulfonamide substitution patterns were modulated to evaluate steric effects, lipophilic contributions, and the capacity of N-acyl sulfonamides to act as bioisosteres of acidic functions. In parallel, linker oxidation states (secondary alcohol, methylene, ketone) were varied to probe the influence of flexibility, polarity, and conformational constraints on receptor affinity.

Finally, the requirement for an ionizable acidic group was investigated by comparing ester and carboxylic acid analogs. In addition, a tetrazole derivative was synthesized as an alternative polar bioisostere, enabling evaluation of non-classical acidic replacements within the series.

### 2.2. Rational Design

**Scheme 1:**
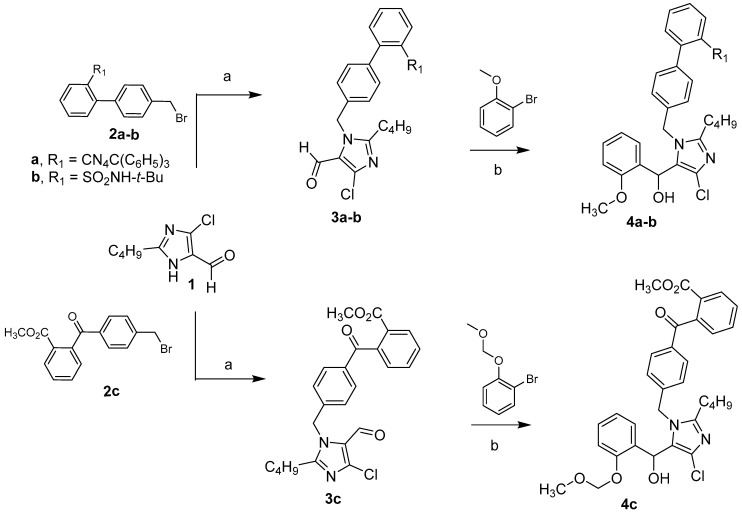
Synthesis of **4a**–**c**. Reagents and conditions: (a) K_2_CO_3_, DMF, 70 °C, 77% for **3a**, 78% for **3b**, 77% for **3c**; (b) Mg, anhydrous THF, 50 °C, 72% for **4a**, 75% for **4b**,73% for **4c**.

The synthesis of secondary alcohols **4a**–**c** was accomplished through a two-step reaction sequence (Scheme 1). In the first step (a), N-alkylation of imidazole **1** [10] with bromomethyl derivatives **2a**–**c** [12,13,14] was carried out in DMF at 70 °C using K_2_CO_3_ as the base [6,8]. This reaction afforded the N-alkylated intermediates **3a**–**c** in good yields (77%, 78%, and 77%, respectively). In the second step (b), secondary alcohols **4a**–**c** were prepared via a Grignard-type reaction. Magnesium metal was reacted with the corresponding bromobenzene derivatives [6] to form the organomagnesium reagents, which subsequently reacted with the aldehyde function of **3a**–**c**, yielding secondary alcohols **4a**–**c** in 72%, 75%, and 73% yields, respectively.

**Scheme 2:**
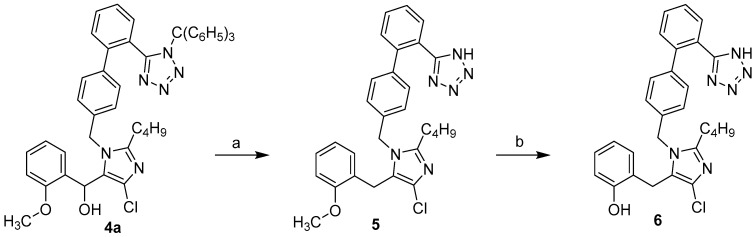
Synthesis of **5**–**6**. Reagents and conditions: (a) (Et)_3_SiH, TFA, rt, 85%; (b) BBr_3_, DCM, rt, 65%.

Scheme 2 outlines the two-step synthesis of compound **6** starting from alcohol **4a**. In step (a), the secondary alcohol of **4a** was selectively reduced to a methylene group using triethylsilane and trifluoroacetic acid at room temperature. Simultaneously, the tetrazole protecting group was removed, affording compound **5** in 85% yield. In step (b), compound **5** underwent selective demethylation of the methoxy group upon treatment with BBr_3_ in DCM, producing compound **6** in 65% yield. This sequence illustrates an efficient strategy combining alcohol reduction and protecting group removal.

**Scheme 3:**
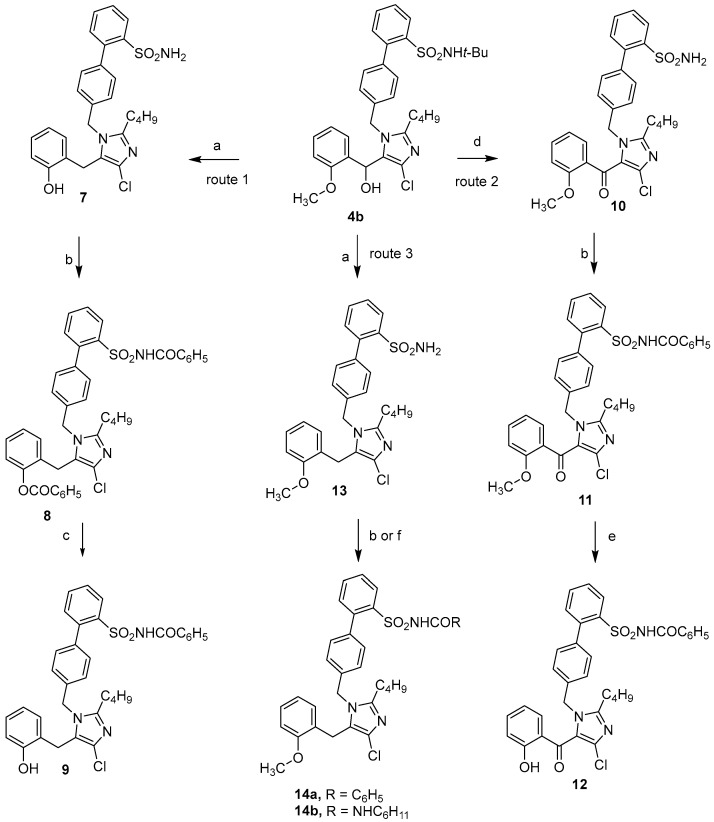
Synthesis of **9**–**14**. Reagents and conditions: (a) (i) (Et)_3_SiH, CF_3_CO_2_H, rt, (ii) BBr_3_, DCM, 72% for 7, 52% for **13**; (b) (C_6_H_5_CO)_2_O, pyridine, 80 °C, 85% for **8**, 82% for **11**, 76% for **14a**; (c) (i) NaOH, CH_3_OH/H_2_O, 60 °C, (ii) 1 M HCl, 62%; (d) (i) MnO_2_, DCM, reflux, (ii) CF_3_CO_2_H, anisole, rt, 56%; (e) BBr_3_, DCM, rt, 77%; (f) C_6_H_11_N=C=O, acetone, K_2_CO_3_, reflux, 77%.

The synthesis of target compounds **8**–**9**, **11**–**12**, and **14a**–**b** is described in Scheme 3 through three distinct synthetic routes originating from intermediate **4b**.

**Route 1.** Treatment of **4b** with triethylsilane and TFA simultaneously reduced the secondary alcohol and removed the sulfonamide protecting group. The resulting intermediate was subjected to BBr_3_-mediated demethylation in DCM to afford phenol **7**. Reaction of **7** with benzoic anhydride at 80 °C yielded compound **8**, which was subsequently hydrolyzed under basic conditions (NaOH in methanol/water). This route provides a simple, yet effective, method for synthesizing disubstituted compounds and performing selective ester hydrolysis.

**Route 2.** Oxidation of **4b** with MnO_2_ in DCM afforded the corresponding ketone. Subsequent sulfonamide deprotection using TFA/anisole gave compound **10**, which was further transformed by amidation and BBr_3_-mediated demethylation to provide compound **12**. This sequence showcases a combination of oxidation, deprotection, and selective demethylation to prepare phenolic compounds.

**Route 3.** Reduction of **4b** with triethylsilane/TFA produced intermediate **13**, which was then reacted with benzoic anhydride or cyclohexyl isocyanate to yield derivatives **14a** and **14b**, respectively. This route highlights the flexibility of sulfonamide chemistry, enabling the formation of both amide and urea derivatives.

Compound **16** was synthesized starting from alcohol **4c**. Treatment with triethylsilane and TFA reduced the secondary alcohol to a methylene group and simultaneously removed the methoxymethyl protecting group, affording ester **15**. Subsequent basic hydrolysis (NaOH in methanol/water) produced acid **16** in excellent yield.

**Scheme 4:**
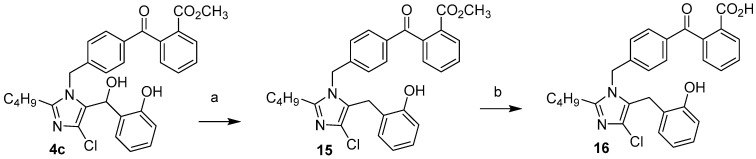
Synthesis of **15**–**16**. Reagents and conditions: (a) (Et)_3_SiH, TFA, rt, 68%; (b) (i) NaOH, CH_3_OH/H_2_O, 60 °C; (ii) 1 M HCl, 88%.

The synthesis of compound **16** begins with the treatment of alcohol **4c** with triethylsilane and TFA at room temperature allowing, the reduction of the alcohol to a methylene group and simultaneously removing the methoxymethyl protection. In the second step, the ester group in the intermediate compound **15** is hydrolyzed under basic conditions (NaOH in methanol/water) at 60 °C, yielding the desired acid **16** in 88% yield. This Scheme 4 illustrates an efficient strategy for reducing alcohols, deprotecting functional groups, and performing ester hydrolysis in a single sequence.

### 2.3. Biological Activity—Structure–Activity Relationships

The title compounds were evaluated for their binding affinities toward AT_1_ and AT_2_ receptors by measuring their ability to displace radiolabeled [^3^H]-angiotensin II ([^3^H]-AII). AT_1_ binding assays were performed using the PLC-PRF-5 human hepatoma cell line [15], whereas AT_2_ assays employed calf cerebellum membranes [16].

Initial structural modifications focused on functionalization of the imidazole core with a biphenylmethylene moiety, a key pharmacophoric feature common to most nonpeptide angiotensin II receptor antagonists. Consistent with the structural requirements observed in irbesartan and losartan analogs, all derivatives retained a butyl substituent on the imidazole ring. Most clinically relevant losartan-type antagonists possess an ionizable acidic function (carboxylate, tetrazole, or acylsulfonamide) located at the biphenyl 2′-position [7,17,18]. This negatively charged group is considered essential for receptor recognition, primarily through electrostatic interaction with positively charged residues such as Lys199 [7,17,18,19].

The binding affinities and functional activities of **4b**–**16** toward the AT_1_ receptor are summarized in Table 1. Clear SAR trends emerged from these data that are summarized in Table 1. Losartan (Ki = 3 nM) served as the reference compound, highlighting the importance of a biphenyl scaffold bearing an ionizable tetrazole moiety for high-affinity AT_1_ receptor binding. Consistent with this pharmacophore, tetrazole-containing derivatives **5** and **6** retained significant affinity (Ki = 25 nM, IC_50_ = 32 nM and Ki = 16 nM, IC_50_ = 20 nM, respectively), with the slightly improved potency of **6** (R_2_ = H) suggesting a modest steric influence near the acidic region. Replacement of the tetrazole with ester **15** resulted in loss of activity, whereas hydrolysis to the corresponding acid **16** partially restored binding (Ki = 251 nM, IC_50_ = 501 nM), confirming the requirement for an ionizable acidic function. Similarly, substitution with a sulfonamide group reduced receptor affinity, as illustrated by the weakly active unsubstituted derivative **7** (Ki = 1000 nM), while bulky substitution (**4b**, Ki = 158 nM, IC_50_ = 794 nM) partially compensated for this decrease.

**Figure 2:**
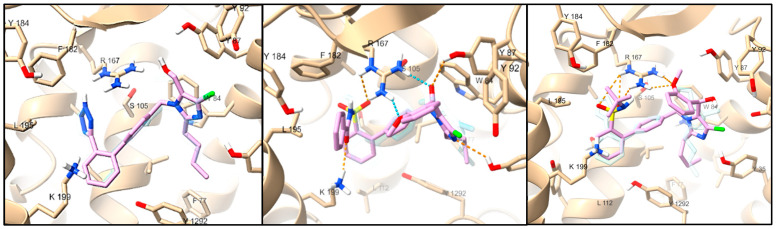
Docking poses within the AT_1_ receptor orthosteric site compared with the X-ray structure of losartan (transparent blue, PDB ID: 8TH4). (**Left**) Docked losartan showing close superposition with the crystallographic pose. (**Middle**) **11**, exhibiting good overlap of both the biphenyl scaffold and the imidazole core. The benzoyl-sulfonamide moiety establishes an extended network of polar and hydrophobic contacts involving residues Arg167, Lys199, Tyr184, and Phe182, while preserving the ionizable character typically associated with the tetrazole group of losartan. Replacement of the benzylic alcohol by a methoxyphenyl substituent combined with a carbonyl linker modifies local geometry and enables additional stabilizing interactions, notably with Tyr87 and Tyr92. (**Right**) **4b**, bearing a secondary alcohol, is predicted to lose key hydrogen-bond interactions with Arg167 and Tyr87. In addition, a slight shift of the biphenyl scaffold is observed, which may contribute to its reduced activity.

Molecular docking studies using the antagonist-bound AT_1_ receptor structure (PDB: 8TH4) indicated that the compounds adopt binding poses comparable to losartan, consistent with the SAR observations (Figure 2).

Docking analysis of **4b** suggests that the secondary alcohol linker induces subtle geometric differences, resulting in a slightly displaced pose within the orthosteric pocket and less optimal alignment of the biphenyl scaffold and sulfonamide pharmacophore (Figure 2). This shift is consistent with steric interference involving the nearby residue Tyr92, an effect not observed for the methylene-linked analog **5**.

Further introduction of an aromatic substituent on the sulfonamide nitrogen produced a pronounced increase in receptor affinity. Comparison of the unsubstituted sulfonamide **13** (Ki = 501 nM, IC_50_ = 1000 nM) with N-benzoyl derivative **14a** (Ki = 16 nM, IC_50_ = 32 nM) and the urea homolog **14b** (Ki = 20 nM, IC_50_ = 40 nM) highlights the favorable contribution of aromatic functionalization. This effect is consistent with docking observations, which indicate that the benzoyl group enhances hydrophobic complementarity and enables stabilizing aromatic interactions, notably involving residues Tyr184 and Phe182. These interactions likely contribute to improved pocket occupancy and ligand stabilization, with a preference for aromatic moiety, as seen with the slight decrease for the saturated **14b**.

Variation in the linker oxidation state significantly affected receptor binding, as seen with **4b**. Exploring this phenomenon, replacement of the methylene linker with a carbonyl group resulted in improved affinity, as illustrated by **10** (Ki = 25 nM, IC_50_ = 50 nM) and **11** (Ki = 5 nM, IC_50_ = 10 nM) compared with their methylene analogs **13** and **14a**. Docking analysis suggests that the carbonyl linker promotes a more favorable orientation of the phenyl/biphenyl system within the orthosteric pocket (Figure 1). In particular, the ketone functionality appears to reduce steric interference near Tyr92, while enabling improved alignment of the aromatic scaffold. Additionally, the carbonyl oxygen may participate in stabilizing polar interactions, including potential hydrogen bonding with residues such as Arg167 and Tyr87, contributing to enhanced ligand recognition.

Finally, modification of the R_2_ substituent further modulated receptor affinity. Conversion of the methoxy group to the corresponding phenol (**11** vs. **12**, Ki = 5 nM vs. 10 nM) resulted in a modest decrease in potency. Docking observations suggest that the phenolic hydroxyl group introduces unfavorable interactions, including potential repulsion or suboptimal contacts near Tyr87. Similarly, incorporation of bulky R_2_ substituents (**8** vs. **14a**) reduced receptor affinity, consistent with steric crowding effects involving the nearby residue Tyr92.

The structure–activity trends observed here suggest that specific functional groups such as sulfonamide, tetrazole and aromatic substituents are critical for improving the inhibitory activity of these compounds at the AT_1_ receptor. These findings underscore the importance of fine-tuning substituents at positions R_1_ and R_2_ to achieve optimal receptor affinity.

Importantly, all compounds exhibited negligible AT_2_ receptor affinity (percentage of inhibition below 20% at 10^−5^ M), demonstrating a high degree of selectivity for the AT_1_ receptor, which is a desirable feature for drugs targeting AT_1_-mediated pathways. In silico predictions were run on the three best overall compounds, **11**, **12** and **14a**, with the Deep-PK API [20] and resulted in similar properties compared to losartan, with drug-like properties (Table S1). Noticeably, our compounds, without the tetrazole moiety, replaced with substituted sulfonamide, present slightly better predicted oral bioavailability and slightly less hepatic metabolism.

## 3. Discussion

A series of novel losartan-inspired imidazole derivatives were identified as potent and selective AT_1_ receptor antagonists. SAR analysis highlighted the key roles of the ionizable tetrazole and N-acyl sulfonamide groups as well as the favorable impact of aromatic substitution and linker oxidation state. Docking studies confirmed binding modes consistent with losartan, providing structural insight into the observed pharmacological trends, explaining the contribution of key substituents to receptor binding.

These findings expand the chemical space of AT_1_ antagonists and establish a strong foundation for further optimization. Additional functional and pharmacokinetic studies will be essential to advance these compounds toward potential therapeutic applications in diseases with renin–angiotensin system dysregulation.

## 4. Experimental Section

### 4.1. Chemistry

Chemicals and solvents were obtained from commercial sources and used without further purification unless otherwise noted. Reactions were monitored by TLC performed on Macherey-Nagel Alugram^®^ Sil 60/UV254 sheets (thickness 0.2 mm, Macherey-Nagel GmbH & Co. KG, Düren, Germany). Purification of products was carried out by recrystallization or column chromatography. Column chromatography was carried out using Macherey-Nagel silica gel (230–400 mesh, Macherey-Nagel GmbH & Co. KG, Düren, Germany). Melting points were determined on a Büchi SMP-20 capillary apparatus (Büchi SARL, Villebon sur Yvette, France) and are uncorrected. NMR spectra were recorded on a Bruker DRX 300 spectrometer (Division Biospin, Wissembourg, France) operating at 300 MHz for ^1^H and 75 MHz for ^13^C). Chemical shifts are expressed in ppm relative to tetramethylsilane. Chemical shifts are reported as position (δ in ppm), multiplicity (s = singlet, d = doublet, t = triplet, q = quartet, dd = double doublet, br = broad, and m = multiplet), coupling constant (*J* in Hz), relative integral, and assignment. Infrared spectra were obtained on a Perkin-Elmer FT-IR S1000 on KBr paths. Mass spectra were recorded with decimal precision using a Waters AcQuity UPLC I-Class with UV detection (PDA) and an electrospray mode (ESI) (Waters Corporation, Milford, MA, USA). UPLC-MS Waters system was equipped with a UPLC I SMP MGR-FTN sample manager, an ACQUITY UPLC I-Class eK photodiode array detector (210–400 nm), and an ACQUITY QDa (Performance) detector (scan 50–1250) (Waters Corporation, Milford, MA, USA). Acquity BEH C18 column (50 mm × 2.1 mm, 1.7 μm, Waters) was used. The injection volume was 0.5 μL. A mixture of water and acetonitrile was used as mobile phase in gradient elution. The pH of the mobile phase was adjusted with HCOOH and NH_3_ (aq) to form a buffer solution at pH 3.8. The analysis time was 5 min (at a flow rate of 600 μL/min), 10 min (at a flow rate of 600 μL/min), or 30 min (at a flow rate of 600 μL/min). Unless otherwise specified, the purity of evaluated compounds was judged to be >95% as determined by UPLC-UV-MS system. HRMS analysis was performed on a LC–MS system equipped with a LCT Premier XE mass spectrometer (Waters) using an XBridge C18 column (50 mm × 4.6 mm, 3.5 μm, Waters). A gradient starting from 98% H_2_O 5 mM ammonium formate pH 3.8 and reaching 100% CH_3_CN 5 mM ammonium formate pH 3.8 within 3 min at a flow rate of 1 mL/min was used. NMR, LC-MS and HRMS spectra are provided as Supplementary Materials.

#### 4.1.1. General Procedure for the N-Alkylation Reaction

Potassium carbonate (30 mmol) was added in portions to a solution of compound **1** (10 mmol) in anhydrous DMF (60 mL). The appropriate alkyl bromide (**2a**–**c**, 10.2 mmol) was subsequently introduced into the reaction mixture, which was stirred at 70 °C for 3 h. Following the reaction, water (150 mL) was added to the mixture, resulting in a suspension that was extracted twice with Et_2_O. The organic phase was washed with water, dried over MgSO_4_, and concentrated under reduced pressure. The resulting solid was purified by recrystallization from the suitable solvent.

#### 4.1.2. Synthesis of 2-Butyl-4-chloro-1-((2′-(1-trityl-1H-tetrazol-5-yl)-[1,1′-biphenyl]-4-yl)methyl)-1H-imidazole-5-carbaldehyde (**3a**)

White solid (77%); mp 127–129 °C (CH_3_CN). ^1^H NMR (300 MHz, DMSO-*d*_6_) δ 9.67 (s, 1H), 7.79 (d, *J* = 6.8 Hz, 1H), 7.74–7.50 (m, 2H), 7.49–7.26 (m, 10H), 7.69 (d, *J* = 8.1 Hz, 2H), 6.95 (d, *J* = 8.1 Hz, 2H), 6.85 (d, *J* = 7.5 Hz, 6H), 5.53 (s, 2H), 2.65 (t, *J* = 7.2 Hz, 2H), 1.47 (quint, *J* = 7.8 Hz, 2H), 1.16 (sext, *J* = 7.3 Hz, 2H), 0.76 (t, *J* = 7.4 Hz, 3H). ^13^C NMR (75 MHz, DMSO-*d*_6_) δ 178.3, 163.8, 154.7, 141.5, 141.3, 140.8, 139.6, 134.9, 130.7, 130.5, 130.3, 129.6, 129.3, 128.3, 126.6, 126.1, 124.4, 82.7, 47.5, 28.9, 25.9, 22.2, 14.0. LCMS tr = 2.78 min, *m*/*z* calc for [M+H]^+^: 663, found: 663.

#### 4.1.3. Synthesis of N-tert-Butyl-2-[4-[(2-butyl-4-chloro-5-formyl-imidazol-1-yl)methyl]phenyl] Benzene Sulfonamide (**3b**)

White solid (78%); mp 117–119 °C (Isopropyl ether). ^1^H NMR (500 MHz, DMSO-*d*_6_) δ 9.72 (s, 1H), 8.03 (dd, *J* = 7.9, 1.5 Hz, 1H), 7.61 (td, *J* = 7.5, 1.5 Hz, 1H), 7.59–7.53 (m, 1H), 7.38 (d, *J* = 7.9 Hz, 2H), 7.27 (dd, *J* = 7.5, 1.4 Hz, 1H), 7.13 (d, *J* = 7.9 Hz, 2H), 6.57 (s, 1H), 5.65 (s, 2H), 2.68 (t, *J* = 7.6 Hz, 2H), 1.57 (quint, *J* = 7.6 Hz, 2H), 1.29 (hex, *J* = 7.5 Hz, 2H), 0.95 (s, 9H), 0.84 (t, *J* = 7.3 Hz, 3H). ^13^C NMR (126 MHz, DMSO-*d*_6_) δ 178.0, 154.4, 142.1, 141.0, 139.5, 139.2, 135.3, 132.5, 131.8, 129.7, 128.0, 127.8, 125.8, 124.0, 53.4, 47.2, 29.3, 28.5, 25.5, 21.7, 13.6. LCMS tr = 2.50 min, *m*/*z* calc for [M+H]^+^: 488, found: 488; [M-H]^−^: 486, found: 486.

#### 4.1.4. Synthesis of Methyl 2-(4-((2-Butyl-4-chloro-5-formyl-1H-imidazol-1-yl)methyl)benzoyl) Benzoate (**3c**)

White solid (77%); mp 98–100 °C (MeOH). ^1^H NMR (300 MHz, DMSO-*d*_6_): δ 9.72 (s, 1H), 8.12 (dd, *J* = 1.2, 8.2 Hz, 1H), 7.75–7.70 (m, 2H), 7.65 (d, *J* = 8.2 Hz, 2H), 7.45 (dd, *J* = 7.4, 1.20 Hz, 1H), 7.22 (d, *J* = 8.2 Hz, 2H), 5.70 (s, 2H), 3.56 (s, 3H), 2.65 (t, *J* = 7.3 Hz, 2H), 1.62 (qt, *J* = 7.3 Hz, 2H), 1.30 (sext, *J* = 3.0 Hz, 2H), 0.80 (t, *J* = 7.3 Hz, 3H). ^13^C NMR (75 MHz, DMSO-*d*_6_): δ 196.3, 189.1, 168.2, 155.9, 141.2, 140.6, 136.0, 135.4, 133.8, 132.7, 132.4, 132.2, 130.2, 128.4, 127.8, 127.2, 51.5, 47.3, 30.8, 25.3, 22.3, 14.2. LCMS tr = 2.40 min, *m*/*z* calc for [M+H]^+^: 439, found: 439; [M-H]^−^: 437, found: 437.

#### 4.1.5. General Procedure for the Grignard Reaction

To a suspension of magnesium turnings (160 mg, 6 mmol) in dry THF (40 mL) under an argon atmosphere was added, dropwise, a solution of the bromine derivative (5.5 mmol) in dry THF (30 mL). The mixture was stirred at reflux for 1 h. Subsequently, the obtained solution was added dropwise to a solution of the desired carbaldehyde (5 mmol) in dry THF (30 mL). The resulting solution was stirred for an additional 3 h. The reaction was quenched with a saturated solution of NH_4_Cl (20 mL). The reaction mixture was then extracted with ethyl acetate, washed with brine, and dried over MgSO_4_. The organic layer was evaporated under reduced pressure, and the crude product was triturated in Et_2_O, filtered and recrystallized from the appropriate solvent.

#### 4.1.6. Synthesis of N-tert-Butyl-2-[4-[[2-butyl-4-chloro-5-[hydroxy-(2-methoxyphenyl)methyl] imidazol-1-yl]methyl]phenyl] Benzene Sulfonamide (**4b**)

White solid (75%); mp 167–168 °C (CH_3_CN). ^1^H NMR (300 MHz, DMSO-*d*_6_) δ 8.04 (dd, *J* = 7.8, 1.5 Hz, 1H), 7.70–7.47 (m, 3H), 7.33–7.22 (m, 3H), 7.22–7.11 (m, 1H), 6.97–6.81 (m, 4H), 6.41 (s, 1H), 5.96 (s, 2H), 5.28 (s, 2H), 3.68 (s, 3H), 2.37 (t, *J* = 7.6 Hz, 2H), 1.47 (quint, *J* = 8.2 Hz, 2H), 1.22 (sext, *J* = 7.3 Hz, 2H), 0.94 (s, 9H), 0.79 (t, *J* = 7.3 Hz, 3H). ^13^C NMR (75 MHz, DMSO-*d*_6_) δ 155.8, 146.7, 142.0, 139.6, 138.5, 136.2, 132.6, 131.8, 129.4, 129.2, 128.2, 128.0, 127.7, 126.7, 126.2, 125.3, 124.8, 119.9, 110.4, 60.7, 55.3, 53.3, 46.4, 29.3, 28.7, 25.8, 21.7, 13.7. LCMS tr = 2.44 min, *m*/*z* calc for [M+H]^+^: 596, found: 596; [M-H]^−^: 594, found: 594. HRMS *m*/*z* calc for C_32_H_39_ClN_3_O_4_S [M+H]^+^ 596.2350, found 596.2349.

#### 4.1.7. Synthesis of Methyl 2-(4-((2-Butyl-4-chloro-5-(hydroxy(2-(methoxymethoxy)phenyl)methyl)-1H-imidazol-1-yl)methyl)benzoyl)benzoate (**4c**)

White solid (73%); mp 144–146 °C (EtOH). ^1^H NMR (300 MHz, DMSO-*d*_6_) δ 7.99 (dd, *J* = 7.6, 1.4 Hz, 1H), 7.77 (td, *J* = 7.4, 1.4 Hz, 1H), 7.69 (td, *J* = 7.6, 1.4 Hz, 1H), 7.56 (dd, *J* = 7.7, 1.7 Hz, 1H), 7.49–7.38 (m, 3H), 7.13–7.02 (m, 1H), 6.94–6.85 (m, 3H), 6.81 (t, *J* = 7.5 Hz, 1H), 6.06 (d, *J* = 4.6 Hz, 1H), 5.94 (d, *J* = 4.2 Hz, 1H), 5.38 (d, *J* = 17.9 Hz, 1H), 5.27 (d, *J* = 17.8 Hz, 1H), 5.14 (d, *J* = 6.9 Hz, 1H), 5.04 (d, *J* = 6.9 Hz, 1H), 3.56 (s, 3H), 3.11 (s, 3H), 2.34–2.25 (m, 2H), 1.47–1.30 (m, 2H), 1.15 (hept, *J* = 7.3 Hz, 2H), 0.73 (t, *J* = 7.3 Hz, 3H). ^13^C NMR (75 MHz, DMSO-*d*_6_) δ 195.6, 165.9, 152.9, 146.7, 142.6, 141.0, 135.4, 132.8, 130.2, 129.7, 129.7, 128.9, 128.7, 128.0, 127.8, 126.7, 126.2, 125.9, 125.3, 120.7, 112.6, 92.8, 60.7, 55.3, 52.2, 46.5, 28.8, 25.6, 21.6, 13.5. LCMS tr = 2.25 min, *m*/*z* calc for [M+H]^+^: 577, found: 577; *m*/*z* calc for [M-H]^−^: 575, found: 575. HRMS *m*/*z* calc for C_32_H_34_ClN_2_O_6_ [M+H]^+^ 577.2105, found 577.2127.

#### 4.1.8. Synthesis of 5-(4′-((2-Butyl-4-chloro-5-(2-methoxybenzyl)-1H-imidazol-1-yl)methyl)-[1,1′-biphenyl]-2-yl)-1H-tetrazole (**5**)

Trifluoroacetic acid (4.5 mL, 30 mmol) was added dropwise to a solution of compound **4a** (1.54 g, 2 mmol) in DCM (60 mL), followed by the dropwise addition of Et_3_SiH (3.23 mL, 20 mmol). The resulting reaction mixture was stirred at ambient temperature for 18 h. After completion of the reaction, the mixture was concentrated under reduced pressure, and water was added to the residue. The resulting precipitate was collected by filtration, washed with n-hexane, and dried to afford the desired product. The solid was further purified by recrystallization in EtOH. White solid (85%); mp 176–178 °C (EtOH). ^1^H NMR (300 MHz, DMSO-*d*_6_) δ 9.62 (br s, 1H), 7.70–7.54 (m, 2H), 7.62 (m, 1H), 7.54 (m,1H), 7.10–6.92 (m, 3H), 6.82 (m, 2H), 6.75 (m, 2H), 6.64 (m, 1H), 5.05 (s, 2H), 3.72 (s, 2H), 3.78 (s, 3H), 2.42 (t, *J* = 7.6 Hz, 2H), 1.45 (quint, *J* = 7.2 Hz, 2H), 1.22 (sext, *J* = 7.2 Hz, 2H), 0.80 (t, *J* = 7.3 Hz, 3H). ^13^C NMR (75 MHz, DMSO-*d*_6_) δ 155.5, 154.2, 146.4, 141.0, 137.4, 134.8, 131.2, 130.5, 128.1, 128.9, 127.9, 127.6, 125.8, 125.4, 124.7, 124.1, 123.7, 123.4, 119.2, 115.1, 57.8, 46.4, 29.0, 25.8, 22.3, 21.7, 13.5. LCMS tr = 2.20 min, *m*/*z* calc for [M+H]^+^: 513, found: 513; [M-H]^−^: 511, found: 511.

#### 4.1.9. Synthesis of 2-((1-((2′-(1H-Tetrazol-5-yl)-[1,1′-biphenyl]-4-yl)methyl)-2-butyl-4-chloro-1H-imidazol-5-yl)methyl)phenol (**6**)

To a stirred solution of compound **5** (0.51 g, 1 mmol) in anhydrous DCM (50 mL), BBr_3_ (0.14 mL, 1.2 mmol) was added, dropwise, at 0 °C under an inert nitrogen atmosphere. The reaction mixture was allowed to warm to room temperature and stirred for 2 h. The reaction mixture was quenched by the slow addition of water. The crude product was purified by recrystallization in CH_3_CN. White solid (65%); mp 223–225 °C (CH_3_CN). ^1^H NMR (300 MHz, DMSO-*d*_6_) δ 9.62 (br s, 1H), 7.71–7.64 (m, 2H), 7.60 (m, 1H), 7.51 (m,1H), 7.03–6.97 (m, 3H), 6.82 (m, 2H), 6.77 (m, 2H), 6.66 (m, 1H), 5.07 (s, 2H), 3.74 (s, 2H), 2.45 (t, *J* = 7.6 Hz, 2H), 1.45 (quint, *J* = 7.2 Hz, 2H), 1.24 (sext, *J* = 7.2 Hz, 2H), 0.79 (t, *J* = 7.3 Hz, 3H). ^13^C NMR (75 MHz, DMSO-*d*_6_) δ 154.4, 146.5, 141.0, 138.3, 136.0, 131.1, 130.6, 129.1, 128.9, 127.8, 127.5, 125.9, 124.7, 124.4, 123.8, 123.4, 119.1, 115.0, 46.3, 29.0, 25.9, 22.2, 21.6, 13.6. LCMS tr = 2.01 min, *m*/*z* calc for [M+H]^+^: 499, found: 499; [M-H]^−^: 497, found: 497. HRMS *m*/*z* calc for C_28_H_28_ClN_6_O [M+H]^+^ 499.2013, found 499.2013.

#### 4.1.10. Synthesis of 4′-((2-Butyl-4-chloro-5-(2-hydroxybenzyl)-1H-imidazol-1-yl)methyl)-[1,1′-biphenyl]-2-sulfonamide (**7**)

TFA (4.5 mL, 30 mmol) was added dropwise to a solution of compound **4b** (1.16 g, 2 mmol) in DCM (60 mL), followed by the dropwise addition of Et_3_SiH (3.23 mL, 20 mmol). The resulting reaction mixture was stirred at room temperature for 18 h. After completion of the reaction, the mixture was concentrated under reduced pressure, and water was added to the residue. The resulting precipitate was collected by filtration, washed with n-hexane, dried, and used for the next step without further purification. The solid was dissolved in anhydrous DCM (50 mL), and BBr_3_ (0.28 mL, 1.5 mmol) was added dropwise at 0 °C under an inert nitrogen atmosphere. The reaction mixture was allowed to warm to room temperature and stirred for 4 h. The reaction mixture was quenched by the slow addition of water. The crude product was collected by filtration, dried, and purified by recrystallization in CH_3_CN. White solid (72%); mp 155–157 °C (CH_3_CN). ^1^H NMR (500 MHz, DMSO-*d*_6_) δ 9.69 (br s, 1H), 8.01 (dd, *J* = 7.9, 1.5 Hz, 1H), 7.61 (td, *J* = 7.5, 1.5 Hz, 1H), 7.56 (td, *J* = 7.7, 1.5 Hz, 1H), 7.30 (d, *J* = 8.0 Hz, 2H), 7.25 (dd, *J* = 7.5, 1.5 Hz, 1H), 7.18 (s, 2H), 7.01 (td, *J* = 7.6, 1.8 Hz, 1H), 6.91 (d, *J* = 8.0 Hz, 2H), 6.80–6.76 (m, 2H), 6.70 (t, *J* = 7.2 Hz, 1H), 5.12 (s, 2H), 3.78 (s, 2H), 2.53 (t, *J* = 7.6 Hz, 2H), 1.49 (quint, *J* = 7.6 Hz, 2H), 1.25 (sext, *J* = 7.4 Hz, 2H), 0.80 (t, *J* = 7.3 Hz, 3H). ^13^C NMR (126 MHz, DMSO-*d*_6_) δ 154.5, 146.7, 142.3, 139.4, 139.2, 135.9, 132.4, 131.6, 129.6, 128.9, 127.8, 127.4, 125.3, 125.1, 124.5, 124.0, 119.3, 115.1, 46.5, 29.2, 26.1, 22.3, 21.8, 13.7.

LCMS tr = 2.12 min, *m*/*z* calc for [M+H]^+^: 510, found: 510; [M-H]^−^: 508, found: 508. HRMS *m*/*z* calc for C_27_H_29_ClN_3_O_3_S [M+H]^+^ 510.1618, found 510.1626.

#### 4.1.11. Synthesis of 2-((1-((2′-(N-Benzoylsulfamoyl)-[1,1′-biphenyl]-4-yl)methyl)-2-butyl-4-chloro-1H-imidazol-5-yl)methyl) Phenyl Benzoate (**8**)

Benzoic anhydride (0.57 mL, 3 mmol) was added dropwise to a stirred solution of compound **7** (0.51 g, 1 mmol) in pyridine (10 mL) at room temperature. The reaction mixture was heated to 80 °C and maintained at this temperature for 4 h. After completion, the mixture was allowed to cool to room temperature and was then transferred to an ice/water bath (100 mL). The resulting precipitate was isolated by filtration and washed with water and Et_2_O. The crude product was recrystallized from EtOH to yield compound **8** as a white solid in 85% yield. White solid (85%); mp 148–150 °C (EtOH). ^1^H NMR (300 MHz, DMSO) δ 8.10–7.98 (m, 3H), 7.75–7.60 (m, 3H), 7.53 (t, *J* = 7.7 Hz, 2H), 7.44–7.14 (m, 10H), 6.96–6.82 (m, 2H), 6.66 (d, *J* = 7.9 Hz, 2H), 4.91 (s, 2H), 3.73 (s, 2H), 2.56 (m, 2H), 1.56 (quint, *J* = 7.5 Hz, 2H), 1.30 (hex, *J* = 7.4 Hz, 2H), 0.86 (t, *J* = 7.3 Hz, 3H). ^13^C NMR (75 MHz, CDCl_3_) δ 164.3, 148.5, 147.0, 143.4, 140.5, 139.0, 134.1, 134.0, 131.3, 129.8, 129.8, 129.5, 129.5, 129.3, 129.2, 129.1, 128.9, 128.5, 128.2, 127.9, 127.0, 126.4, 125.9, 124.2, 122.8, 122.3, 46.7, 29.1, 25.9, 23.2, 21.7, 13.8. LCMS tr = 2.40 min, *m*/*z* calc for [M+H]^+^: 718, found: 718; [M-H]^−^: 716, found: 716. HRMS *m*/*z* calc for C_41_H_37_ClN_3_O_5_S [M+H]^+^ 718.2142, found 718.2165.

#### 4.1.12. Synthesis of N-((4′-((2-Butyl-4-chloro-5-(2-hydroxybenzyl)-1H-imidazol-1-yl)methyl)-[1,1′-biphenyl]-2-yl)sulfonyl) Benzamide (**9**)

Sodium hydroxide (NaOH, 0.12 g, 3 mmol) was dissolved in water (30 mL) and added to a stirred solution of compound **8** (0.72 g, 1 mmol) in methanol (MeOH, 10 mL). The reaction mixture was heated to reflux and stirred for 2 h. After the reaction was complete, the solvent was removed under reduced pressure, and water was added to the residue. The aqueous solution was then acidified with 1 M HCl. The resulting precipitate was isolated by filtration and washed with water and Et_2_O. The crude product was purified by recrystallization from ethanol (EtOH). White solid (62%); mp 267–269 °C (EtOH). ^1^H NMR (300 MHz, DMSO-*d*_6_) δ 12.08 (br s, 1H), 9.88 (br s, 1H), 8.17 (dd, *J* = 7.9, 1.5 Hz, 1H), 7.79–7.54 (m, 5H), 7.40 (t, *J* = 7.7 Hz, 2H), 7.26 (dd, *J* = 7.4, 1.6 Hz, 1H), 7.22 (d, *J* = 8.2 Hz, 2H), 7.05 (td, *J* = 7.6, 1.8 Hz, 1H), 6.93 (d, *J* = 8.0 Hz, 2H), 6.92–6.78 (m, 2H), 6.71 (td, *J* = 7.4, 1.2 Hz, 1H), 5.28 (s, 2H), 3.82 (s, 2H), 2.71 (t, *J* = 7.7 Hz, 2H), 1.57 (quint, *J* = 7.7 Hz, 2H), 1.27 (sext, *J* = 7.4 Hz, 2H), 0.83 (t, *J* = 7.3 Hz, 3H). ^13^C NMR (75 MHz, DMSO-*d*_6_) δ 165.1, 154.8, 147.0, 139.8, 138.1, 137.7, 135.1, 133.3, 133.2, 132.7, 131.1, 129.5, 129.3, 128.4, 128.4, 128.3, 128.0, 126.2, 125.6, 122.7, 121.0, 119.2, 115.2, 47.0, 28.7, 25.3, 22.7, 21.7, 13.6. LCMS tr = 2.15 min, *m*/*z* calc for [M+H]^+^: 614, found: 614; [M-H]^−^: 612, found: 612. HRMS *m*/*z* calc for C_34_H_33_ClN_3_O_4_S [M+H]^+^ 614.1880, found 614.1887.

#### 4.1.13. Synthesis of 4′-((2-Butyl-4-chloro-5-(2-methoxybenzoyl)-1H-imidazol-1-yl)methyl)-[1,1′-biphenyl]-2-sulfonamide (**10**)

MnO_2_ (0.35 g, 4 mmol) was added to a solution of the compound **4b** (0.6 g, 1 mmol) in DCM. The mixture was stirred under reflux for 4 h, and then filtered. Trifluoroacetic acid (5 mL) and anisole (0.1 mL, 1 mmol) were added, and the solution was stirred for 3 h and evaporated. The residue was triturated with Et_2_O and filtered. The precipitate was recrystallized from EtOH. White solid (56%); mp 147–148 °C (EtOH). ^1^H NMR (300 MHz, DMSO-*d*_6_) δ 8.03 (dd, *J* = 7.4, 1.9 Hz, 1H), 7.65–7.46 (m, 3H), 7.39 (d, *J* = 8.1 Hz, 2H), 7.29–7.23 (m, 2H), 7.20–7.08 (m, 5H), 7.04 (t, *J* = 7.4 Hz, 1H), 5.72 (s, 2H), 3.74 (s, 3H), 2.68 (t, *J* = 7.6 Hz, 2H), 1.56 (quint, *J* = 7.6 Hz, 2H), 1.30 (sext, *J* = 7.1 Hz, 2H), 0.84 (t, *J* = 7.3 Hz, 3H). ^13^C NMR (75 MHz, DMSO-*d*_6_) δ 184.4, 166.5, 157.0, 153.1, 142.3, 139.4, 139.2, 136.5, 135.8, 132.5, 131.4, 129.5, 129.1, 128.6, 127.7, 127.3, 125.5, 120.7, 111.7, 55.8, 47.5, 28.6, 25.7, 21.7, 13.6. LCMS tr = 2.28 min, *m*/*z* calc for [M+H]^+^: 538, found: 538; [M-H]^−^: 536, found: 536. HRMS *m*/*z* calc for C_28_H_29_ClN_3_O_4_S [M+H]^+^ 538.1567, found 538.1580.

#### 4.1.14. Synthesis of N-((4′-((2-Butyl-4-chloro-5-(2-methoxybenzoyl)-1H-imidazol-1-yl)methyl)-[1,1′-biphenyl]-2-yl)sulfonyl) Benzamide (**11**)

This compound was prepared following the same procedure as compound **8**, using compound **10** (0.65 g, 1.2 mmol) and benzoic anhydride (0.27 mL, 1.44 mmol) in pyridine (10 mL). Recrystallization from EtOH yielded compound **11**. White solid (82%); mp 169–170 °C (EtOH). ^1^H NMR (500 MHz, DMSO-*d*_6_) δ 12.08 (br s, 1H), 8.17 (dd, *J* = 8.0, 1.4 Hz, 1H), 7.72 (td, *J* = 7.5, 1.5 Hz, 1H), 7.66 (td, *J* = 7.7, 1.5 Hz, 1H), 7.59–7.54 (m, 2H), 7.55–7.46 (m, 2H), 7.36 (t, *J* = 7.7 Hz, 2H), 7.32–7.25 (m, 4H), 7.10 (d, *J* = 8.4 Hz, 3H), 7.05 (t, *J* = 7.4 Hz, 1H), 5.73 (s, 2H), 3.65 (s, 3H), 2.66 (t, *J* = 7.6 Hz, 2H), 1.60 (quint, *J* = 7.6 Hz, 2H), 1.31 (sext, *J* = 7.4 Hz, 2H), 0.86 (t, *J* = 7.4 Hz, 3H). ^13^C NMR (126 MHz, DMSO-*d*_6_) δ 184.5, 165.1, 157.0, 153.1, 140.0, 137.8, 137.6, 136.6, 136.3, 133.2, 133.0, 132.7, 132.6, 131.1, 129.7, 129.4, 129.1, 128.7, 128.3, 128.2, 128.1, 125.7, 125.6, 120.7, 111.7, 55.7, 28.5, 25.7, 21.7, 13.7. LCMS tr = 2.31 min, *m*/*z* calc for [M+H]^+^: 642, found: 642; [M-H]^−^: 640, found: 640. HRMS *m*/*z* calc for C_35_H_33_ClN_3_O_5_S [M+H]^+^ 642.1829, found 642.1796.

#### 4.1.15. Synthesis of N-((4′-((2-Butyl-4-chloro-5-(2-hydroxybenzoyl)-1H-imidazol-1-yl)methyl)-[1,1′-biphenyl]-2-yl)sulfonyl) Benzamide (**12**)

This compound was prepared following the same procedure as compound **6**, using compound **11** (0.42 g, 0.65 mmol) and BBr_3_ (0.092 mL, 0.98 mmol). Recrystallization from EtOH gave compound **12**. White solid (77%); mp 189–191 °C (EtOH). ^1^H NMR (500 MHz, DMSO) δ 12.09 (br s, 1H), 10.17 (br s, 1H), 8.16 (dd, *J* = 8.0, 1.4 Hz, 1H), 7.71 (td, *J* = 7.5, 1.4 Hz, 1H), 7.65 (td, *J* = 7.7, 1.4 Hz, 1H), 7.59–7.50 (m, 3H), 7.43–7.34 (m, 3H), 7.33 (m, 1H), 7.28–7.21 (m, 3H), 7.16–7.10 (m, 2H), 6.95 (m, 2H), 5.64 (s, 2H), 2.65 (t, *J* = 7.3 Hz, 2H), 1.59 (quint, *J* = 7.6 Hz, 2H), 1.30 (sext, *J* = 7.4 Hz, 2H), 0.85 (t, *J* = 7.3 Hz, 3H). ^13^C NMR (126 MHz, DMSO) δ 186.2, 165.1, 156.9, 156.5, 153.6, 152.6, 140.0, 137.8, 137.6, 136.3, 135.5, 133.2, 133.1, 132.7, 131.2, 130.2, 129.8, 129.3, 128.3, 128.2, 128.1, 126.4, 125.9, 123.3, 119.2, 116.4, 47.3, 28.5, 25.7, 21.7, 13.7. LCMS tr = 2.29 min, *m*/*z* calc for [M+H]^+^: 628, found: 628; [M-H]^−^: 626, found: 626. HRMS *m*/*z* calc for C_34_H_31_ClN_3_O_5_S [M+H]^+^ 628.1673, found 628.1703.

#### 4.1.16. Synthesis of 4′-((2-Butyl-4-chloro-5-(2-methoxybenzyl)-1H-imidazol-1-yl)methyl)-[1,1′-biphenyl]-2-sulfonamide (**13**)

This compound was prepared following the same procedure as compound **5**, using compound **4b** (1.2 g, 2 mmol), Et_3_SiH (3.23 mL, 20 mmol) and TFA (4.46 mL, 60 mmol). Recrystallization from CH_3_CN gave compound **13.** White solid (52%); mp 182–184 °C (CH_3_CN). ^1^H NMR (500 MHz, DMSO-*d*_6_) δ 8.02 (dd, *J* = 7.9, 1.4 Hz, 1H), 7.61 (t, *J* = 7.5 Hz, 1H), 7.57 (t, *J* = 7.5 Hz, 1H), 7.29 (d, *J* = 8.0 Hz, 2H), 7.25 (d, *J* = 7.3, 1H), 7.21–7.15 (m, 3H), 6.92 (d, *J* = 8.2 Hz, 1H), 6.90–6.79 (m, 4H), 5.11 (s, 2H), 3.79 (s, 2H), 3.75 (s, 3H), 2.54 (t, *J* = 2.5 Hz, 2H), 1.52 (quint, *J* = 7.6 Hz, 2H), 1.27 (sext, *J* = 7.4 Hz, 2H), 0.82 (t, *J* = 7.3 Hz, 3H). ^13^C NMR (126 MHz, DMSO-*d*_6_) δ 156.5, 146.7, 142.2, 139.4, 139.0, 135.7, 132.4, 131.5, 129.5, 128.7, 127.9, 127.7, 127.3, 125.4, 125.3, 125.0, 123.9, 120.4, 110.7, 55.3, 46.4, 29.1, 26.0, 22.5, 21.7, 13.7. LCMS tr = 2.32 min, *m*/*z* calc for [M+H]^+^: 524, found: 524; [M-H]^−^: 522, found: 522. HRMS *m*/*z* calc for C_28_H_31_ClN_3_O_3_S [M+H]^+^ 524.1775, found 524.1777.

#### 4.1.17. Synthesis of N-((4′-((2-Butyl-4-chloro-5-(2-methoxybenzyl)-1H-imidazol-1-yl)methyl)-[1,1′-biphenyl]-2-yl)sulfonyl) Benzamide (**14a**)

This compound was prepared following the same procedure as compound **8**, using compound **13** (0.65 g, 1.2 mmol) and benzoic anhydride (0.27 mL, 1.44 mmol). Recrystallization from CH_3_CN gave compound **14a.** White solid (76%); mp 204–206 °C (CH_3_CN). ^1^H NMR (300 MHz, DMSO-*d*_6_) δ 12.02 (s, 1H), 8.16 (dd, *J* = 7.8, 1.5 Hz, 1H), 7.72 (td, *J* = 7.5, 1.6 Hz, 1H), 7.65 (td, *J* = 7.6, 1.6 Hz, 1H), 7.62–7.52 (m, 3H), 7.42–7.34 (m, 2H), 7.26 (dd, *J* = 7.5, 1.5 Hz, 1H), 7.23–7.14 (m, 3H), 6.93 (d, *J* = 8.2 Hz, 1H), 6.88–6.78 (m, 4H), 5.09 (s, 2H), 3.78 (s, 2H), 3.76 (s, 3H), 2.55 (t, *J* = 7.6 Hz, 2H), 1.59 (quint, *J* = 8.3, 7.8 Hz, 2H), 1.30 (sext, *J* = 7.5 Hz, 2H), 0.85 (t, *J* = 7.3 Hz, 3H). ^13^C NMR (75 MHz, DMSO-*d*_6_) δ 165.0, 156.5, 146.6, 139.9, 137.7, 137.6, 136.1, 133.2, 133.1, 132.7, 131.2, 129.5, 129.3, 128.7, 128.2, 128.1, 127.9, 125.2, 123.8, 120.4, 110.7, 55.3, 46.3, 29.0, 25.9, 22.6, 21.7, 13.8. LCMS tr = 2.31 min, *m*/*z* calc for [M+H]^+^: 628, found: 628; [M-H]^−^: 626, found: 626. HRMS *m*/*z* calc for C_35_H_35_ClN_3_O_4_S [M+H]^+^ 628.2037, found 628.2067.

#### 4.1.18. Synthesis of 4′-((2-Butyl-4-chloro-5-(2-methoxybenzyl)-1H-imidazol-1-yl)methyl)-N-(cyclohexylcarbamoyl)-[1,1′-biphenyl]-2-sulfonamide (**14b**)

Cyclohexyl isocyanate (0.15 mL, 1.2 mmol) was added dropwise to a stirred mixture of sulfonyl amine **13** (520 mg, 1 mmol) and K_2_CO_3_ (0.41 g, 3 mmol) in acetone (50 mL). The reaction mixture was refluxed for 24 h. After cooling to room temperature, the mixture was transferred to an ice/water bath (100 mL) and acidified with 3 M hydrochloric acid (HCl). The resulting precipitate was isolated by filtration, washed with water and Et_2_O, and dried. The crude product was recrystallized from acetonitrile, yielding compound **14b**. White solid (77%); mp 176–178 °C (CH_3_CN). ^1^H NMR (500 MHz, DMSO-*d*_6_) δ 7.98 (dd, *J* = 7.9, 1.5 Hz, 1H), 7.53–7.38 (m, 2H), 7.32 (d, *J* = 6.4 Hz, 2H), 7.22–7.16 (m, 1H), 7.13–7.08 (m, 1H), 6.94 (d, *J* = 8.2 Hz, 1H), 6.89–6.78 (m, 4H), 5.56 (s, 1H), 5.08 (s, 2H), 3.79 (s, 2H), 3.77 (s, 3H), 3.21–3.13 (m, 1H), 2.56 (t, *J* = 7.4 Hz, 2H), 1.66–1.51 (m, 7H), 1.51–1.43 (m, 1H), 1.29 (sext, *J* = 7.3 Hz, 2H), 1.22–0.92 (m, 5H), 0.84 (t, *J* = 7.4 Hz, 3H). ^13^C NMR (126 MHz, DMSO-*d*_6_) δ 156.5, 146.6, 139.8, 139.3, 135.0, 131.8, 130.4, 129.5, 129.0, 128.5, 127.9, 126.9, 125.4, 125.3, 124.5, 123.7, 120.4, 110.7, 55.3, 47.6, 46.5, 33.0, 29.1, 26.0, 25.3, 24.5, 22.6, 21.8, 13.7. LCMS tr = 2.54 min, *m*/*z* calc for [M+H]^+^: 649, found: 649; [M-H]^−^: 647, found: 647. HRMS *m*/*z* calc for C_35_H_42_ClN_4_O_4_S [M+H]^+^ 649.2615, found 649.2601.

#### 4.1.19. Synthesis of Methyl 2-(4-((2-Butyl-4-chloro-5-(2-hydroxybenzyl)-1H-imidazol-1-yl)methyl) benzoyl)benzoate (**15**)

This compound was prepared by the same procedure as compound **5**, using compound **4c** (1.15 g, 2 mmol), Et_3_SiH (1.61 mL, 10 mmol) and TFA (4.46 mL, 60 mmol). Recrystallization from MeOH gave compound **15**. White solid (68%); mp 215–217 °C (MeOH). ^1^H NMR (300 MHz, DMSO-*d*_6_) δ 9.60 (br s, 1H), 7.98 (dd, *J* = 7.7, 1.4 Hz, 1H), 7.77 (td, *J* = 7.5, 1.5 Hz, 1H), 7.69 (td, *J* = 7.6, 1.5 Hz, 1H), 7.57–7.48 (m, 2H), 7.44 (dd, *J* = 7.3, 1.4 Hz, 1H), 7.03–6.89 (m, 3H), 6.81–6.60 (m, 3H), 5.28 (s, 2H), 3.71 (s, 2H), 3.56 (s, 3H), 2.49–2.46 (m, 2H), 1.47 (quint, *J* = 7.5 Hz, 2H), 1.26–1.17 (m, 2H), 0.78 (t, *J* = 7.3 Hz, 3H). ^13^C NMR (75 MHz, DMSO-*d*_6_) δ 195.5, 165.9, 154.4, 146.5, 142.3, 140.9, 135.7, 132.8, 130.2, 129.7, 129.2, 128.9, 128.7, 127.8, 127.5, 126.0, 125.1, 124.5, 123.7, 119.1, 115.0, 52.2, 46.4, 29.0, 25.9, 22.1, 21.6, 13.6. LCMS tr = 2.28 min, *m*/*z* calc for [M+H]^+^: 517, found: 517; [M-H]^−^: 515, found: 515. HRMS *m*/*z* calc for C_30_H_30_ClN_2_O_4_ [M+H]^+^ 517.1894, found 517.1903.

#### 4.1.20. Synthesis of 2-(4-((2-Butyl-4-chloro-5-(2-hydroxybenzyl)-1H-imidazol-1-yl)methyl)benzoyl) Benzoic Acid (**16**)

NaOH (200 mg, 5 mmol) in water (30 mL) was added to a stirred solution of compound **15** (0.52 g, 1 mmol) in CH_3_OH (10 mL). The resulting mixture was refluxed for 3 h and then concentrated in reduced pressure. The residue was diluted with water. The aqueous layer was acidified by addition of 1 M HCl and filtered. The precipitate was recrystallized in acetonitrile to give compound **16**. White solid (88%); mp 195–196 °C (CH_3_CN). ^1^H NMR (300 MHz, DMSO) δ 13.15 (br s, 1H), 9.59 (br s, 1H), 7.99 (dd, *J* = 7.6, 1.5 Hz, 1H), 7.72 (td, *J* = 7.4, 1.5 Hz, 1H), 7.64 (td, *J* = 7.5, 1.5 Hz, 1H), 7.52 (d, *J* = 8.3 Hz, 2H), 7.36 (dd, *J* = 7.4, 1.5 Hz, 1H), 7.02–6.92 (m, 3H), 6.83–6.52 (m, 3H), 5.16 (s, 2H), 3.71 (s, 2H), 2.45 (t, *J* = 7.8 Hz, 2H), 1.47 (quint, *J* = 6.9 Hz, 2H), 1.23 (sext, *J* = 7.3 Hz, 2H), 0.78 (t, *J* = 7.3 Hz, 3H). ^13^C NMR (75 MHz, DMSO) δ 195.9, 166.8, 154.4, 146.5, 142.1, 141.4, 135.9, 132.4, 129.9, 129.7, 129.3, 128.9, 127.5, 127.3, 125.9, 125.1, 124.5, 123.7, 119.1, 115.0, 46.4, 29.0, 25.9, 22.1, 21.6, 13.6. LCMS tr = 2.02 min, *m*/*z* calc for [M+H]^+^: 503, found: 503; [M-H]^−^: 501, Found: 501. HRMS *m*/*z* calc for C_29_H_28_ClN_2_O_4_ [M+H]^+^ 503.1738, Found 503.1708.

### 4.2. Biological Methods

#### 4.2.1. Membrane Preparation

PLC-PRF Membranes (Human Hepatoma Cell Line): Crude membranes were prepared following the protocol described by Maeda et al. [21]. Cells were lysed in Tris/HCl buffer (50 mM, MgCl_2_ 2 mM, pH 7.4) using a Polytron (1100 rpm, maximum setting, 10 s). The homogenate was centrifuged at 30,000× *g* for 15 min at 4 °C, and the pellet was resuspended in Tris/HCl buffer (20 mM, sucrose 250 mM, pH 7.4) at a membrane concentration of 2 mM, and then stored at −70 °C until use.

Calf Cerebellum Membranes: Prepared using the same protocol after dissection on dental wax over ice. Tissue was homogenized in 40 volumes of ice-cold Tris/HCl buffer using a Polytron (setting 5, 20 s) before the centrifugation step.

#### 4.2.2. AT_1_ Receptor Radiobinding Assay (PLC-PRF Cells)

Aliquots containing 100 µg of protein were incubated at 25 °C for 1 h in the same Tris/HCl buffer used for membrane preparation. Incubation was initiated by adding 3 nM [^3^H]AII in a total volume of 500 µL. Non-specific binding was measured in the presence of 1 µM losartan. Test compounds were studied in a concentration range of 10^−10^ to 10^−5^ M. Binding was terminated by rapid vacuum filtration onto glass fiber filters (GF/C Whatman pre-incubated in 0.1% polyethyleneimine for 2 h). Filters were washed four times with 1 mL of ice-cold buffer. Dry filters were placed into vials containing 2 mL of scintillation fluid, and radioactivity was measured using a scintillation counter (model 1609, Wallac, Turku, Finland). Ki values (concentration for 50% displacement of specifically bound [^3^H]AII) were calculated using the Cheng and Prussof equation integrated into graphic software (Kaleidagraph for Macintosh, version 5.0.6).

#### 4.2.3. AT_2_ Receptor Radiobinding Assay (Calf Cerebellum Cortex)

Membranes were pre-incubated without [^3^H]AII in sodium phosphate buffer (100 mM, pH 7.0) at 37 °C for 30 min in the presence of DTT (1 mM), PMSF (0.1 mM), EDTA (5 mM), and BSA (0.5%) to eliminate residual AT_1_ receptor binding. Membranes were pelleted and resuspended in the same assay mixture. Membranes (300 µg) were incubated with [^3^H]AII (5 nM) at 25 °C for 60 min. Filtration, washing, and calculation procedures were performed as described above. Non-specific binding was measured in the presence of PD 123177 (1 µM), a selective AT_2_ receptor ligand.

### 4.3. Bioinformatics

The AT_1_ structure bound to losartan was sourced from the Protein Data Bank (PDB ID: 8TH4). Losartan was removed, and both polar and nonpolar hydrogen atoms and assigned Gasteiger charges were added to the structure with ChimeraX [22]. The docking was performed with GNINA [23] from the prebuild binary. Docking parameters were set with autobox function around the losartan recovered (PDB ID: 8TH4), with 3 Å added for each side and exhaustiveness at 64. Initial docking yielded cnn_pose_score and cnn_affinity scores; GNINA’s CNN algorithm then prioritized conformations based on cnn_pose_score (ranging from 0 to 1, indicating binding potential), while cnn_affinity provided additional binding potential assessment, with higher values indicating stronger binding. Binding poses matching the imidazole position from losartan were selected to perform comparison of the interactions.

## Figures and Tables

**Table 1:** AT_1_ receptor binding affinities (Ki) and functional IC_50_ values of imidazole derivatives.

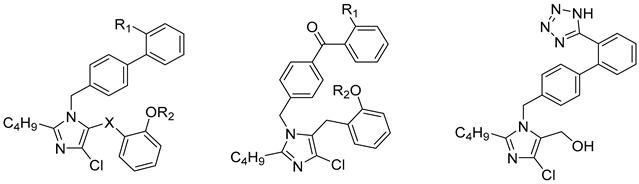					
4b, 5–14		15–16		Losartan	
N°	R_1_	R_2_	X	Ki (nM) ^a^ AT_1_	IC_50_ (nM) ^b^ AT_1_
losartan	**-**	**-**	**-**	3	20 ^b^
4b	SO_2_NHt-Bu	CH_3_	CHOH	158	794
5	tetrazole	CH_3_	CH_2_	25	32
6	tetrazole	H	CH_2_	16	20
7	SO_2_NH_2_	H	CH_2_	1000	1585
8	SO_2_NHCOC_6_H_5_	COC_6_H_5_	CH_2_	25	40
9	SO_2_NHCOC_6_H_5_	H	CH_2_	25	50
10	SO_2_NH_2_	CH_3_	CO	25	50
11	SO_2_NHCOC_6_H_5_	CH_3_	CO	5	10
12	SO_2_NHCOC_6_H_5_	H	CO	10	16
13	SO_2_NH_2_	CH_3_	CH_2_	501	1000
14a	SO_2_NHCOC_6_H_5_	CH_3_	CH_2_	16	32
14b	SO_2_NHCONHC_6_H_11_	CH_3_	CH_2_	20	40
15	CO_2_CH_3_	H	CH_2_	nd ^c^	-
16	CO_2_H	H	CH_2_	251	501

^a^ AT_1_ binding, human hepatoma cell line, PLC-PRF-5. Each value represents the mean values. ^b^ IC_50_ value from Burnier et al. [9] ^c^ Inhibition of 35% at 10^−5^ M. For the AT_2_ affinity, the percentage of inhibition is <20% at 10^−5^ M for all the compounds.

## Data Availability

The original contributions presented in this study are included in the article/Supplementary Material. Further inquiries can be directed to the corresponding authors.

## Supplementary Materials

The following supporting information can be downloaded at: https://www.mdpi.com/article/10.3390/molecules31111971/s1, Table S1: Output of the Deep-PK API (https://biosig.lab.uq.edu.au/deeppk/) for in this order: compound **14a**, **11** & **12** compared to losartan as last molecule; LCMS, ^1^H & ^13^C NMR data for all compounds and HRMS data for all final compounds.
