# Supplementary material for: Imidazole-Based AT1 Receptor Ligands: Design, Synthesis and Pharmacological Evaluation
Source: Molecules. 2026 Jun 5;31(11):1971. doi: 10.3390/molecules31111971 (PMC13258302; doi:10.3390/molecules31111971)
Supplement: Supplementary file 1 [file molecules-31-01971-s001.zip › molecules-4322715-supplementary.pdf]

Supplementary Materials

Imidazole-based AT1 Receptor Ligands: Design, Synthesis and Pharmacological Evaluation

Florian Descamps[a], Marouane Rami[a], Jean François Goossens[b], Patricia Melnyk[a], Maxime Liberelle[a], Saïd Yous[b*]

[a]UMR-S 1172-LilNCog-Lille Neuroscience & Cognition, Univ. Lille, Inserm, CHU Lille, 59000, Lille, France; [b]Univ. Lille, CHU Lille, ULR 7365 GRITA - Groupe de Recherche sur les Formes Injectables et Technologies Associées, F-59000, Lille, France.

* Address for correspondence: Saïd Yous, said.yous@univ-lille.fr, U1172 - LilNCog - Lille Neuroscience & Cognition, UFR3S-Pharmacie, 3 rue du Professeur Laguesse, F-59000 Lille, France.

Table S1. Output of the Deep-PK API (https://biosig.lab.uq.edu.au/deeppk/) for in this order : compound **14a**, **11** & **12** compared to losartan as last molecule.

Absorption<br>Caco-2 (logPaap)	Absorption<br>Human Oral Bioavailability 20%	Absorption<br>Human Intestinal Absorption	Absorption<br>Madin-Darby Canine Kidney	Absorption<br>Human Oral Bioavailability 50%	Absorption<br>P-Glycoprotein Inhibitor	Absorption<br>P-Glycoprotein Substrate	Absorption<br>Skin Permeability
-5.32	Bioavailable (Low Confidence)	Absorbed (High Confidence)	-4.62	Bioavailable (Medium Confidence)	Inhibitor (High Confidence)	Non-Substrate (High Confidence)	37.91
-5.44	Bioavailable (Low Confidence)	Absorbed (High Confidence)	-4.69	Bioavailable (Medium Confidence)	Inhibitor (High Confidence)	Non-Substrate (Medium Confidence)	23.70
-5.45	Bioavailable (Low Confidence)	Absorbed (High Confidence)	-4.77	Bioavailable (Low Confidence)	Inhibitor (High Confidence)	Non-Substrate (High Confidence)	22.46
-5.38	Bioavailable (Low Confidence)	Absorbed (High Confidence)	-5.01	Non-Bioavailable (Low Confidence)	Non-Inhibitor (High Confidence)	Non-Substrate (Low Confidence)	-1.38

Distribution<br>Blood-Brain Barrier Central Nervous System	Distribution<br>Blood-Brain Barrier	Distribution<br>Fraction Unbound (Human)	Distribution<br>Plasma Protein Binding	Distribution<br>Steady State Volume of Distribution
-2.51	Non-Penetrable (High Confidence)	2.31	104.97	0.85
-2.34	Non-Penetrable (High Confidence)	2.51	104.65	0.93
-2.94	Non-Penetrable (High Confidence)	2.15	107.04	0.86
-2.46	Non-Penetrable (High Confidence)	1.59	97.10	1.05

Metabolism<br>Breast Cancer Resistance Protein	Metabolism<br>CYP 1A2 Inhibitor	Metabolism<br>CYP 1A2 Substrate	Metabolism<br>CYP 2C19 Inhibitor	Metabolism<br>CYP 2C19 Substrate	Metabolism<br>CYP 2C9 Inhibitor	Metabolism<br>CYP 2C9 Substrate	Metabolism<br>CYP 2D6 Inhibitor	Metabolism<br>CYP 2D6 Substrate	Metabolism<br>CYP 3A4 Inhibitor	Metabolism<br>CYP 3A4 Substrate	Metabolism<br>OATP1B1
Non-Inhibitor (Medium Confidence)	Non-Inhibitor (Medium Confidence)	Non-Substrate (High Confidence)	Inhibitor (High Confidence)	Non-Substrate (High Confidence)	Inhibitor (High Confidence)	Non-Substrate (Low Confidence)	Non-Inhibitor (High Confidence)	Non-Substrate (Low Confidence)	Inhibitor (High Confidence)	Substrate (Medium Confidence)	Inhibitor (High Confidence)
Non-Inhibitor (Medium Confidence)	Non-Inhibitor (High Confidence)	Non-Substrate (High Confidence)	Inhibitor (High Confidence)	Non-Substrate (Medium Confidence)	Inhibitor (High Confidence)	Substrate (Medium Confidence)	Non-Inhibitor (Low Confidence)	Non-Substrate (Low Confidence)	Inhibitor (High Confidence)	Substrate (High Confidence)	Inhibitor (High Confidence)
Non-Inhibitor (High Confidence)	Inhibitor (Low Confidence)	Non-Substrate (High Confidence)	Inhibitor (High Confidence)	Non-Substrate (High Confidence)	Inhibitor (High Confidence)	Substrate (High Confidence)	Inhibitor (Low Confidence)	Non-Substrate (Low Confidence)	Inhibitor (High Confidence)	Non-Substrate (Low Confidence)	Inhibitor (High Confidence)
Non-Inhibitor (High Confidence)	Non-Inhibitor (Medium Confidence)	Non-Substrate (High Confidence)	Inhibitor (High Confidence)	Non-Substrate (Medium Confidence)	Inhibitor (High Confidence)	Substrate (High Confidence)	Inhibitor (Medium Confidence)	Non-Substrate (Medium Confidence)	Inhibitor (High Confidence)	Substrate (High Confidence)	Inhibitor (High Confidence)

Excretion<br>Clearance	Excretion<br>Organic Cation Transporter 2	Excretion<br>Half-Life of Drug
3.58	Non-Inhibitor (Medium Confidence)	Half-Life < 3hs (High Confidence)
4.43	Non-Inhibitor (Medium Confidence)	Half-Life < 3hs (High Confidence)
5.05	Non-Inhibitor (Medium Confidence)	Half-Life < 3hs (Medium Confidence)
6.75	Non-Inhibitor (Medium Confidence)	Half-Life < 3hs (High Confidence)

Toxicity AMES Mutagenesis	Toxicity Avian	Toxicity Bee	Toxicity Bioconcentration Factor	Toxicity Biodegradation	Toxicity Carcinogenesis	Toxicity Crustacean	Toxicity Liver Injury I (DILI)	Toxicity Eye Corrosion	Toxicity Eye irritation
Safe (High Confidence)	Safe (High Confidence)	Toxic (Low Confidence)	0.80	Safe (High Confidence)	Safe (High Confidence)	Toxic (High Confidence)	Safe (Low Confidence)	Safe (High Confidence)	Safe (High Confidence)
Safe (High Confidence)	Safe (High Confidence)	Toxic (Low Confidence)	1.43	Safe (High Confidence)	Safe (High Confidence)	Toxic (High Confidence)	Safe (Medium Confidence)	Safe (High Confidence)	Safe (High Confidence)
Safe (High Confidence)	Safe (High Confidence)	Toxic (Low Confidence)	0.61	Safe (High Confidence)	Safe (High Confidence)	Toxic (High Confidence)	Safe (Low Confidence)	Safe (High Confidence)	Safe (High Confidence)
Safe (High Confidence)	Safe (High Confidence)	Toxic (Low Confidence)	0.52	Safe (High Confidence)	Safe (High Confidence)	Toxic (High Confidence)	Safe (High Confidence)	Safe (High Confidence)	Safe (High Confidence)

Toxicity Maximum Tolerated Dose	Toxicity Liver Injury II	Toxicity hERG Blockers	Toxicity Daphnia Maga	Toxicity Micronucleos	Toxicity NR-AhR	Toxicity NR-AR	Toxicity NR-AR-LBD	Toxicity NR-Aromatase	Toxicity NR-ER	Toxicity NR-ER-LBD
-0.20	Toxic (High Confidence)	Safe (Medium Confidence)	9.32	Toxic (High Confidence)	Safe (High Confidence)	Safe (Medium Confidence)	Safe (High Confidence)	Safe (High Confidence)	Safe (High Confidence)	Safe (High Confidence)
-0.26	Toxic (Medium Confidence)	Safe (Low Confidence)	8.03	Toxic (High Confidence)	Safe (High Confidence)	Safe (Medium Confidence)	Safe (High Confidence)	Safe (High Confidence)	Safe (High Confidence)	Safe (High Confidence)
-0.03	Toxic (High Confidence)	Safe (High Confidence)	8.08	Toxic (High Confidence)	Safe (High Confidence)	Safe (Medium Confidence)	Safe (High Confidence)	Safe (High Confidence)	Safe (High Confidence)	Safe (High Confidence)
0.28	Toxic (Medium Confidence)	Toxic (Medium Confidence)	7.24	Toxic (High Confidence)	Safe (High Confidence)	Safe (High Confidence)	Safe (High Confidence)	Safe (High Confidence)	Safe (High Confidence)	Safe (High Confidence)

Toxicity NR-GR	Toxicity NR-PPAR-gamma	Toxicity NR-TR	Toxicity T. Pyriformis	Toxicity Rat (Acute)	Toxicity Rat (Chronic Oral)	Toxicity Fathead Minnow	Toxicity Respiratory Disease
Safe (Medium Confidence)	Safe (High Confidence)	Safe (High Confidence)	-72476.12	2.73	1.61	105.61	Safe (High Confidence)
Safe (High Confidence)	Safe (High Confidence)	Safe (High Confidence)	-47287.40	2.75	1.45	75.04	Safe (Low Confidence)
Safe (Medium Confidence)	Safe (High Confidence)	Safe (High Confidence)	-42252.59	2.61	1.73	70.99	Safe (High Confidence)
Safe (High Confidence)	Safe (High Confidence)	Safe (High Confidence)	-66.37	2.64	1.75	4.78	Toxic (High Confidence)

Toxicity Skin Sensitisation	Toxicity SR-ARE	Toxicity SR-ATAD5	Toxicity SR-HSE	Toxicity SR-MMP	Toxicity SR-p53
Safe (Medium Confidence)	Toxic (High Confidence)	Safe (High Confidence)	Safe (High Confidence)	Toxic (Medium Confidence)	Safe (High Confidence)
Safe (Low Confidence)	Toxic (Medium Confidence)	Safe (High Confidence)	Safe (High Confidence)	Toxic (Low Confidence)	Safe (High Confidence)
Safe (Medium Confidence)	Toxic (High Confidence)	Safe (High Confidence)	Safe (High Confidence)	Toxic (Medium Confidence)	Safe (High Confidence)
Toxic (Low Confidence)	Toxic (Low Confidence)	Safe (High Confidence)	Safe (High Confidence)	Toxic (Low Confidence)	Safe (High Confidence)

Contents

LCMS, ^{1}H & ^{13}C NMR data for all compounds and HRMS data for all final compounds.

2-Butyl-4-chloro-1-((2'-(1-trityl-1H-tetrazol-5-yl)-[1,1'-biphenyl]-4-yl)methyl)-1H-imidazole-5-carbaldehyde (3a). 5
N-tert-butyl-2-[4-[(2-butyl-4-chloro-5-formyl-imidazol-1-yl)methyl]phenyl]benzenesulfonamide (3b). 8
N-tert-butyl-2-[4-[[2-butyl-4-chloro-5-[hydroxy-(2-methoxyphenyl)methyl]imidazol-1-yl]methyl]phenyl] benzenesulfonamide (4b). 11
Methyl 2-(4-((2-butyl-4-chloro-5-(hydroxy(2-(methoxymethoxy)phenyl)methyl)-1H-imidazol-1-yl)methyl) benzoyl)benzoate (4c). 15
2-((1-((2'-(1H-Tetrazol-5-yl)-[1,1'-biphenyl]-4-yl)methyl)-2-butyl-4-chloro-1H-imidazol-5-yl)methyl)phenol (6). 19
4'-((2-Butyl-4-chloro-5-(2-hydroxybenzyl)-1H-imidazol-1-yl)methyl)-[1,1'-biphenyl]-2-sulfonamide (7). 23
2-((1-((2'-(N-Benzoylsulfamoyl)-[1,1'-biphenyl]-4-yl)methyl)-2-butyl-4-chloro-1H-imidazol-5-yl)methyl) phenyl benzoate (8). 27
N-((4'-((2-butyl-4-chloro-5-(2-hydroxybenzyl)-1H-imidazol-1-yl)methyl)-[1,1'-biphenyl]-2-yl)sulfonyl)benzamide (9). 31
N-(tert-Butyl)-4'-((2-butyl-4-chloro-5-(2-methoxybenzoyl)-1H-imidazol-1-yl)methyl)-[1,1'-biphenyl]-2-sulfonamide (10). 36
N-((4'-((2-Butyl-4-chloro-5-(2-methoxybenzoyl)-1H-imidazol-1-yl)methyl)-[1,1'-biphenyl]-2-yl)sulfonyl) benzamide (11). 40
N-((4'-((2-Butyl-4-chloro-5-(2-hydroxybenzoyl)-1H-imidazol-1-yl)methyl)-[1,1'-biphenyl]-2-yl)sulfonyl) benzamide (12). 44
4'-((2-Butyl-4-chloro-5-(2-methoxybenzyl)-1H-imidazol-1-yl)methyl)-[1,1'-biphenyl]-2-sulfonamide (13). 48
N-((4'-((2-Butyl-4-chloro-5-(2-methoxybenzyl)-1H-imidazol-1-yl)methyl)-[1,1'-biphenyl]-2-yl)sulfonyl) benzamide (14a). 52
4'-((2-Butyl-4-chloro-5-(2-methoxybenzyl)-1H-imidazol-1-yl)methyl)-N-(cyclohexylcarbamoyl)-[1,1'-biphenyl]-2-sulfonamide (14b). 56
Methyl 2-(4-((2-butyl-4-chloro-5-(2-hydroxybenzyl)-1H-imidazol-1-yl)methyl)benzoyl)benzoate (15). 60
2-(4-((2-Butyl-4-chloro-5-(2-hydroxybenzyl)-1H-imidazol-1-yl)methyl)benzoyl)benzoic acid (16). 64

2-Butyl-4-chloro-1-((2'-(1-trityl-1H-tetrazol-5-yl)-[1,1'-biphenyl]-4-yl)methyl)-1H-imidazole-5-carbaldehyde (3a).

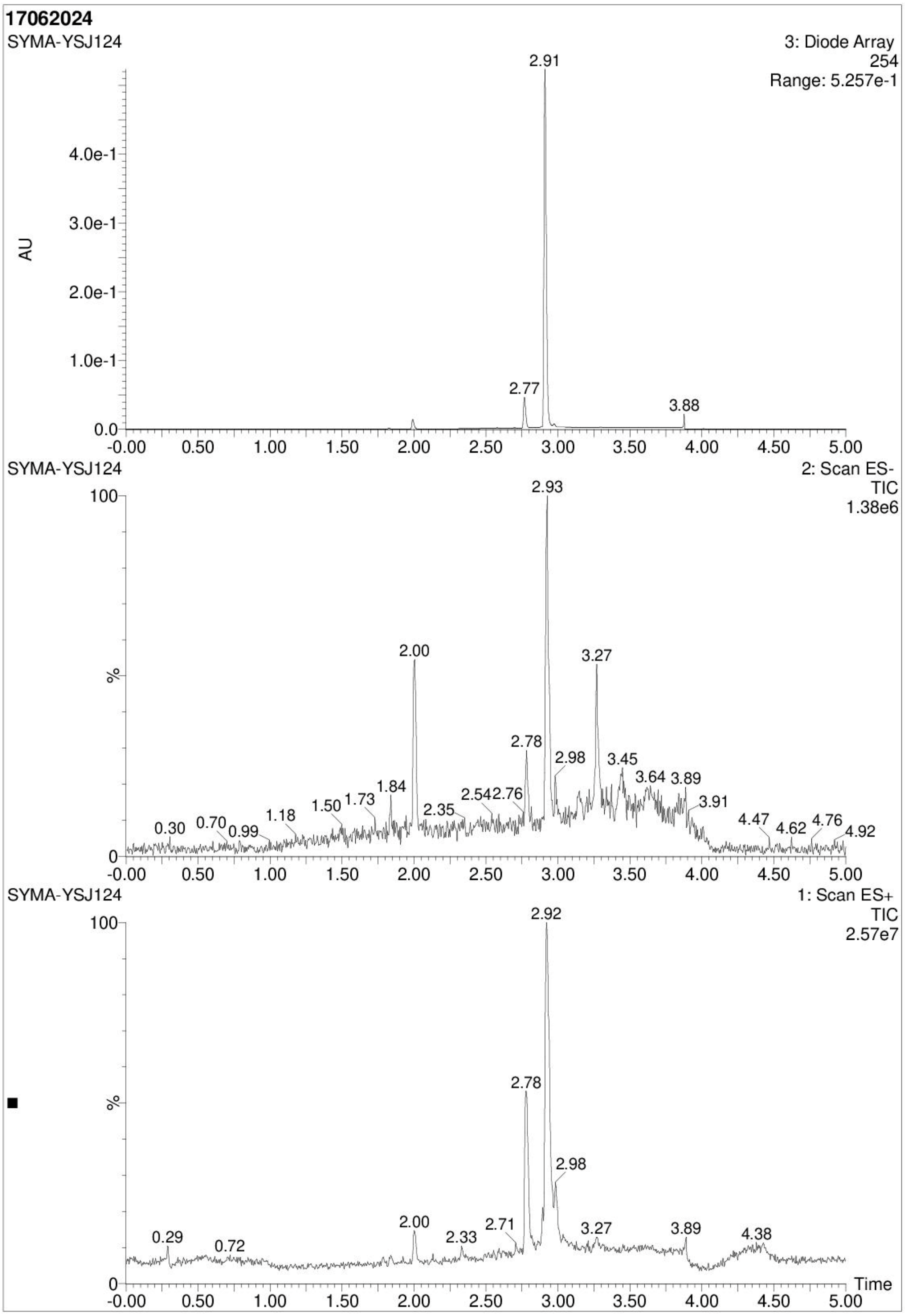

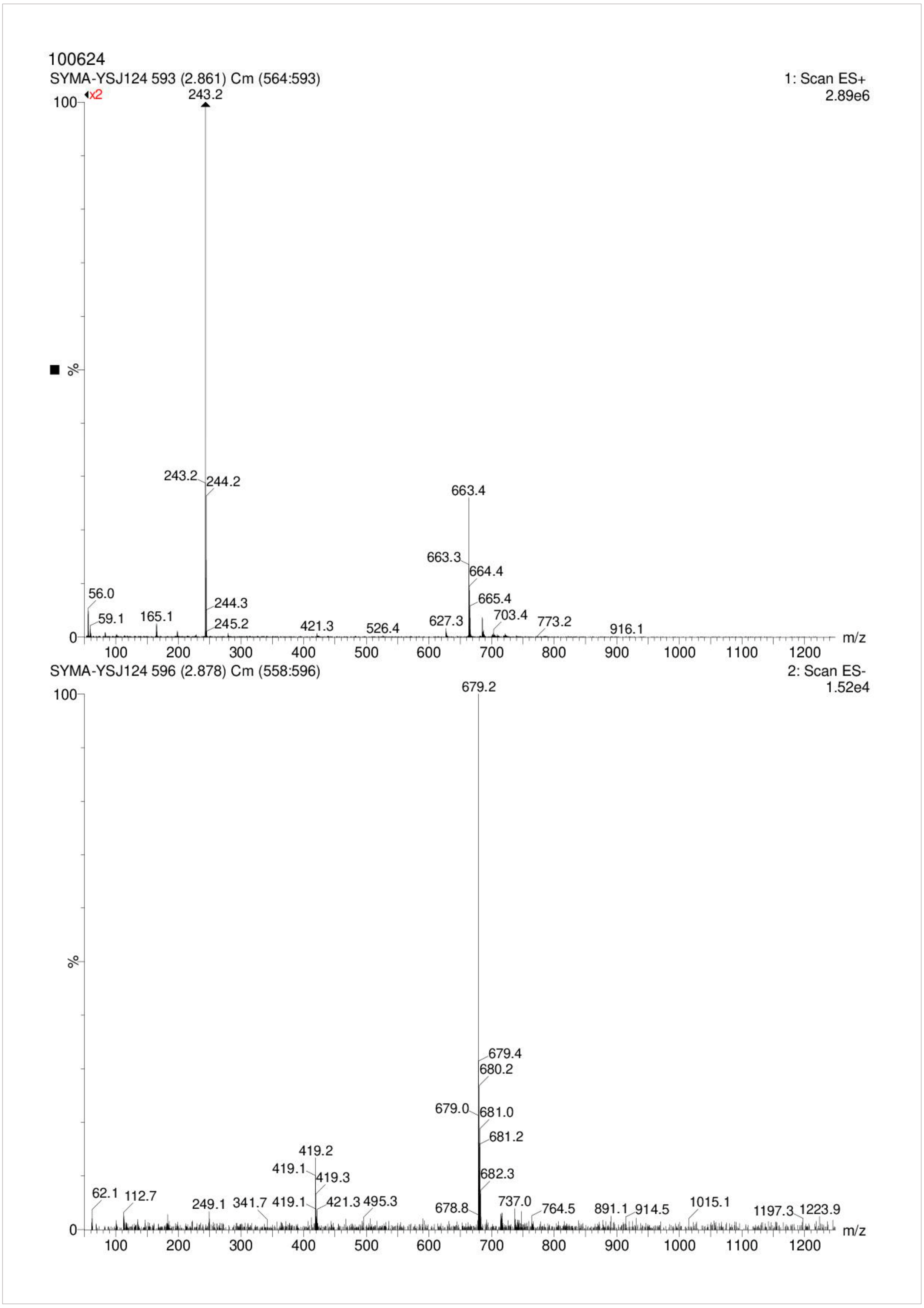
100624
SYMA-YSJ124 593 (2.861) Cm (564:593)
1: Scan ES+
2.89e6
x2
243.2
244.2
663.4
663.3
664.4
56.0
59.1
165.1
244.3
245.2
421.3
526.4
627.3
665.4
703.4
773.2
916.1
%
m/z
SYMA-YSJ124 596 (2.878) Cm (558:596)
2: Scan ES-
1.52e4
679.2
679.4
680.2
679.0
681.0
681.2
682.3
419.2
419.1
419.3
62.1
112.7
249.1
341.7
421.3
495.3
678.8
737.0
764.5
891.1
914.5
1015.1
1197.3
1223.9

YSI124-1H

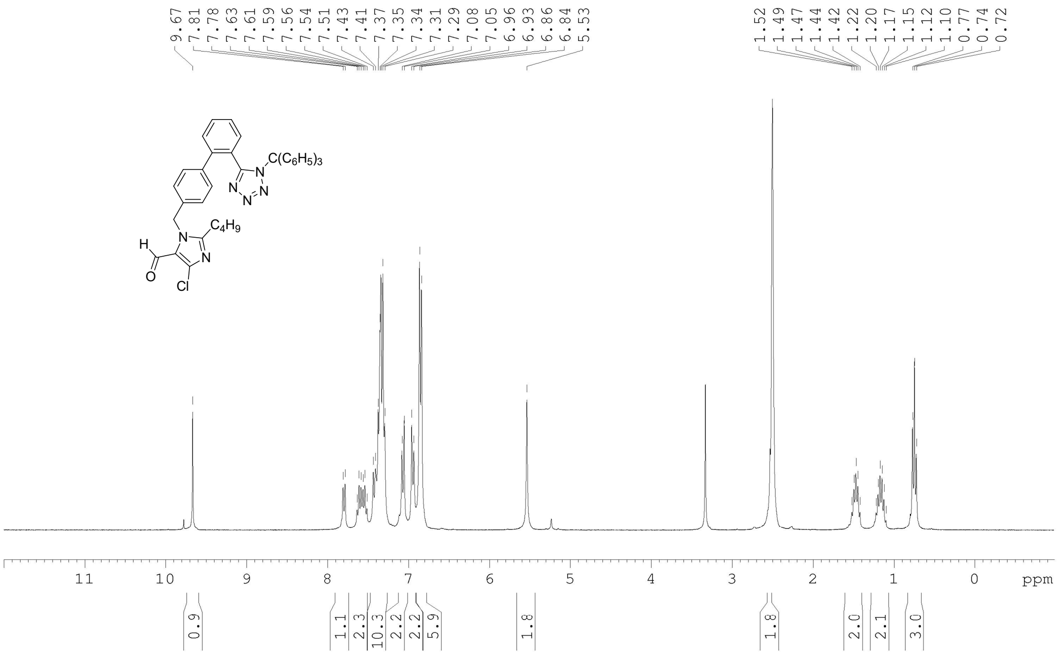

YSI124-13C

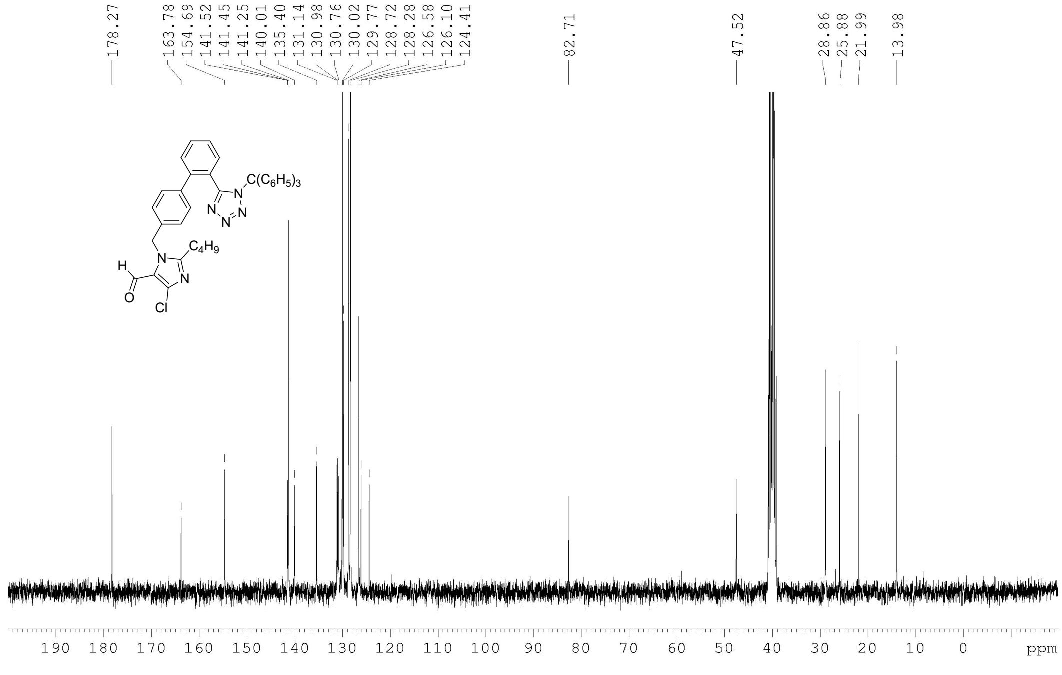

N-tert-butyl-2-[4-[(2-butyl-4-chloro-5-formyl-imidazol-1-yl)methyl]phenyl]benzenesulfonamide (3b).

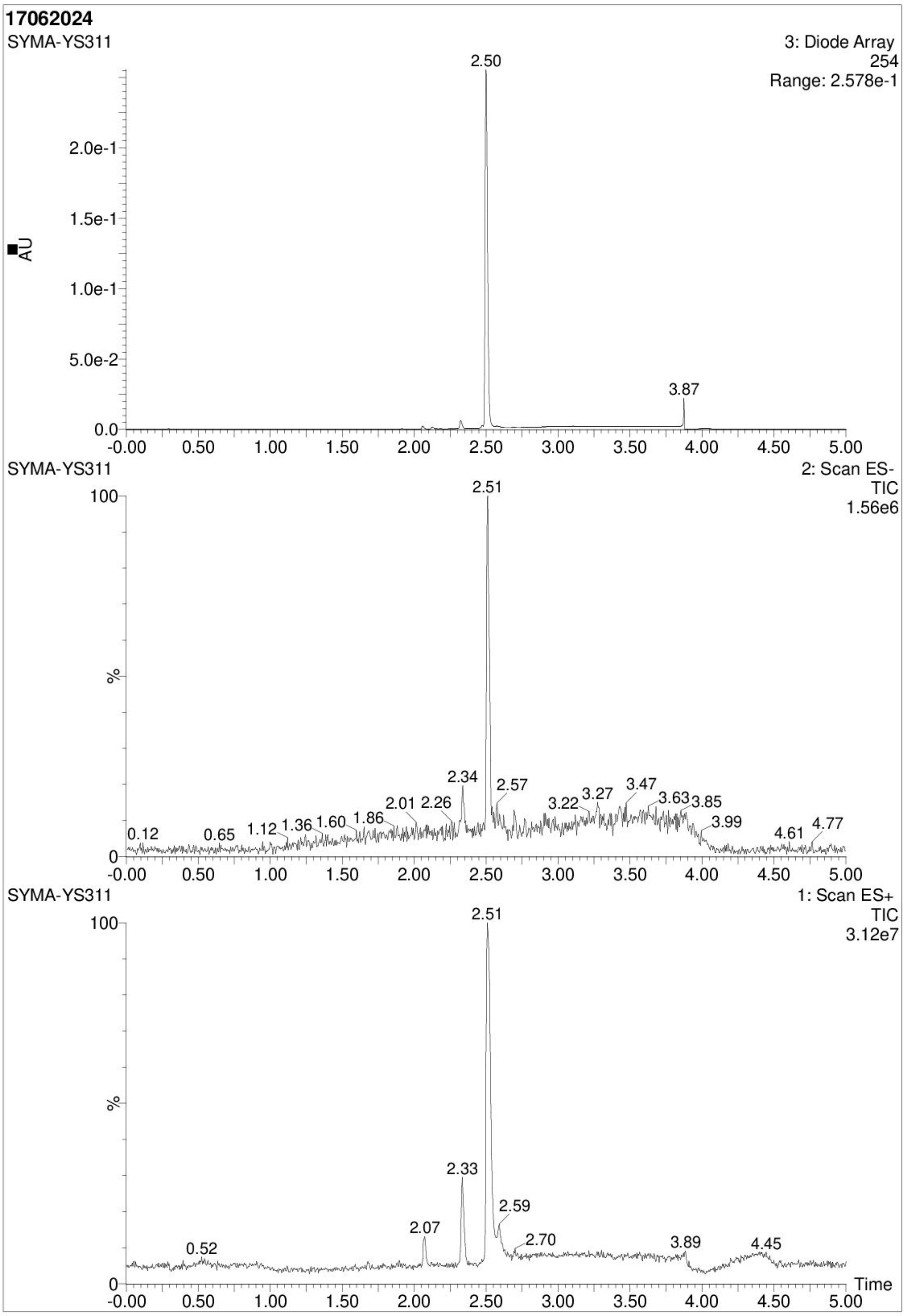

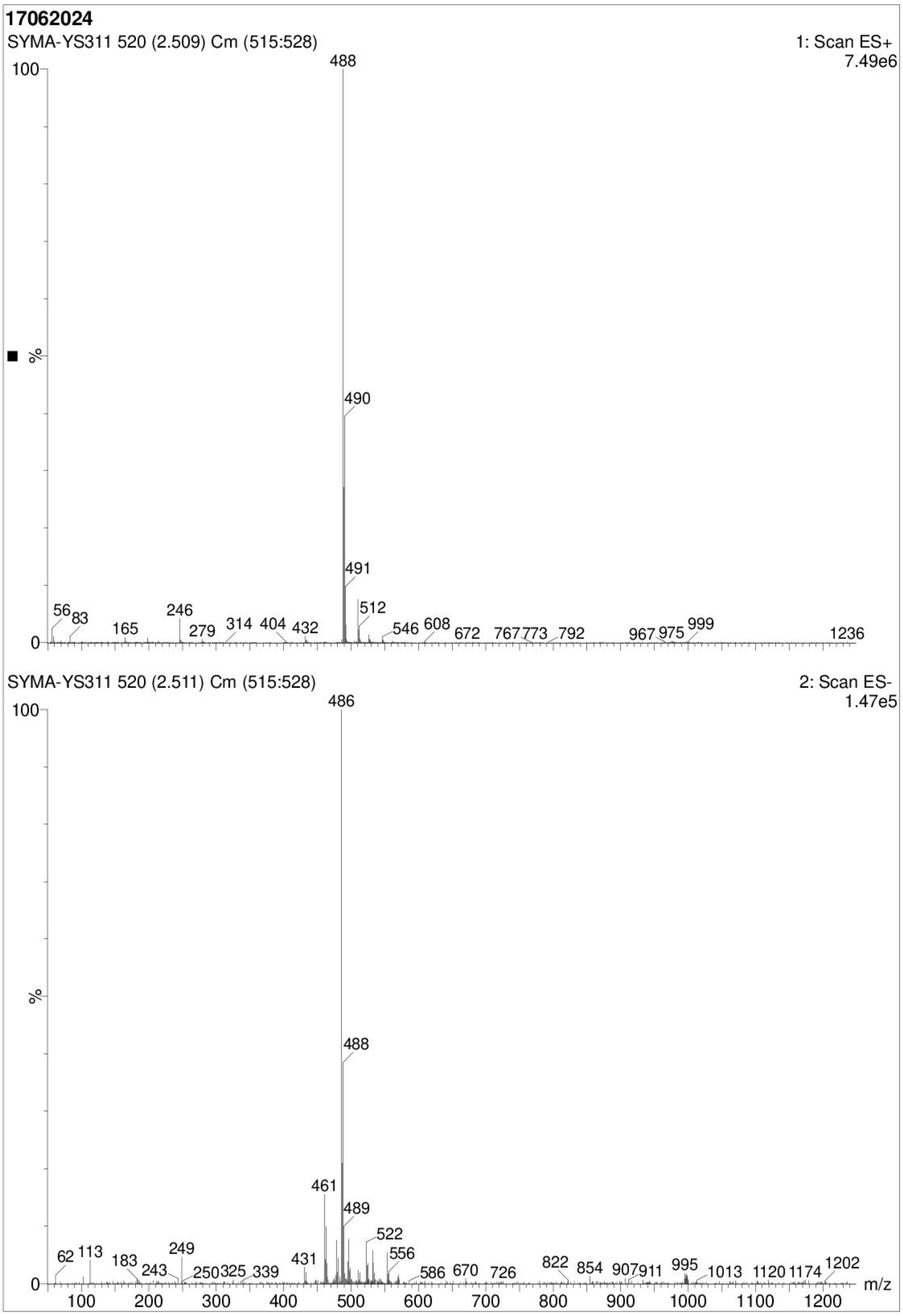
17062024
SYMA-YS311 520 (2.509) Cm (515:528)
1: Scan ES+
7.49e6
100
%
0
488
490
491
512
56
83
165
246
279
314
404
432
546
608
672
767
773
792
967
975
999
1236
SYMA-YS311 520 (2.511) Cm (515:528)
2: Scan ES-
1.47e5
486
488
461
489
522
556
62
113
183
243
249
250
325
339
431
586
670
726
822
854
907
911
995
1013
1120
1174
1202
m/z
100
200
300
400
500
600
700
800
900
1000
1100
1200

YS311-1H

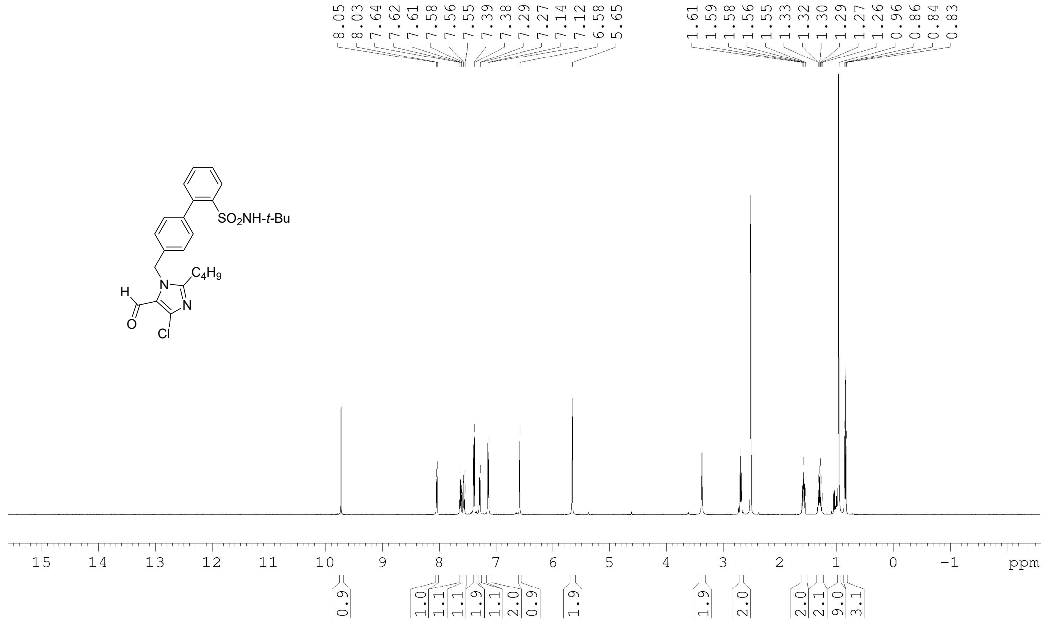

YS311-13C

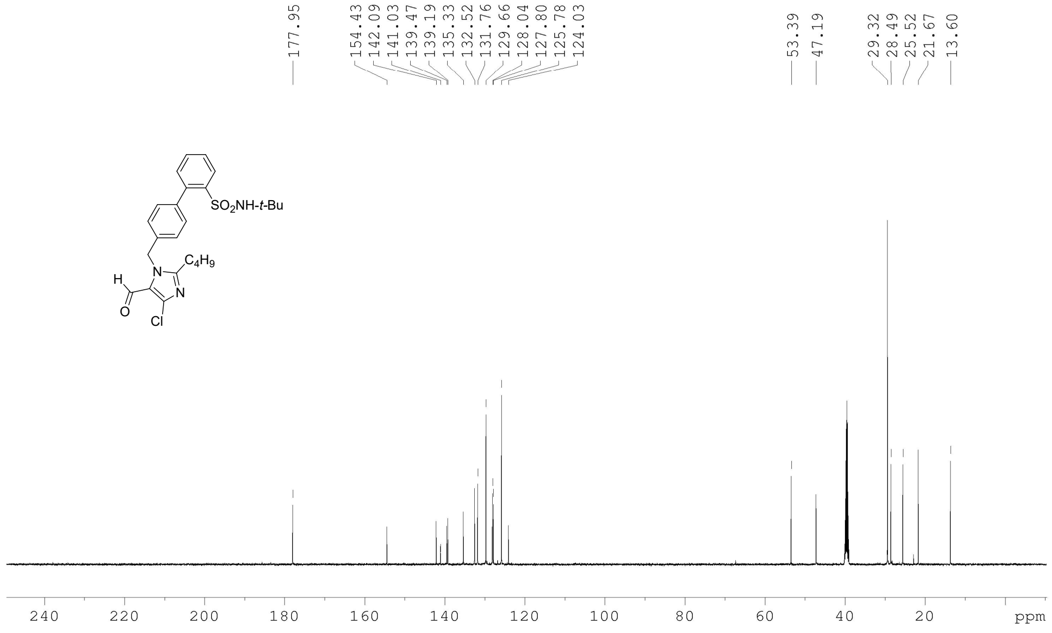

N-tert-butyl-2-[4-[[2-butyl-4-chloro-5-[hydroxy-(2-methoxyphenyl)methyl]imidazol-1-yl]methyl]phenyl] benzenesulfonamide (4b).

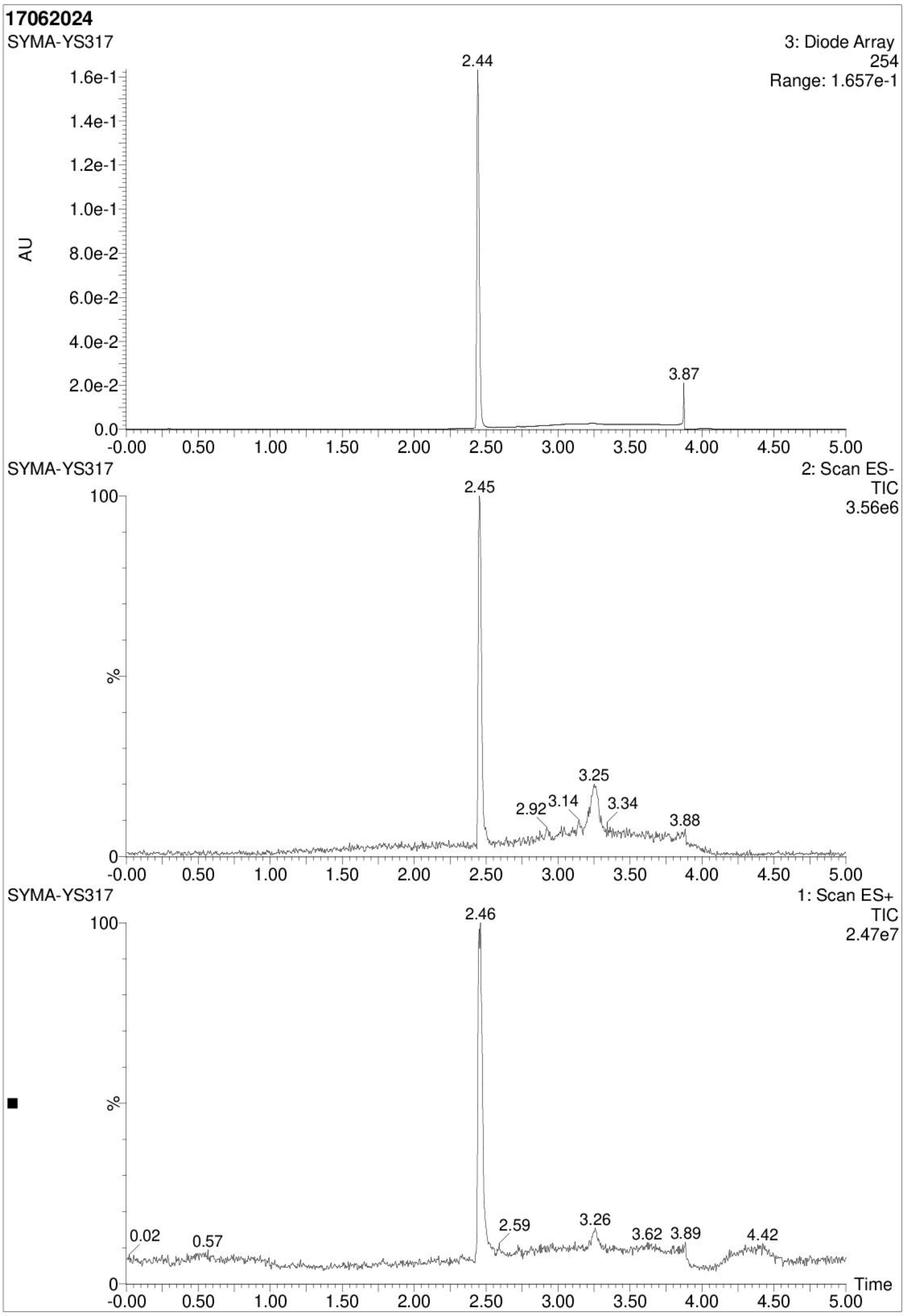

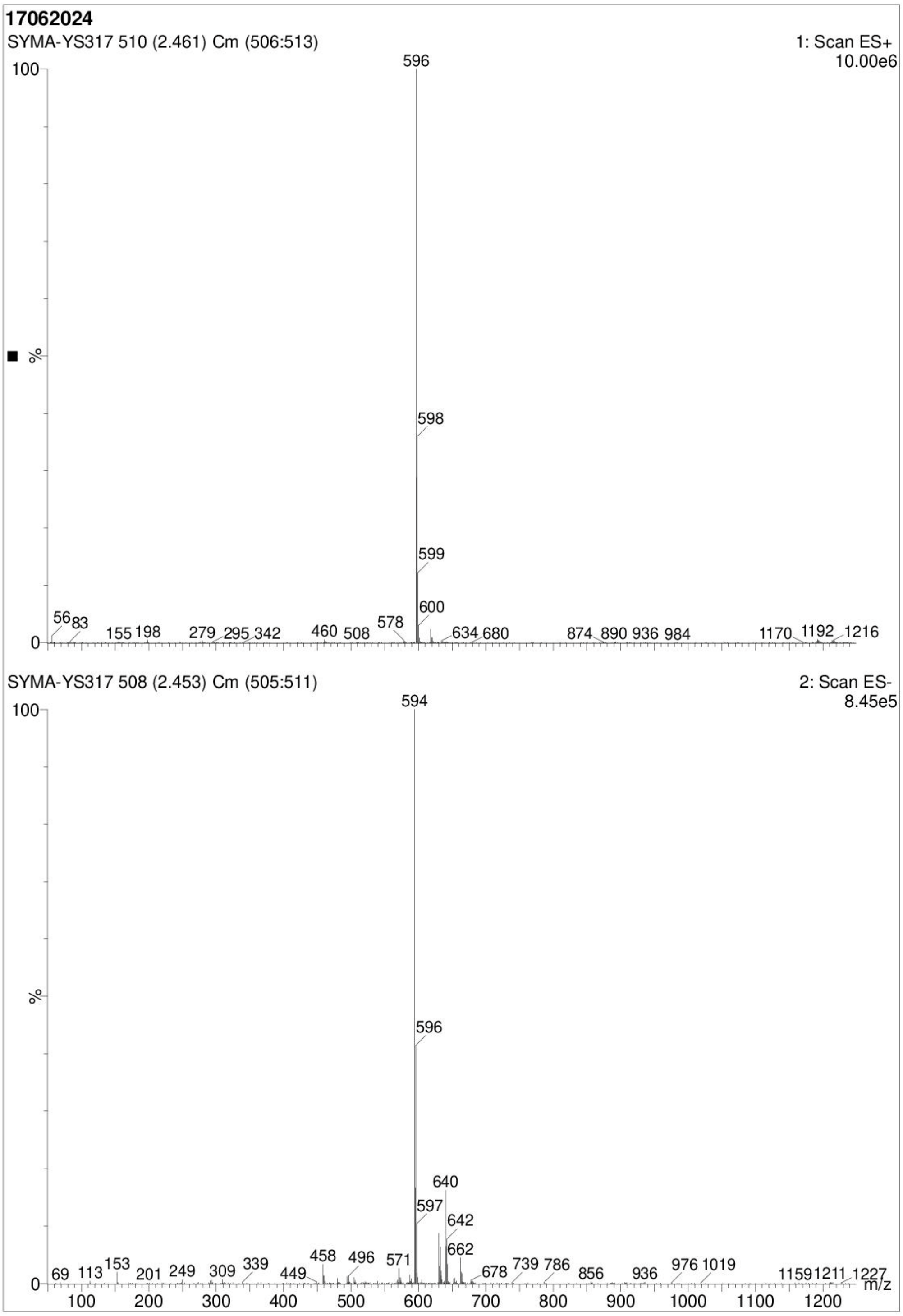
17062024
SYMA-YS317 510 (2.461) Cm (506:513)
1: Scan ES+
10.00e6
596
598
599
600
%
SYMA-YS317 508 (2.453) Cm (505:511)
2: Scan ES-
8.45e5
594
596
640
597
642
662
m/z

YS317-1H

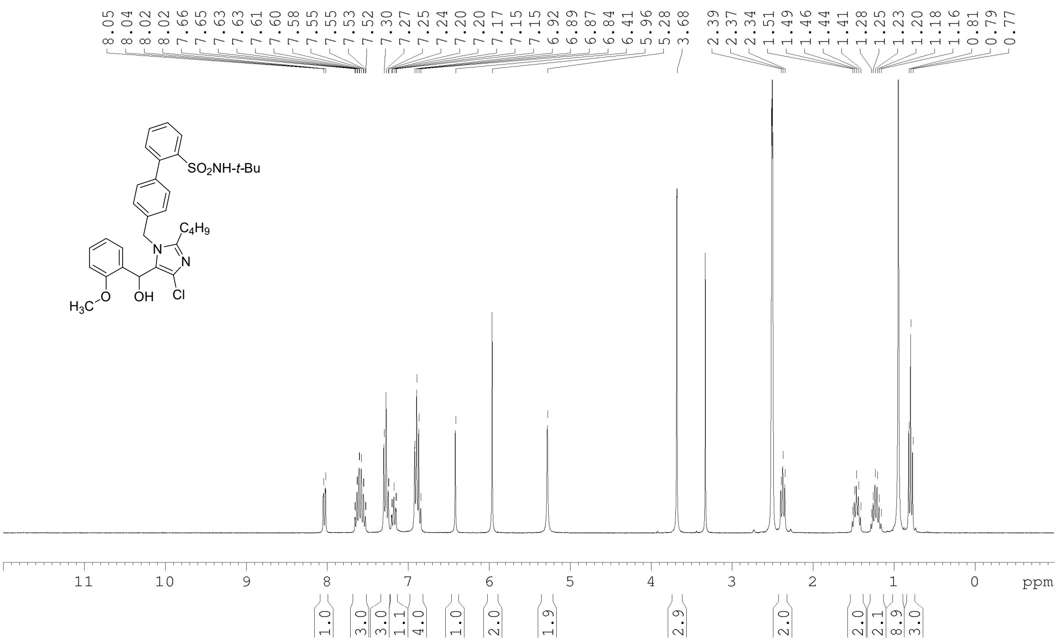

YS317-13C

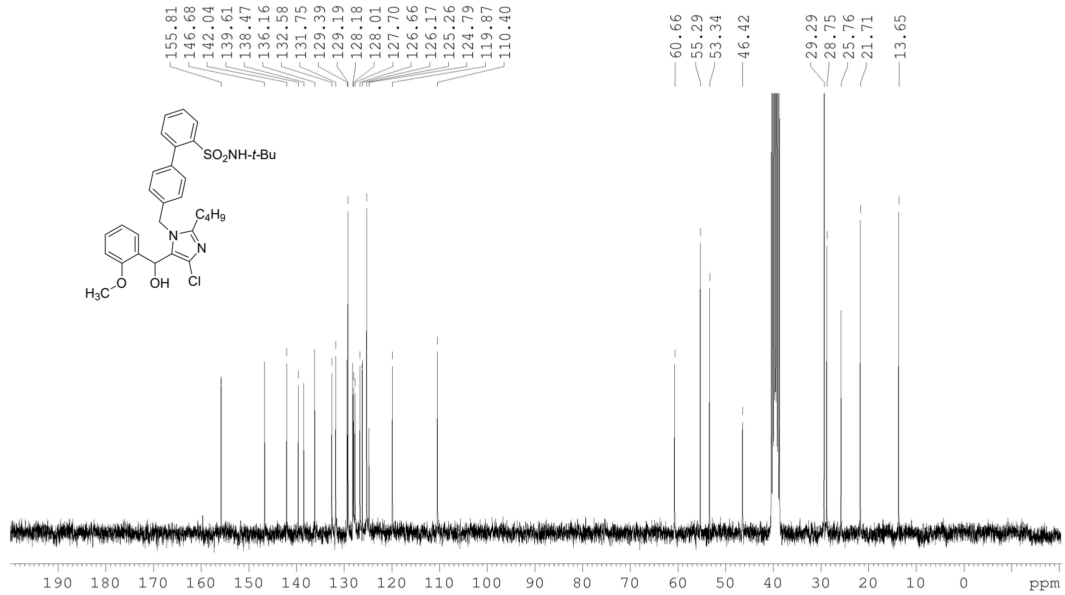

Single Mass Analysis

Tolerance = 5.0 mDa / DBE: min = -1.5, max = 50.0
Selected filters: None

Monoisotopic Mass, Even Electron Ions
252 formula(e) evaluated with 2 results within limits (up to 1000 best isotopic matches for each mass)
Elements Used:
C: 0-33 H: 0-39 N: 0-3 O: 0-7 F: 0-1 S: 0-1 Cl: 0-1

SYMA
YS317-HRMS 117 (2.730) 1: TOF MS ES+ 3.11e3

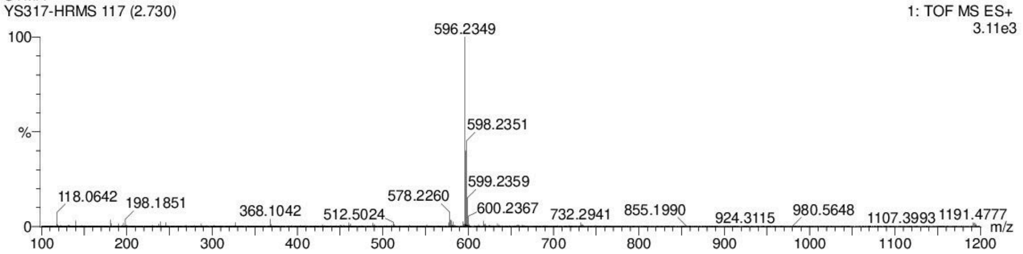

Minimum:				-1.5		
Maximum:		5.0	10.0	50.0		
Mass	Calc. Mass	mDa	PPM	DBE	i-FIT	Formula
596.2349	596.2350	-0.1	-0.2	14.5	0.8	C32 H39 N3 O4 S Cl
	596.2328	2.1	3.5	15.5	8.1	C32 H36 N3 O5 F Cl

Methyl 2-(4-((2-butyl-4-chloro-5-(hydroxy(2-(methoxymethoxy)phenyl)methyl)-1H-imidazol-1-yl)methyl) benzoyl)benzoate (4c).

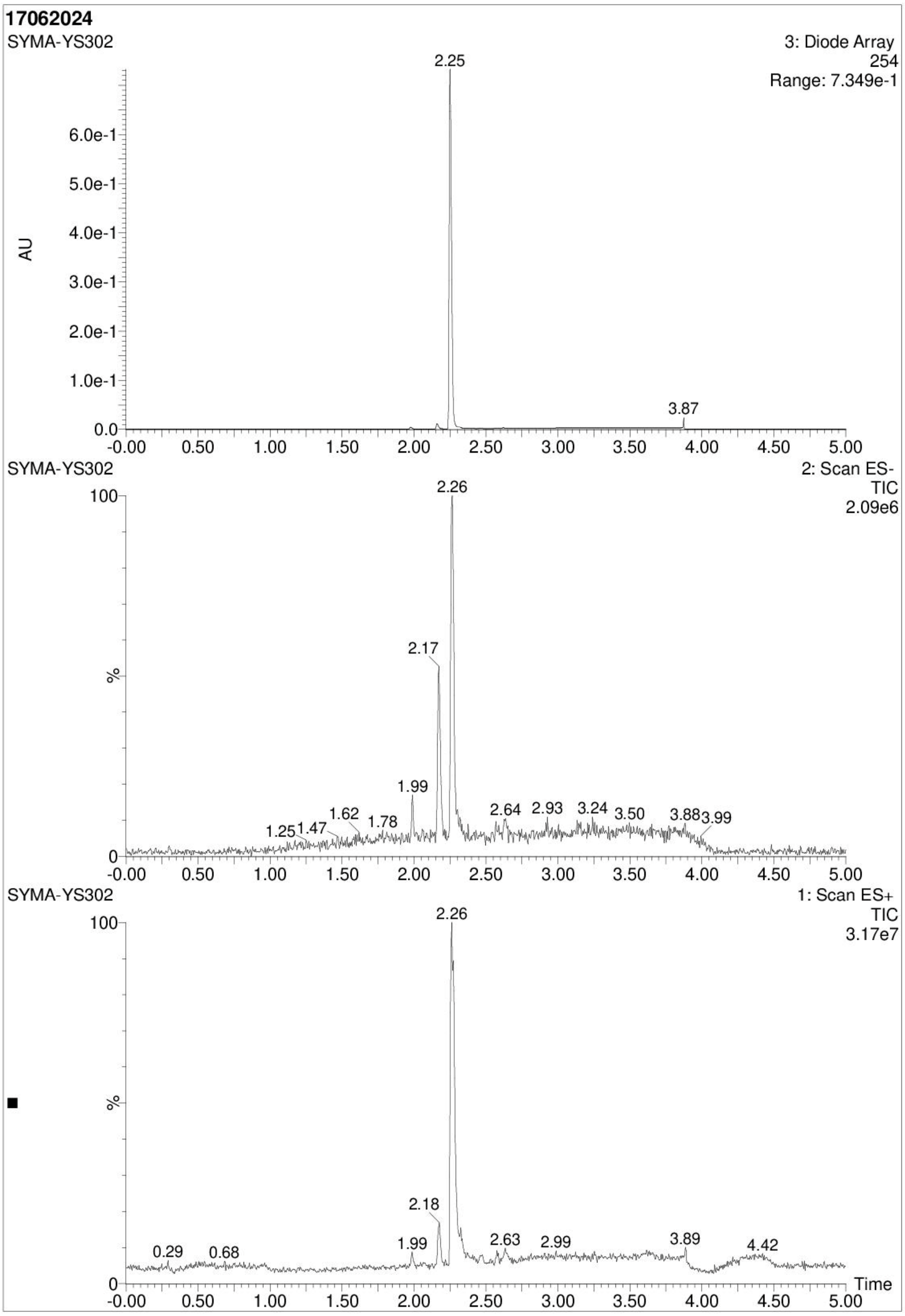

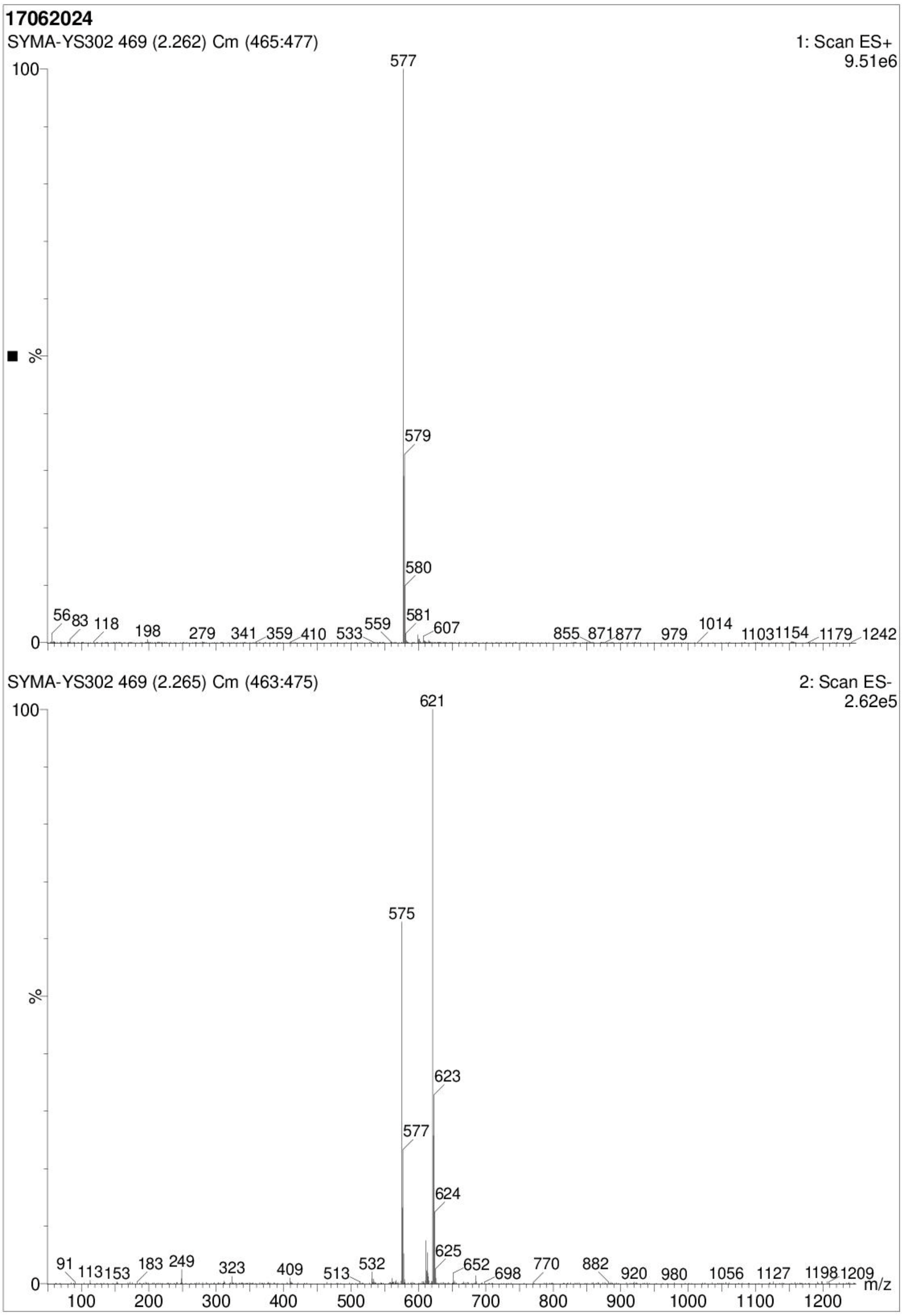
17062024
SYMA-YS302 469 (2.262) Cm (465:477)
1: Scan ES+
9.51e6
577
579
580
581
607
%
SYMA-YS302 469 (2.265) Cm (463:475)
2: Scan ES-
2.62e5
621
575
623
577
624
625
m/z

YS302-YSI126 1H 300MHz

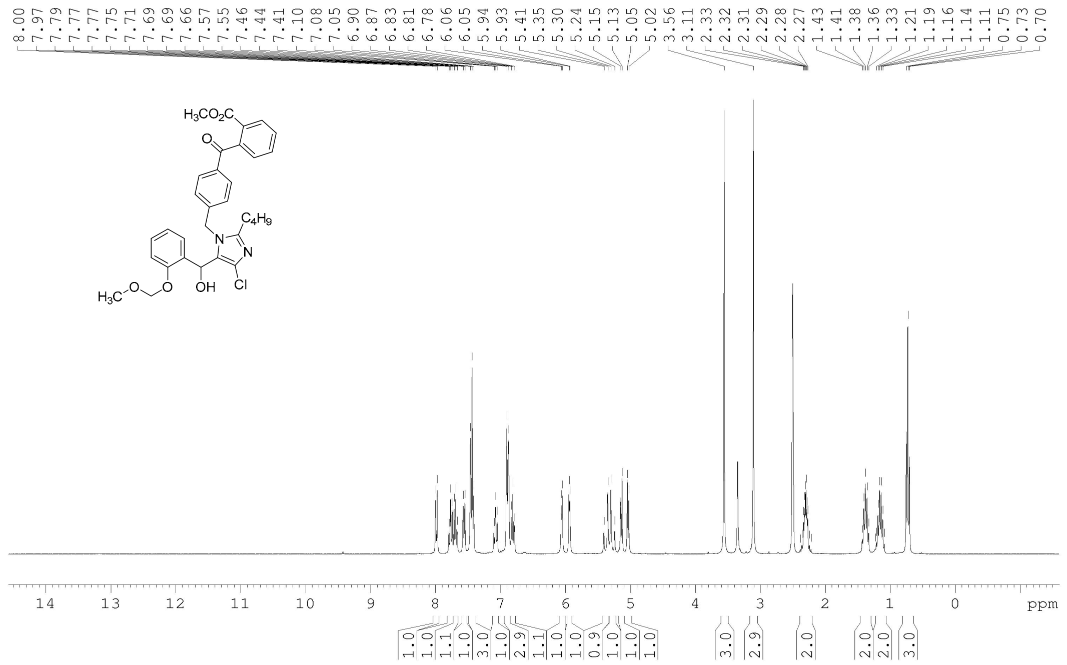

YS302-YSI126 13C

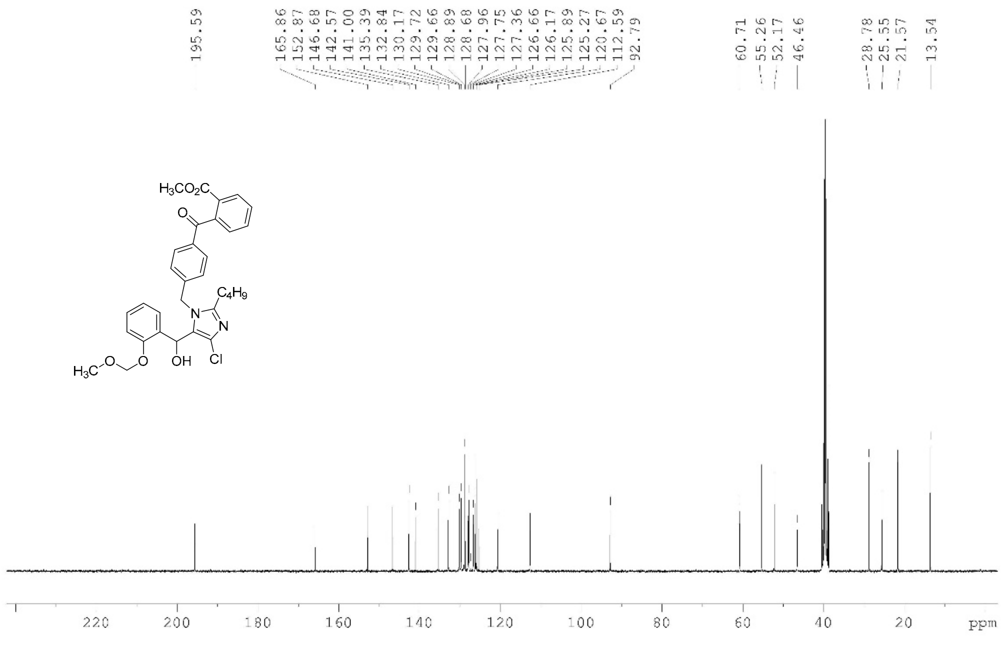

Single Mass Analysis
Tolerance = 5.0 mDa / DBE: min = -1.5, max = 50.0
Selected filters: None

Monoisotopic Mass, Even Electron Ions
2610 formula(e) evaluated with 4 results within limits (up to 1000 best isotopic matches for each mass)
Elements Used:
C: 0-33 H: 0-35 N: 0-2 O: 0-7 F: 0-1 S: 0-10 Cl: 0-1
SYMA
YS302-HRMS (2.541) Is (1.00,1.00) C32H34ClN2O6 1: TOF MS ES+ 5.18e12

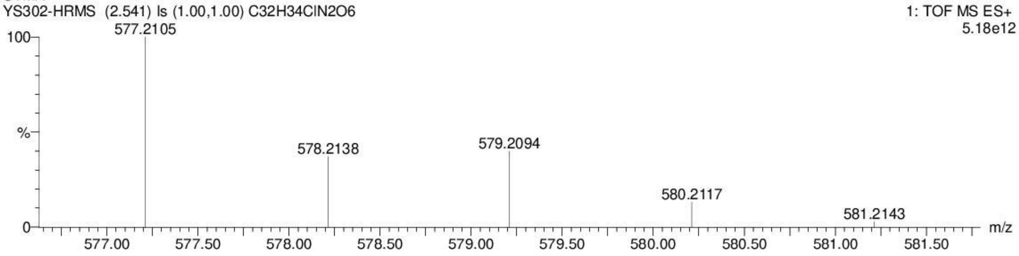

Minimum:				-1.5		
Maximum:		5.0	10.0	50.0		
Mass	Calc. Mass	mDa	PPM	DBE	i-FIT	Formula
577.2127	577.2105	2.2	3.8	16.5	0.0	C32 H34 N2 O6 Cl
	577.2117	1.0	1.7	12.5	5815957504.0	C29 H35 N2 O7 F Cl
	577.2092	3.5	6.1	16.5	8281605120.0	C33 H35 N2 O2 F S Cl
	577.2172	-4.5	-7.8	16.5	484350066688.0	C32 H34 N2 O5 F S

2-((1-((2'-(1H-Tetrazol-5-yl)-[1,1'-biphenyl]-4-yl)methyl)-2-butyl-4-chloro-1H-imidazol-5-yl)methyl)phenol (6).

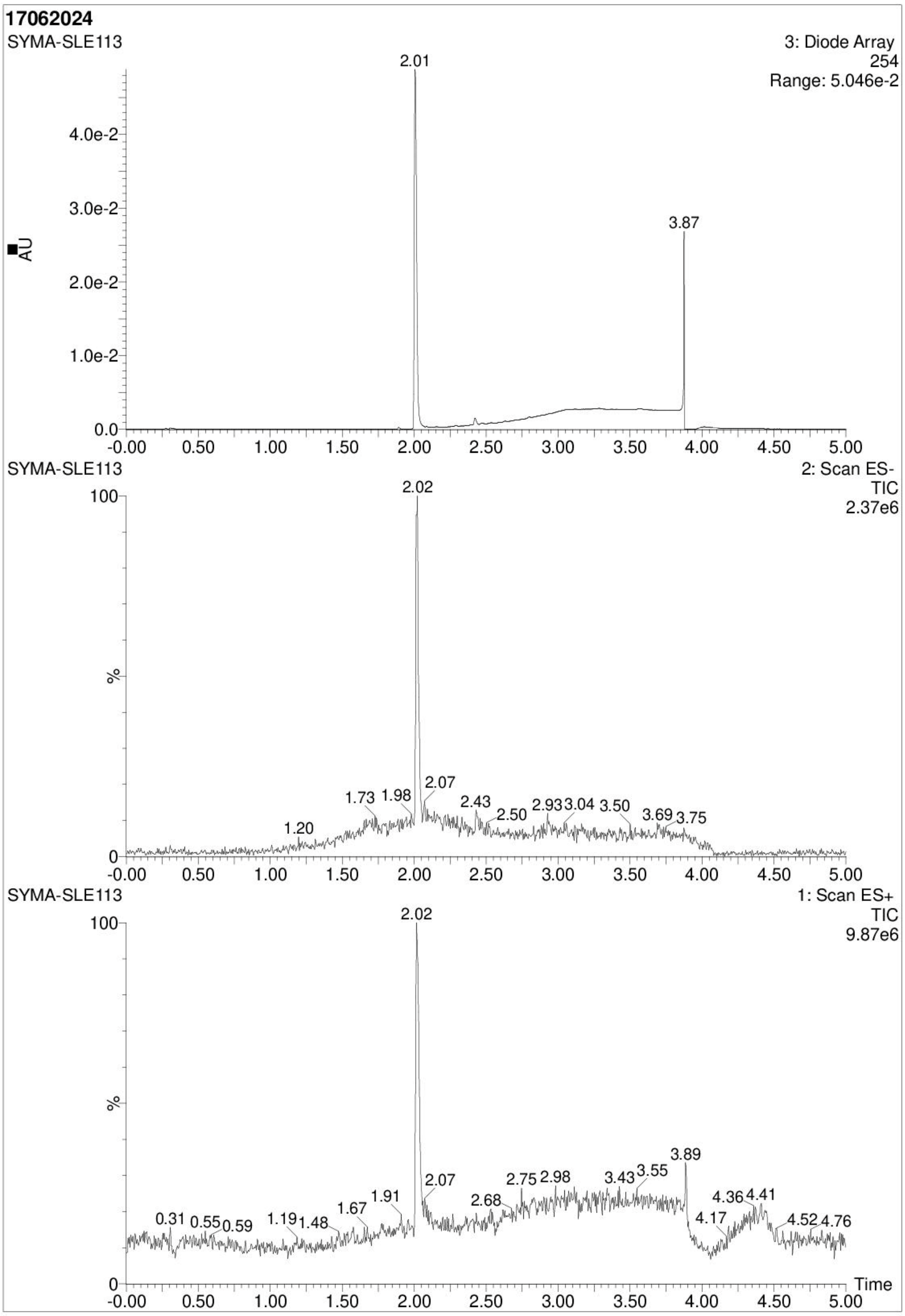

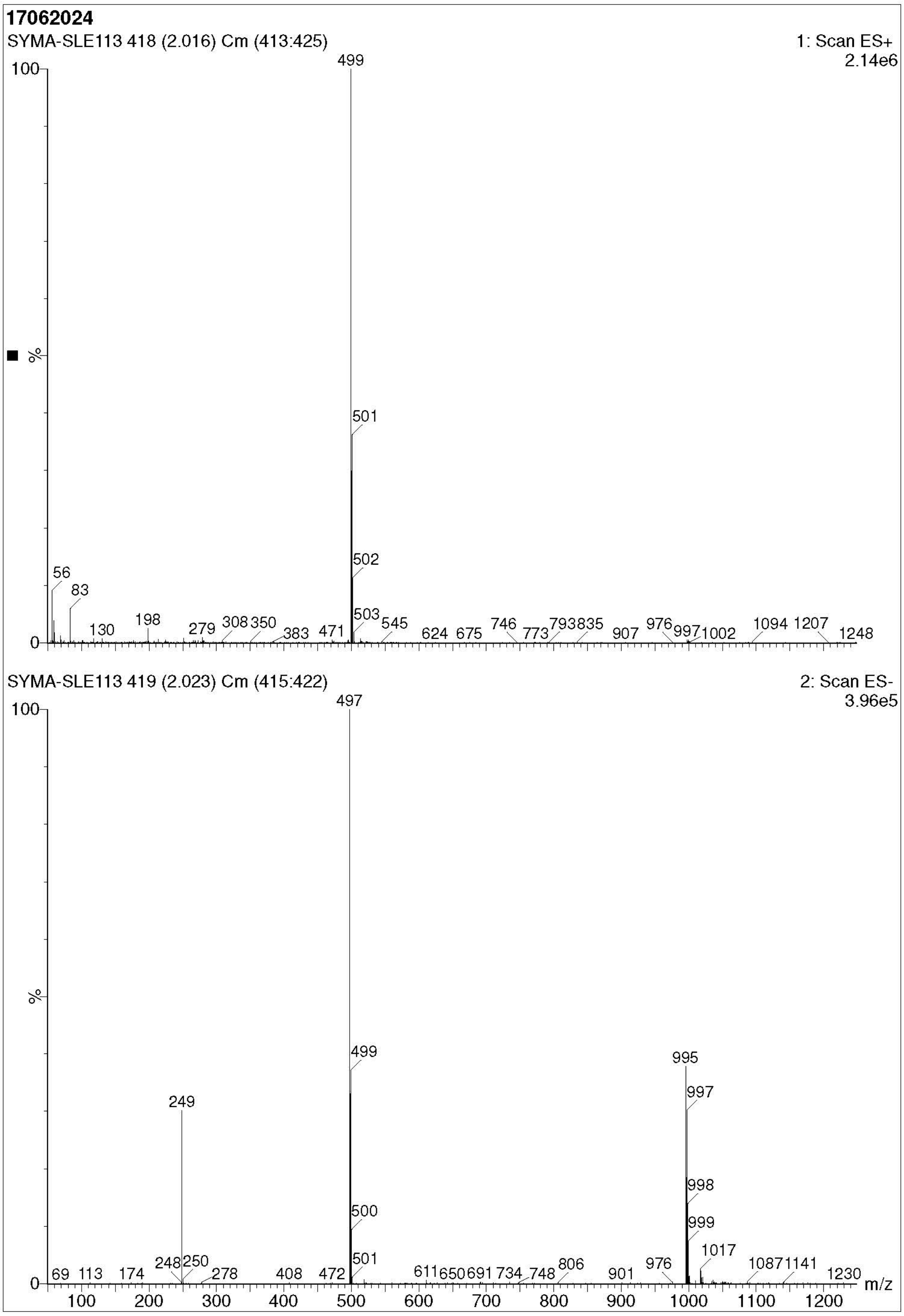
17062024
SYMA-SLE113 418 (2.016) Cm (413:425)
1: Scan ES+
2.14e6
100
%
0
499
501
502
56
83
130
198
279
308
350
383
471
503
545
624
675
746
773
793
835
907
976
997
1002
1094
1207
1248
SYMA-SLE113 419 (2.023) Cm (415:422)
2: Scan ES-
3.96e5
100
%
0
497
499
249
995
997
998
500
999
1017
501
69
113
174
248
250
278
408
472
611
650
691
734
748
806
901
976
1087
1141
1230
m/z
100
200
300
400
500
600
700
800
900
1000
1100
1200

SLE113-1H

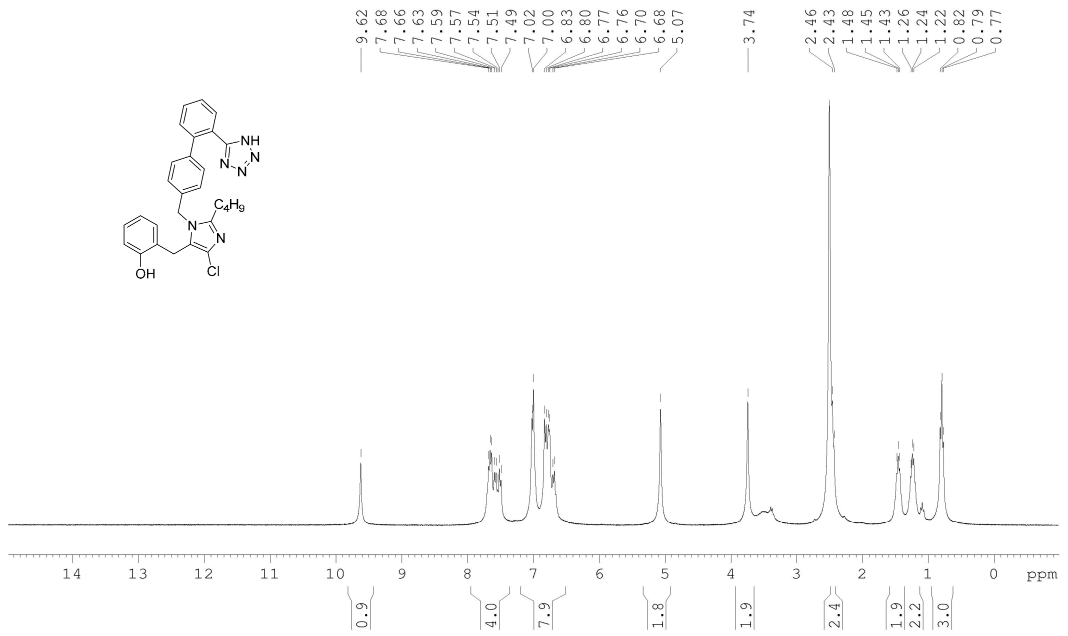

SLE113-13C

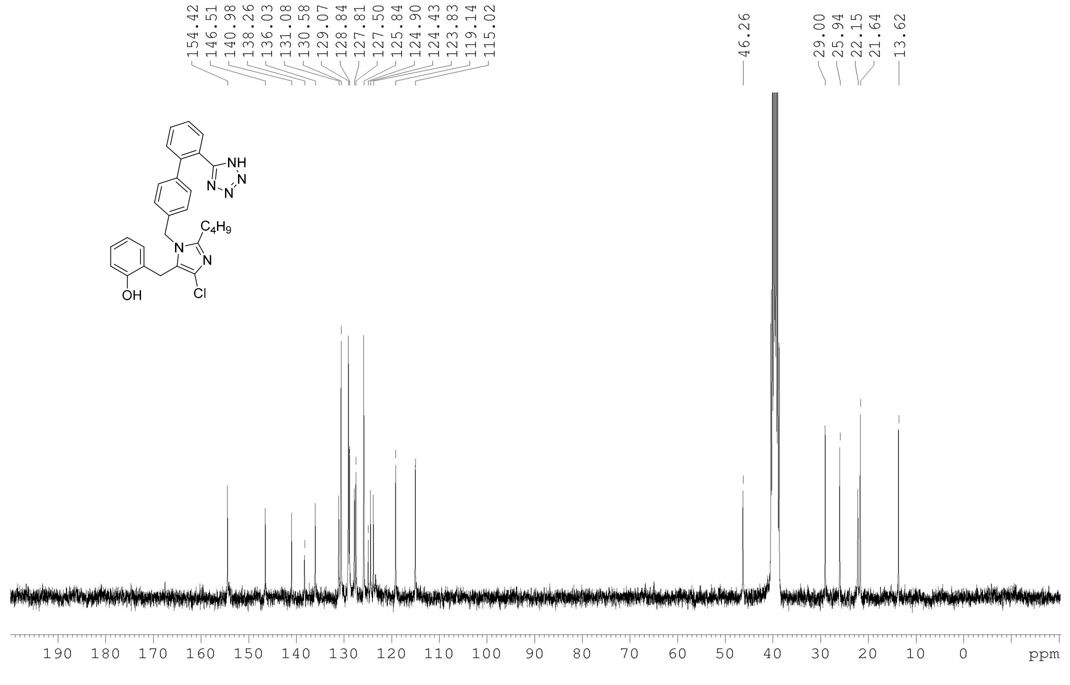

Elemental Composition Report

Single Mass Analysis

Tolerance = 5.0 mDa / DBE: min = -1.5, max = 50.0
Selected filters: None

Monoisotopic Mass, Even Electron Ions
42 formula(e) evaluated with 1 results within limits (up to 1000 best isotopic matches for each mass)
Elements Used:
C: 0-28 H: 0-28 N: 0-6 O: 0-1 F: 0-1 Cl: 0-1

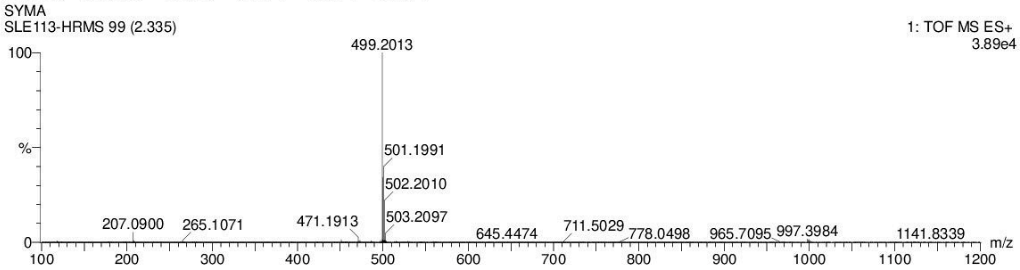

		mDa	PPM	DBE	i-FIT	Formula
Minimum:				-1.5		
Maximum:		5.0	10.0	50.0		

Mass	Calc. Mass	mDa	PPM	DBE	i-FIT	Formula
499.2013	499.2013	0.0	0.0	17.5	17.3	C28 H28 N6 O Cl

4'-((2-Butyl-4-chloro-5-(2-hydroxybenzyl)-1H-imidazol-1-yl)methyl)-[1,1'-biphenyl]-2-sulfonamide (7).

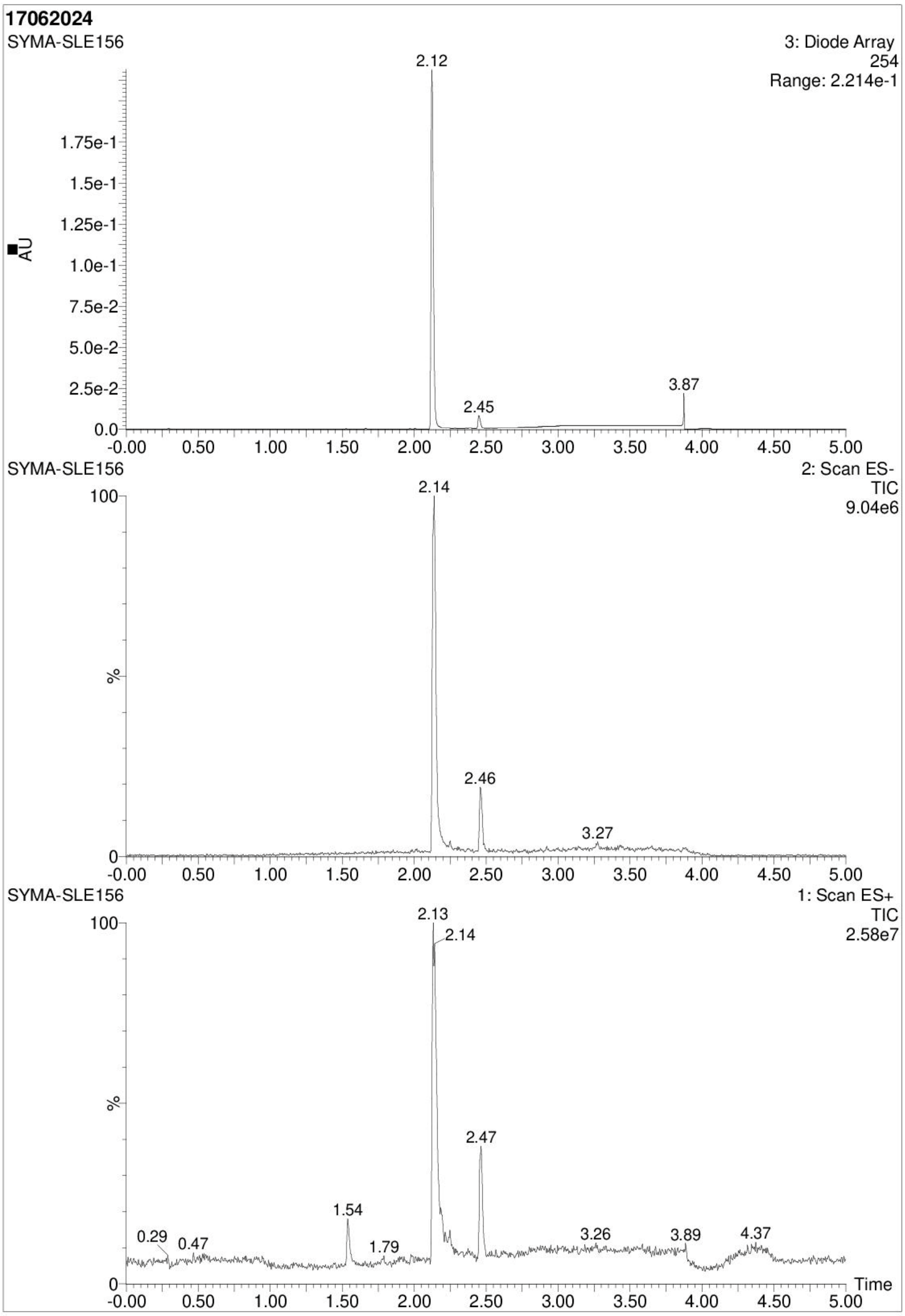

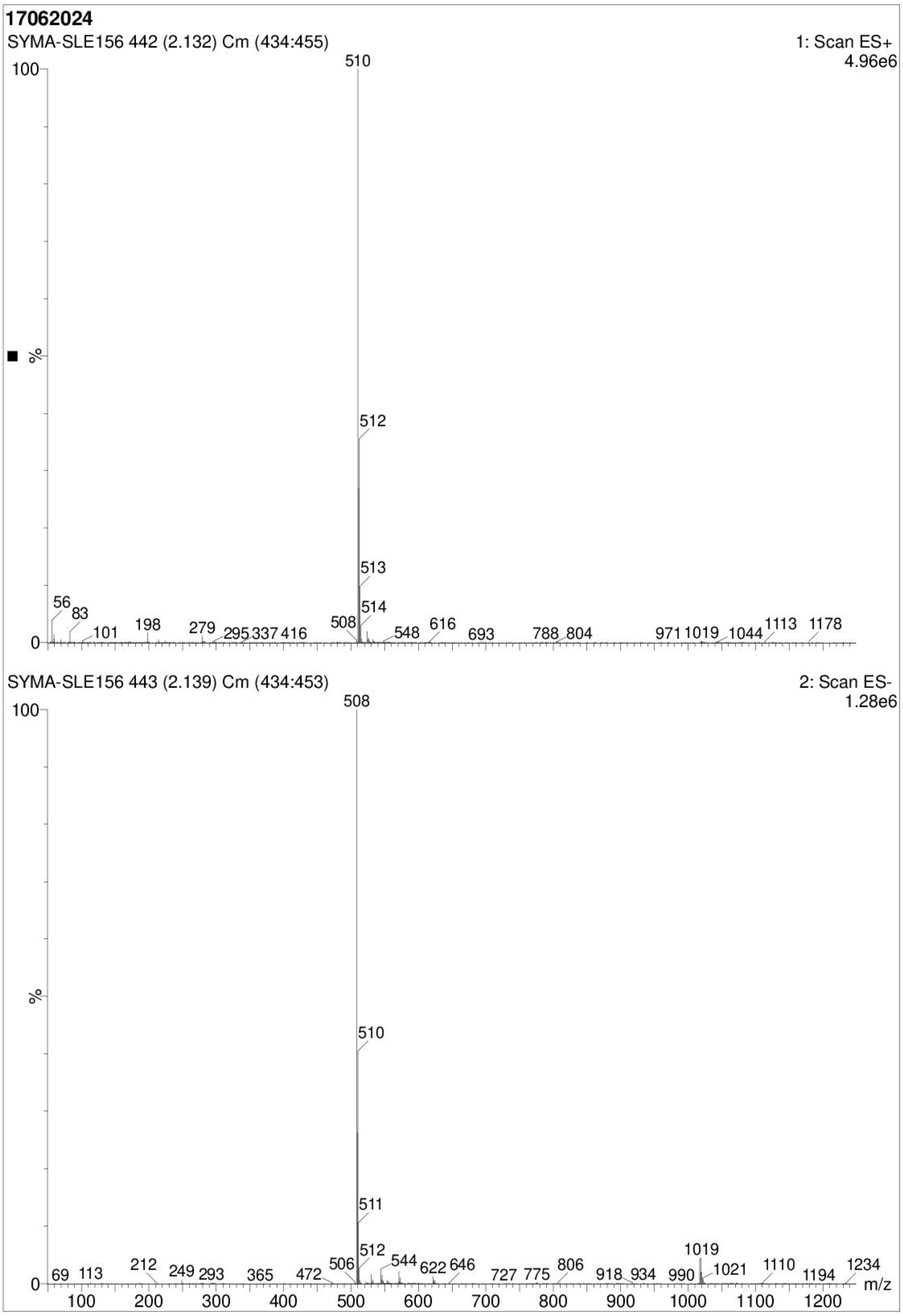
17062024
SYMA-SLE156 442 (2.132) Cm (434:455)
1: Scan ES+
4.96e6
100
%
0
510
512
513
514
508
56
83
101
198
279
295
337
416
548
616
693
788
804
971
1019
1044
1113
1178
SYMA-SLE156 443 (2.139) Cm (434:453)
2: Scan ES-
1.28e6
100
%
0
508
510
511
512
506
544
622
646
69
113
212
249
293
365
472
727
775
806
918
934
990
1019
1021
1110
1194
1234
m/z
100
200
300
400
500
600
700
800
900
1000
1100
1200

SLE156 1H 500 MHz

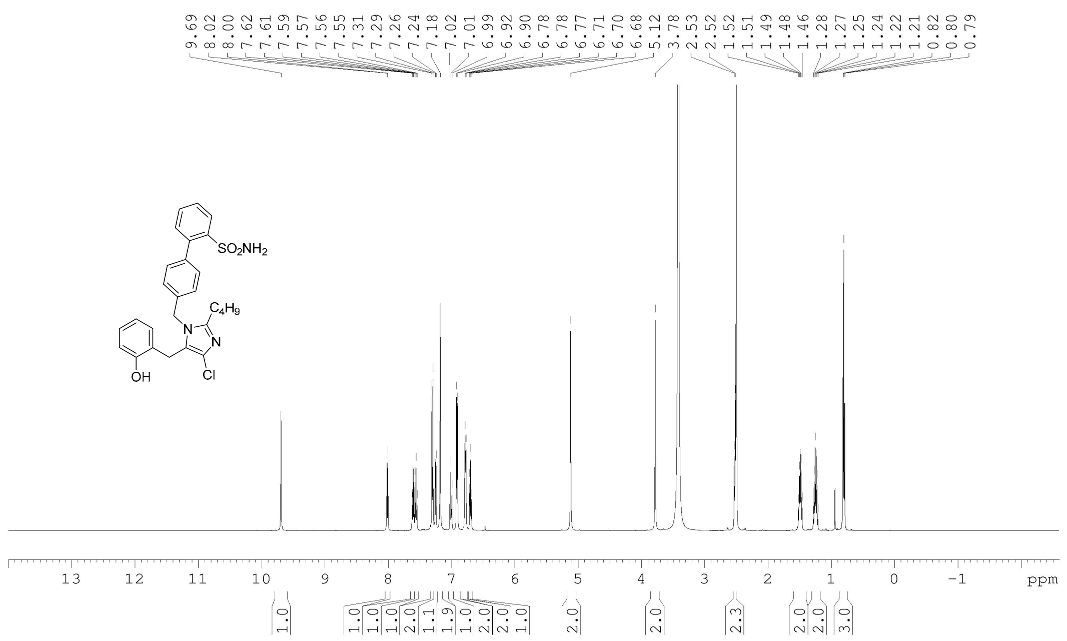

SLE156 13C 126 MHz

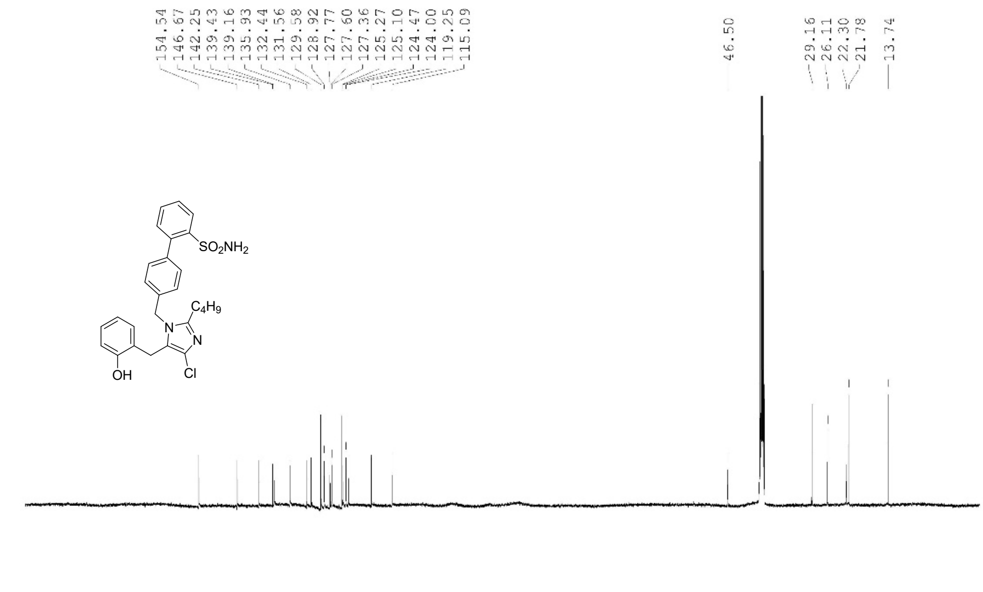

Single Mass Analysis
Tolerance = 5.0 mDa / DBE: min = -1.5, max = 50.0
Selected filters: None

Monoisotopic Mass, Even Electron Ions
109 formula(e) evaluated with 1 results within limits (up to 1000 best isotopic matches for each mass)
Elements Used:
C: 0-27 H: 0-29 N: 0-3 O: 0-3 F: 0-1 S: 0-1 Cl: 0-1

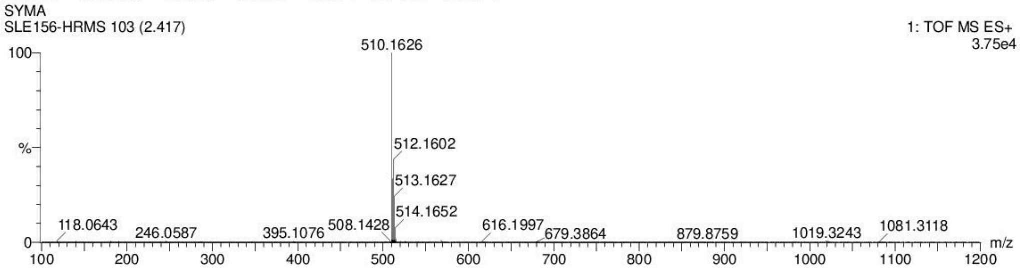

Minimum:				-1.5		
Maximum:		5.0	10.0	50.0		
Mass	Calc. Mass	mDa	PPM	DBE	i-FIT	Formula
510.1626	510.1618	0.8	1.6	14.5	8.8	C27 H29 N3 O3 S Cl

2-((1-((2'-(N-Benzoylsulfamoyl)-[1,1'-biphenyl]-4-yl)methyl)-2-butyl-4-chloro-1H-imidazol-5-yl)methyl) phenyl benzoate (8).

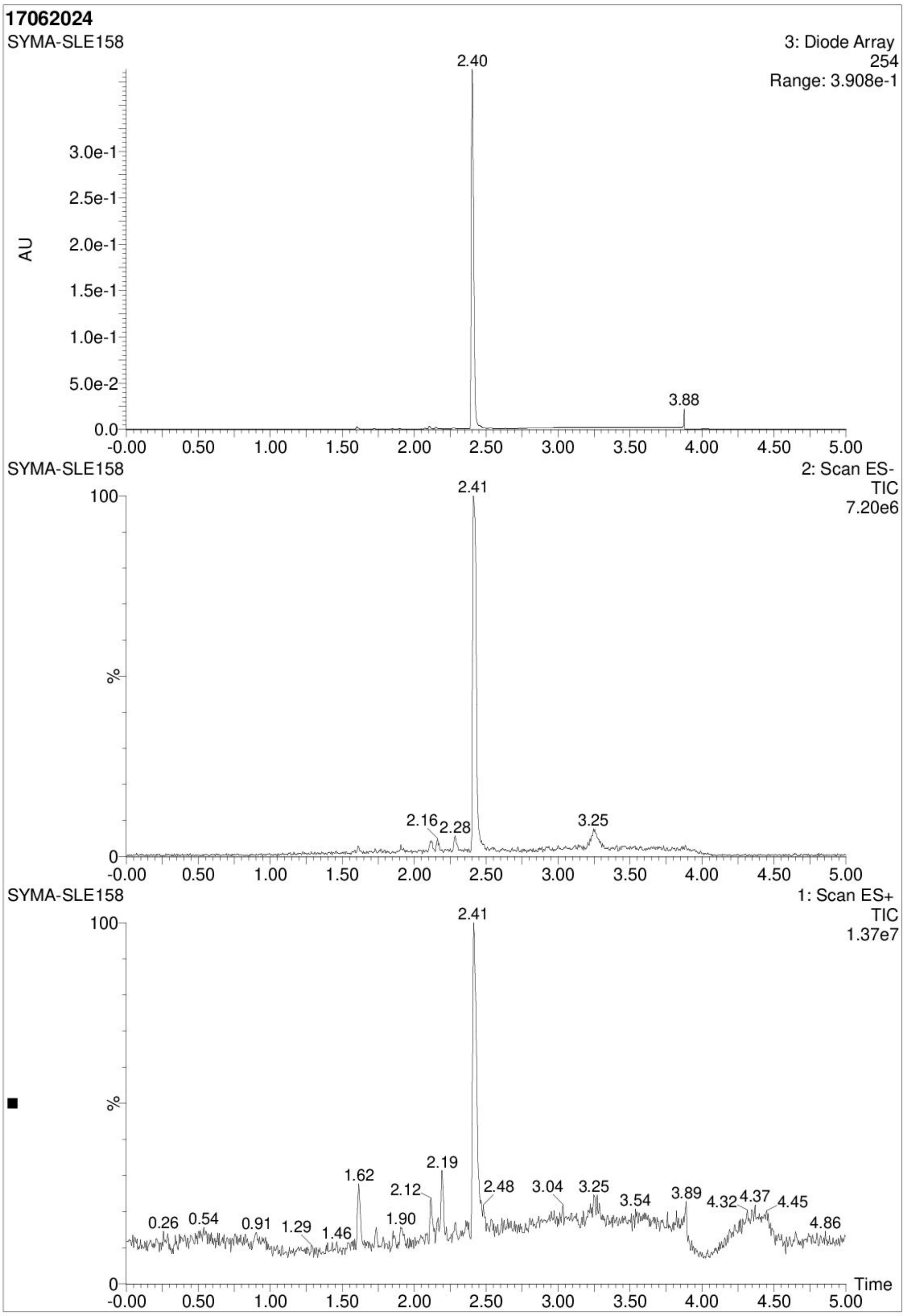

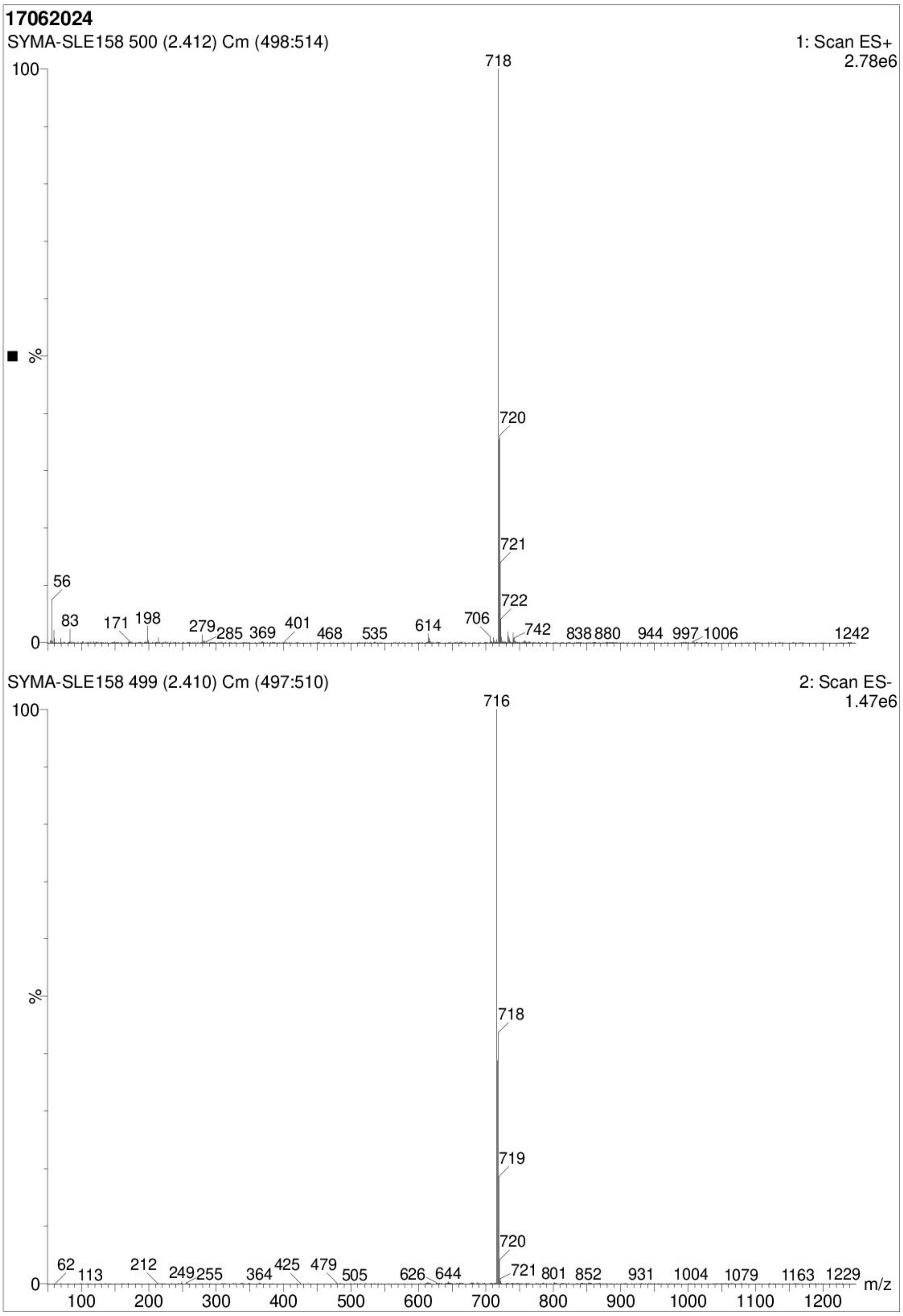
17062024
SYMA-SLE158 500 (2.412) Cm (498:514)
1: Scan ES+
2.78e6
718
720
721
722
56
83 171 198 279 285 369 401 468 535 614 706 742 838 880 944 997 1006 1242
SYMA-SLE158 499 (2.410) Cm (497:510)
2: Scan ES-
1.47e6
716
718
719
720
62 113 212 249 255 364 425 479 505 626 644 721 801 852 931 1004 1079 1163 1229
%
100
0
m/z
100 200 300 400 500 600 700 800 900 1000 1100 1200

SLE158 1H

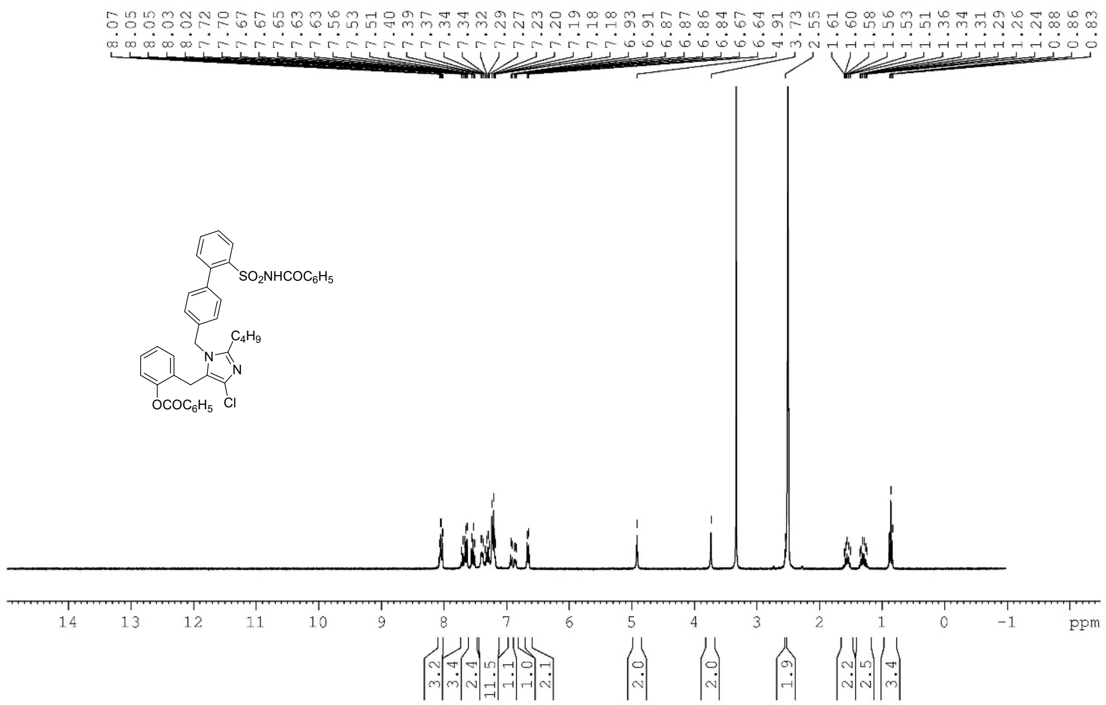

SLE158 13C

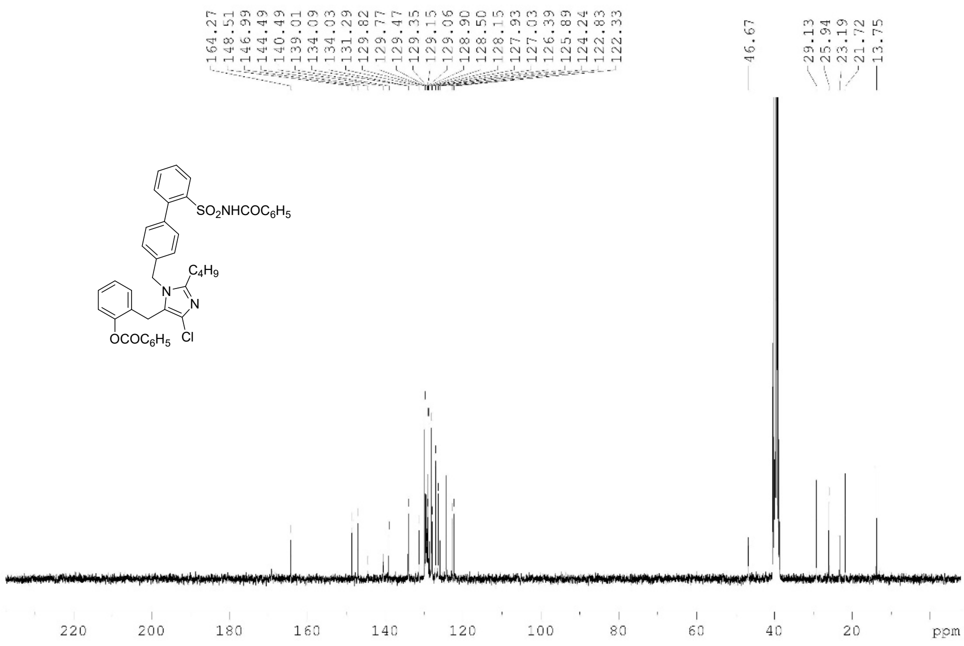

Single Mass Analysis
Tolerance = 5.0 mDa / DBE: min = -1.5, max = 50.0
Selected filters: None

Monoisotopic Mass, Even Electron Ions
152 formula(e) evaluated with 1 results within limits (up to 1000 best isotopic matches for each mass)
Elements Used:
C: 0-41 H: 0-37 N: 0-3 O: 0-5 F: 0-1 S: 0-1 Cl: 0-1

SYMA
SLE158-HRMS 123 (2.854) 1: TOF MS ES+ 1.92e4

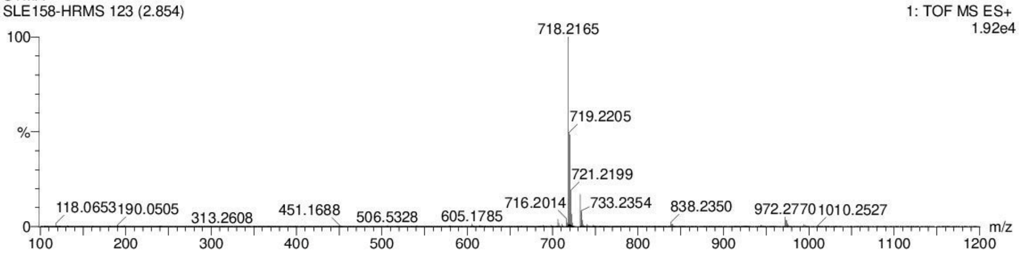

Minimum:				-1.5		
Maximum:		5.0	10.0	50.0		
Mass	Calc. Mass	mDa	PPM	DBE	i-FIT	Formula
718.2165	718.2142	2.3	3.2	24.5	4.2	C41 H37 N3 O5 S Cl

***N*-((4'-((2-butyl-4-chloro-5-(2-hydroxybenzyl)-1*H*-imidazol-1-yl)methyl)-[1,1'-biphenyl]-2-yl)sulfonyl)benzamide (9).**

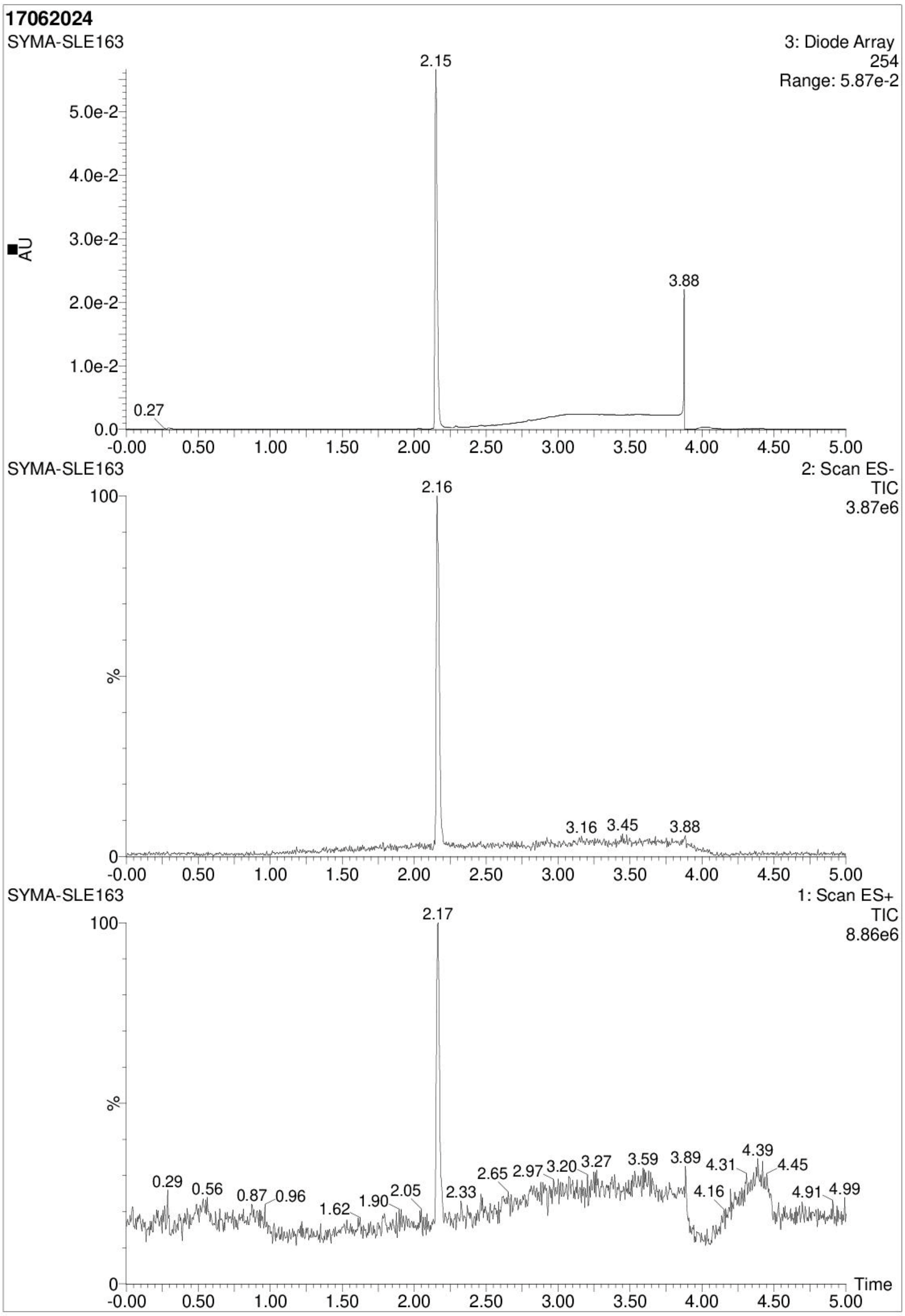

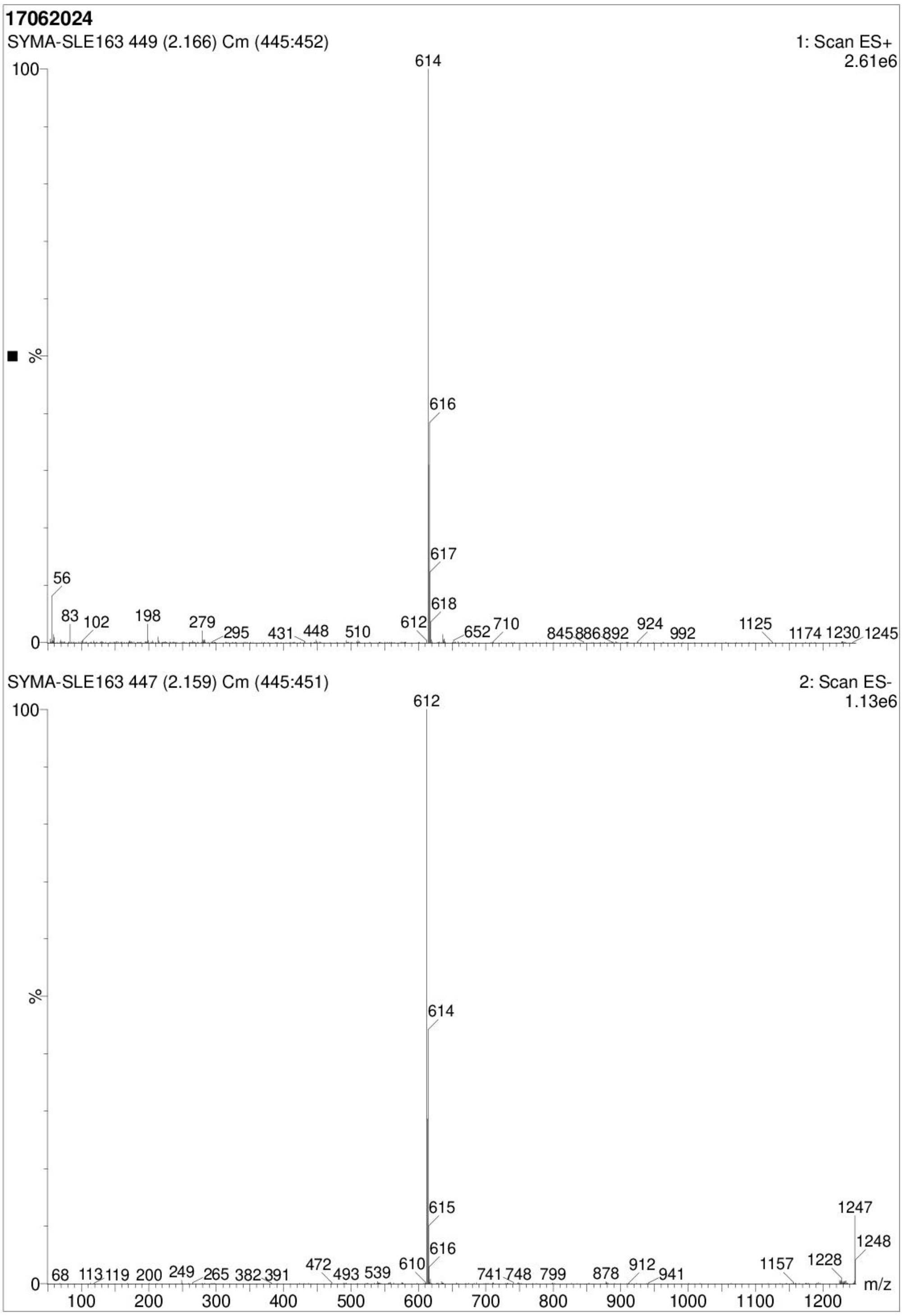
17062024
SYMA-SLE163 449 (2.166) Cm (445:452)
1: Scan ES+
2.61e6
614
616
617
618
56
83 102
198
279
295
431 448
510
612
652 710
845 886 892
924
992
1125
1174 1230 1245
SYMA-SLE163 447 (2.159) Cm (445:451)
2: Scan ES-
1.13e6
612
614
615
616
610
68 113 119 200 249 265 382 391 472 493 539
741 748 799 878 912 941
1157 1228
1247
1248
m/z
%
100
0

SLE163 1H 300MHz

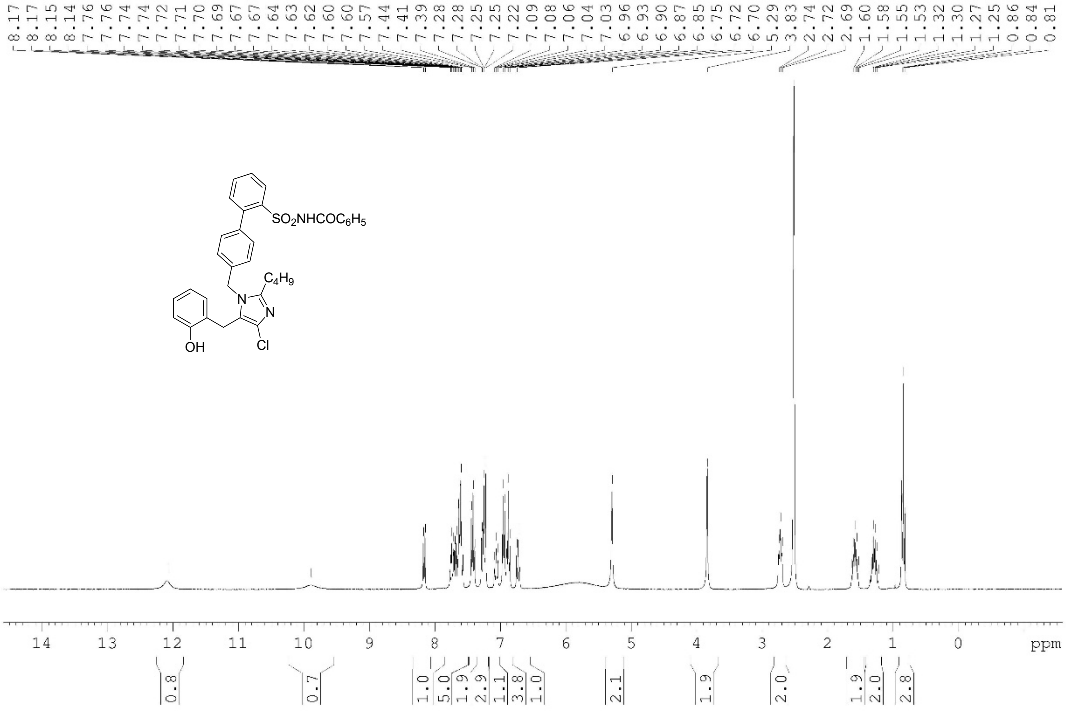

SLE163 13C

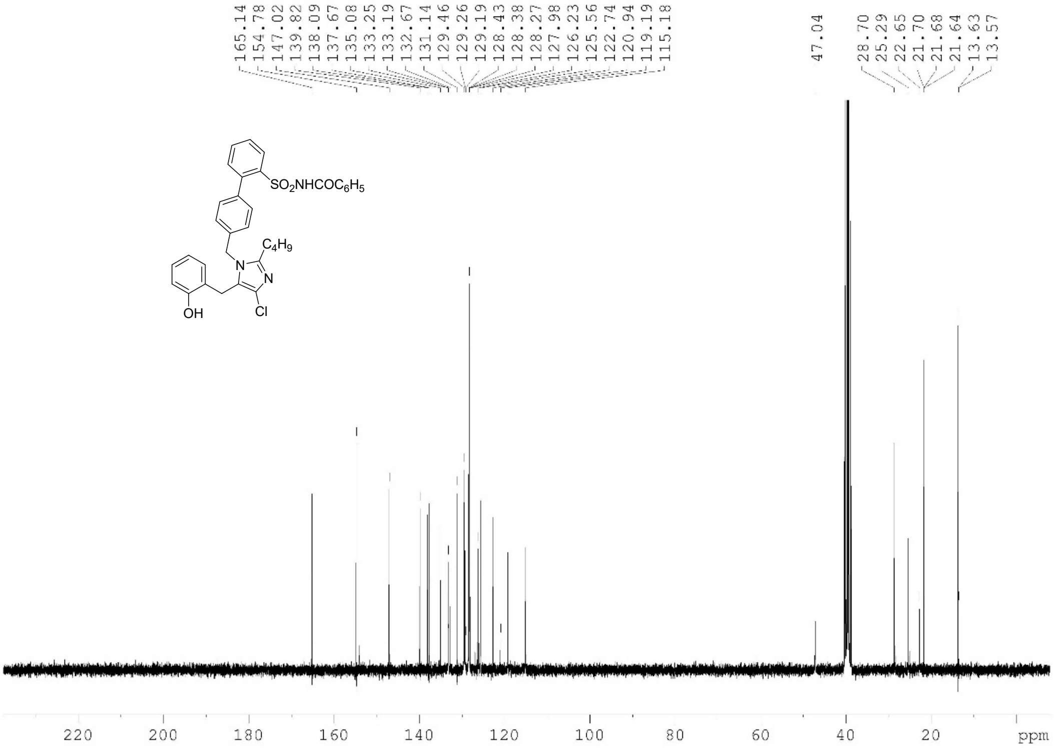

Single Mass Analysis

Tolerance = 5.0 mDa / DBE: min = -1.5, max = 50.0
Selected filters: None

Monoisotopic Mass, Even Electron Ions
123 formula(e) evaluated with 1 results within limits (up to 1000 best isotopic matches for each mass)
Elements Used:
C: 0-34 H: 0-33 N: 0-3 O: 0-4 F: 0-1 S: 0-1 Cl: 0-1

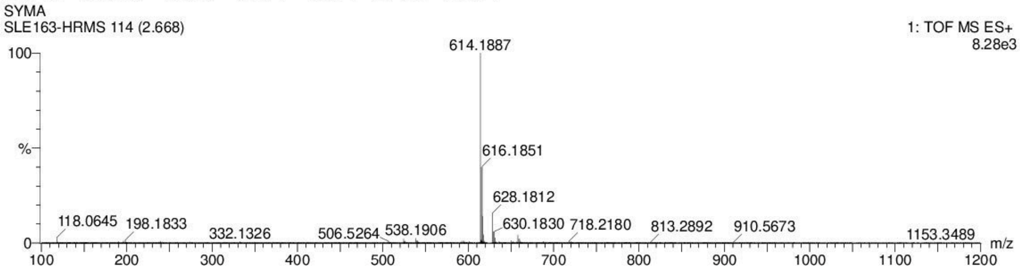

		mDa	PPM	DBE	i-FIT	Formula
Minimum:				-1.5		
Maximum:		5.0	10.0	50.0		
Mass	Calc. Mass	mDa	PPM	DBE	i-FIT	Formula
614.1887	614.1880	0.7	1.1	19.5	18.6	C34 H33 N3 O4 S Cl

N-(tert-Butyl)-4'-((2-butyl-4-chloro-5-(2-methoxybenzoyl)-1H-imidazol-1-yl)methyl)-[1,1'-biphenyl]-2-sulfonamide (10).

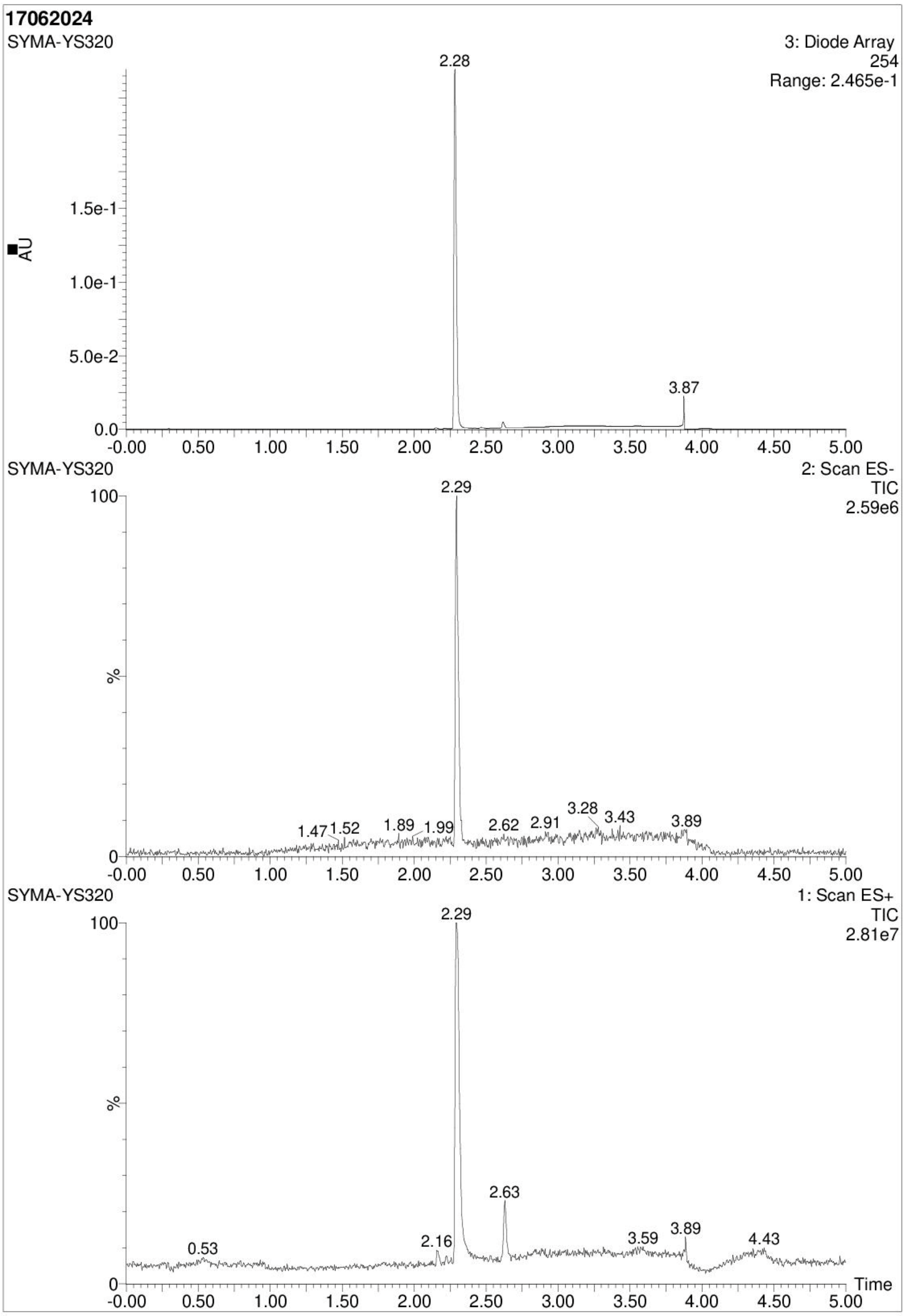

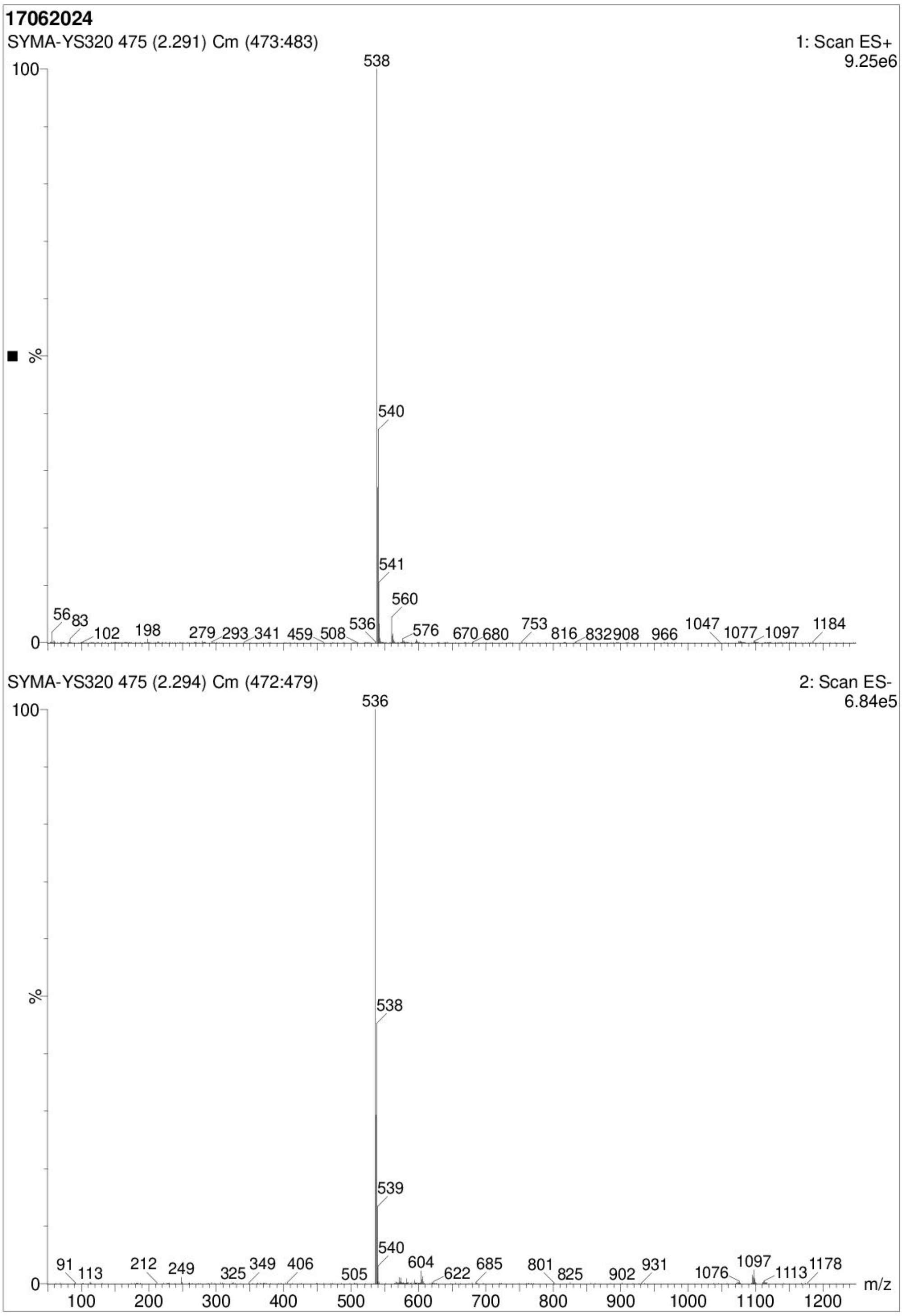

17062024
SYMA-YS320 475 (2.291) Cm (473:483)
1: Scan ES+
9.25e6
538
540
541
560
56
83
102
198
279
293
341
459
508
536
576
670
680
753
816
832
908
966
1047
1077
1097
1184
SYMA-YS320 475 (2.294) Cm (472:479)
2: Scan ES-
6.84e5
536
538
539
540
91
113
212
249
325
349
406
505
604
622
685
801
825
902
931
1076
1097
1113
1178
100
%
0
m/z
100
200
300
400
500
600
700
800
900
1000
1100
1200

YS320 1H

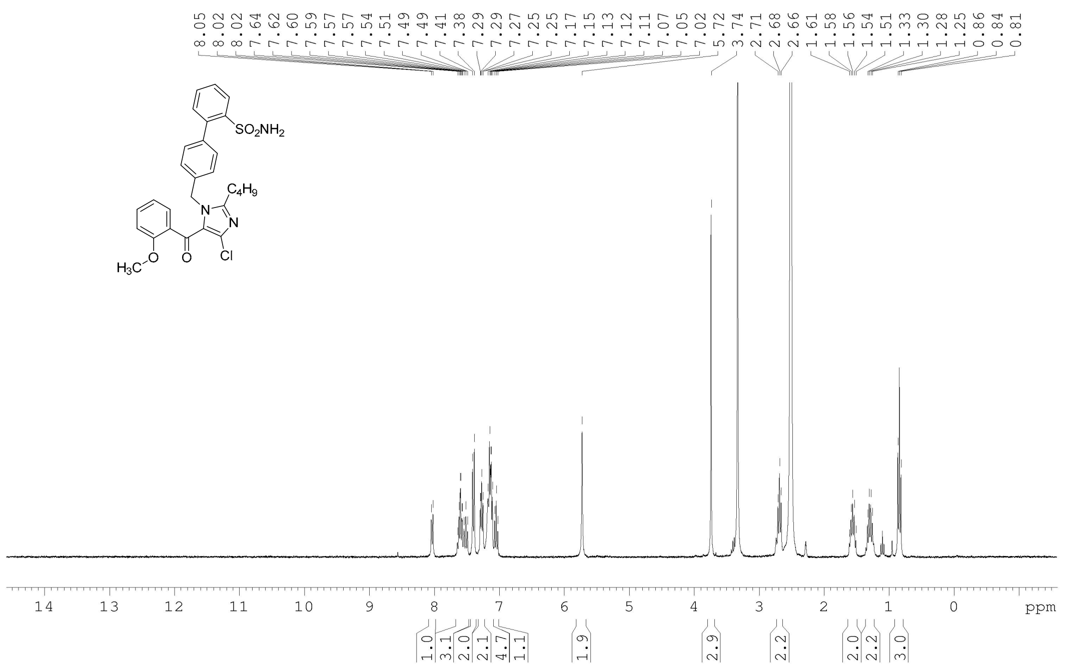

YS320 13C

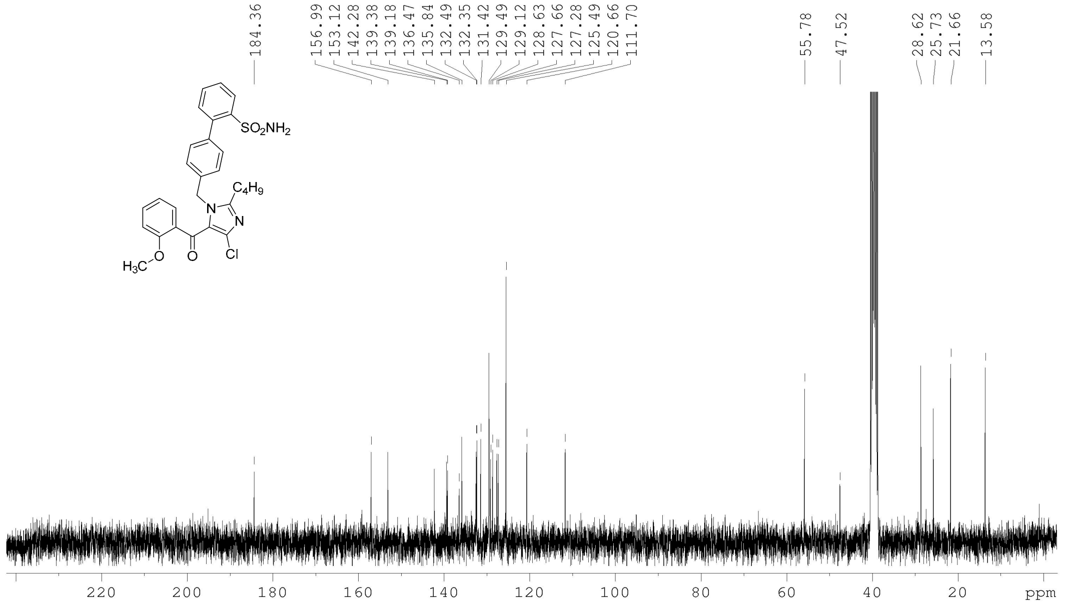

Elemental Composition Report **Page 1**

Single Mass Analysis
Tolerance = 5.0 mDa / DBE: min = -1.5, max = 50.0
Selected filters: None

Monoisotopic Mass, Even Electron Ions
141 formula(e) evaluated with 1 results within limits (up to 1000 best isotopic matches for each mass)
Elements Used:
C: 0-28 H: 0-29 N: 0-3 O: 0-4 F: 0-1 S: 0-1 Cl: 0-1

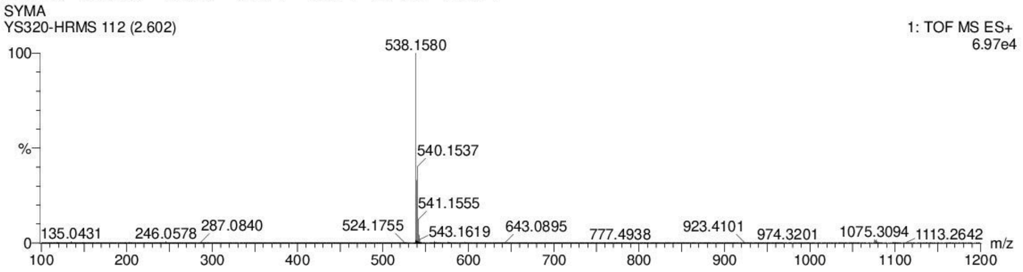

Minimum:				-1.5		
Maximum:		5.0	10.0	50.0		
Mass	Calc. Mass	mDa	PPM	DBE	i-FIT	Formula
538.1580	538.1567	1.3	2.4	15.5	44.9	C28 H29 N3 O4 S Cl

N-((4'-((2-Butyl-4-chloro-5-(2-methoxybenzoyl)-1H-imidazol-1-yl)methyl)-[1,1'-biphenyl]-2-yl)sulfonyl) benzamide (11).

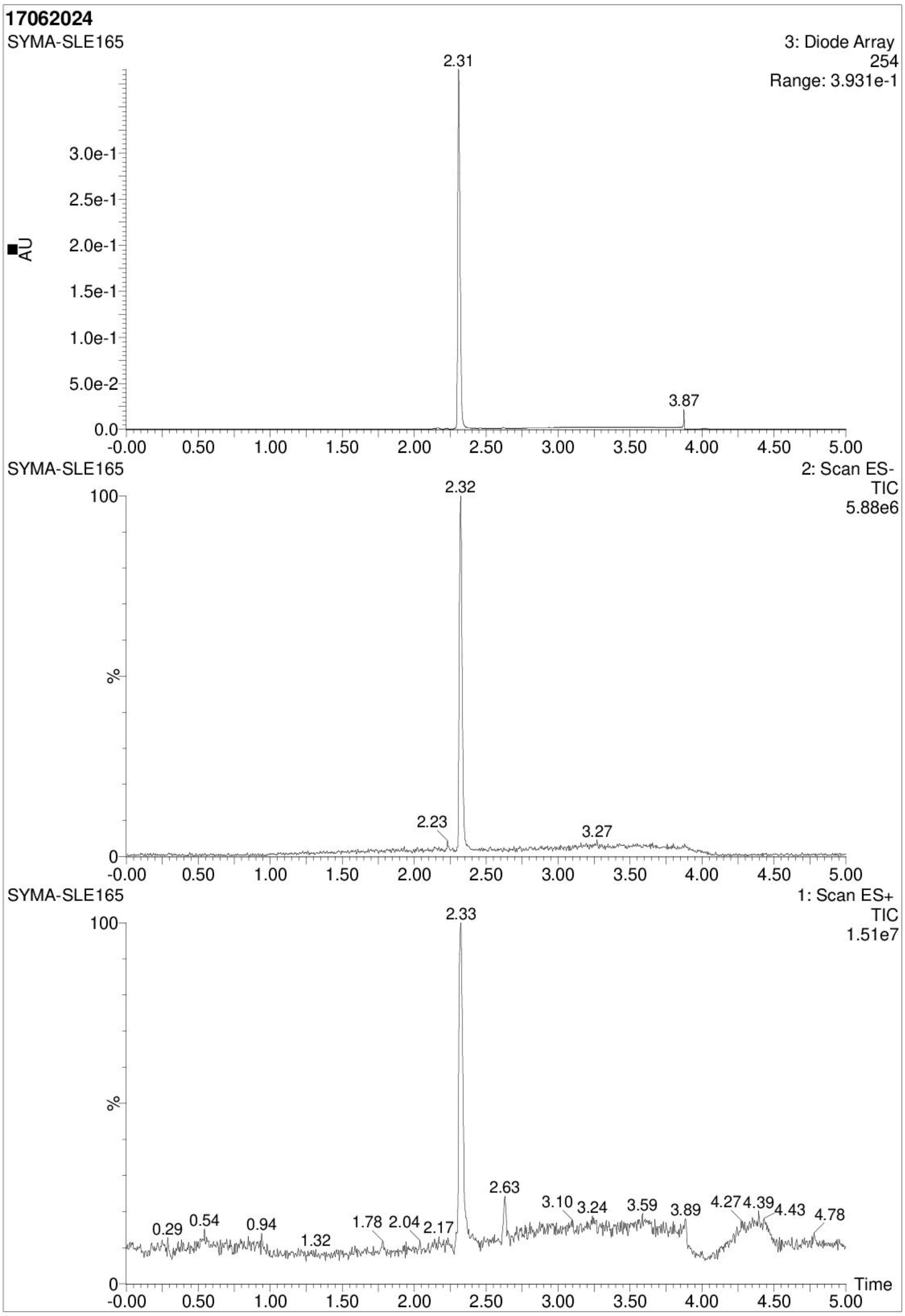

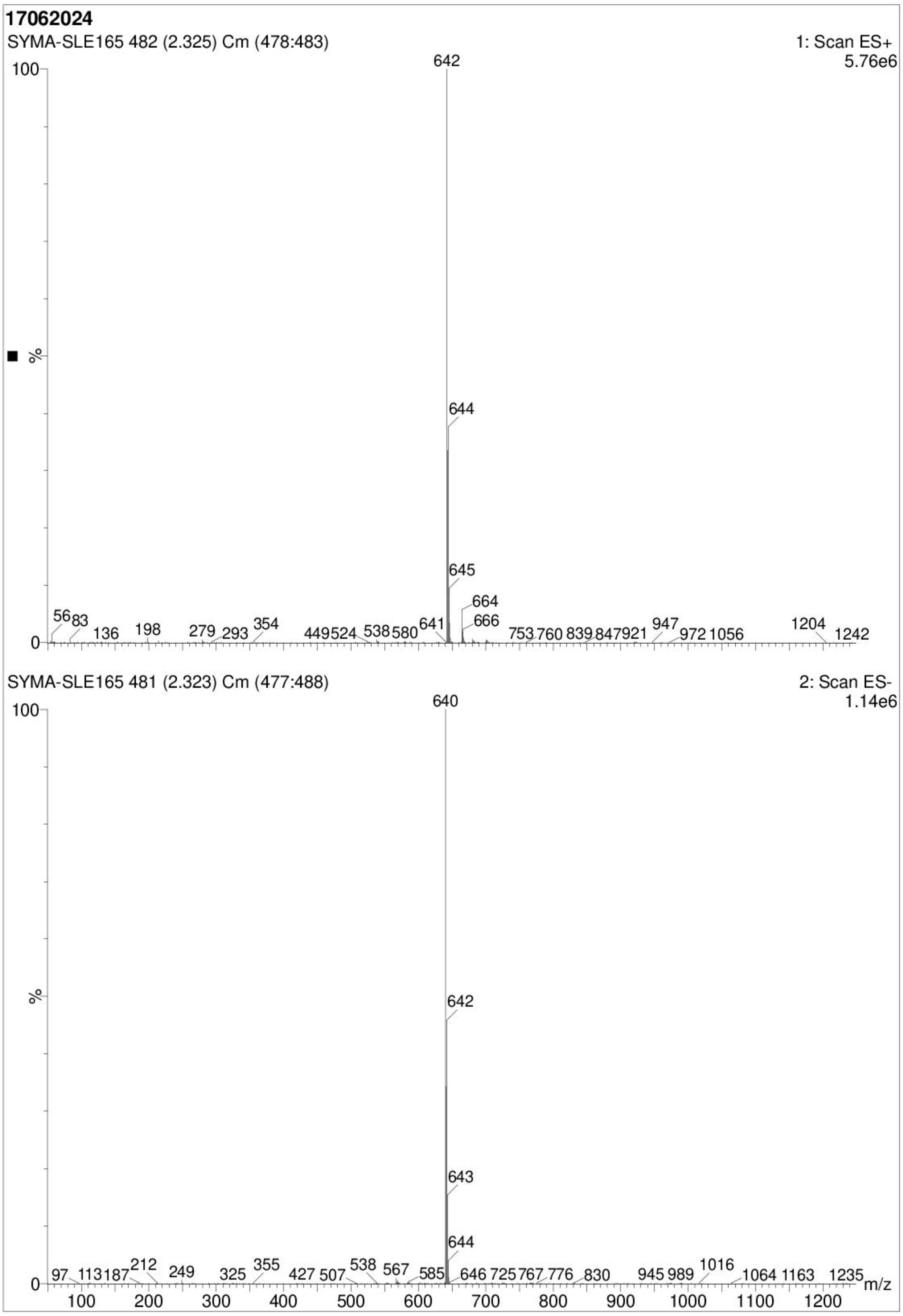
17062024
SYMA-SLE165 482 (2.325) Cm (478:483)
1: Scan ES+
5.76e6
100
%
0
642
644
645
664
666
56
83
136
198
279
293
354
449
524
538
580
641
753
760
839
847
921
947
972
1056
1204
1242
SYMA-SLE165 481 (2.323) Cm (477:488)
2: Scan ES-
1.14e6
640
642
643
644
97
113
187
212
249
325
355
427
507
538
567
585
646
725
767
776
830
945
989
1016
1064
1163
1235
m/z
100
200
300
400
500
600
700
800
900
1000
1100
1200

SLE165 DMSO

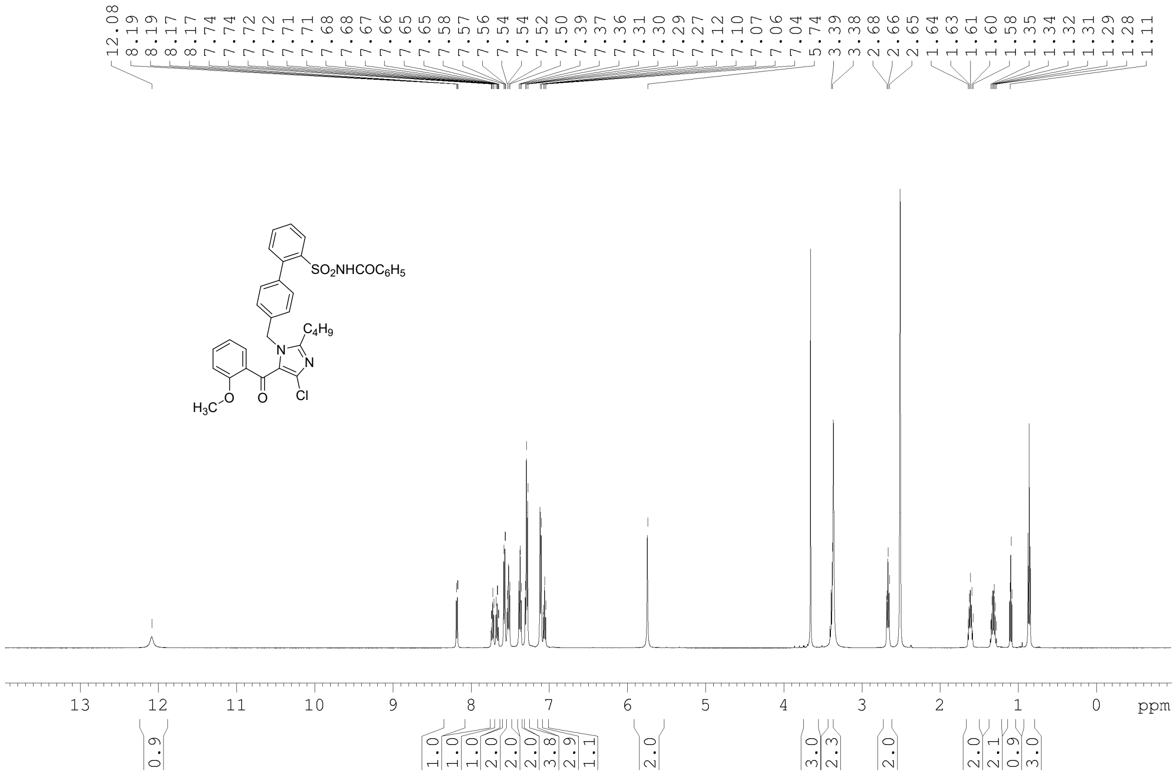

SLE165 13C 126 MHz

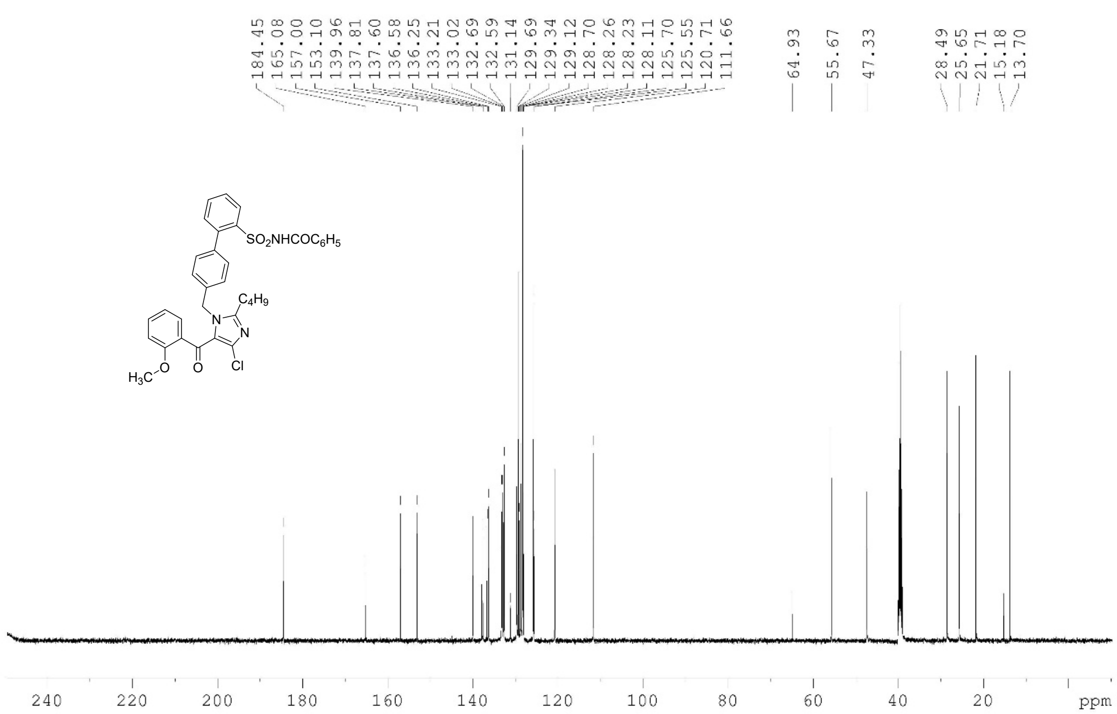

Elemental Composition Report **Page 1**

Single Mass Analysis
Tolerance = 5.0 mDa / DBE: min = -1.5, max = 50.0
Selected filters: None

Monoisotopic Mass, Even Electron Ions
154 formula(e) evaluated with 1 results within limits (up to 1000 best isotopic matches for each mass)
Elements Used:
C: 0-35 H: 0-33 N: 0-3 O: 0-5 F: 0-1 S: 0-1 Cl: 0-1

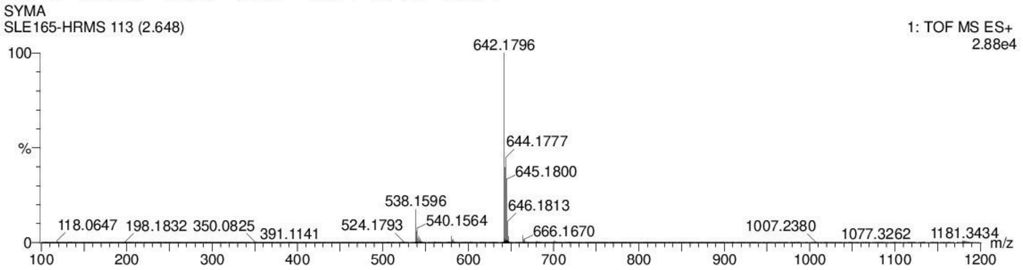

		mDa	PPM	DBE	i-FIT	Formula
Minimum:				-1.5		
Maximum:		5.0	10.0	50.0		

Mass	Calc. Mass	mDa	PPM	DBE	i-FIT	Formula
642.1796	642.1829	-3.3	-5.1	20.5	14.0	C35 H33 N3 O5 S Cl

N-((4'-((2-Butyl-4-chloro-5-(2-hydroxybenzoyl)-1H-imidazol-1-yl)methyl)-[1,1'-biphenyl]-2-yl)sulfonyl) benzamide (12).

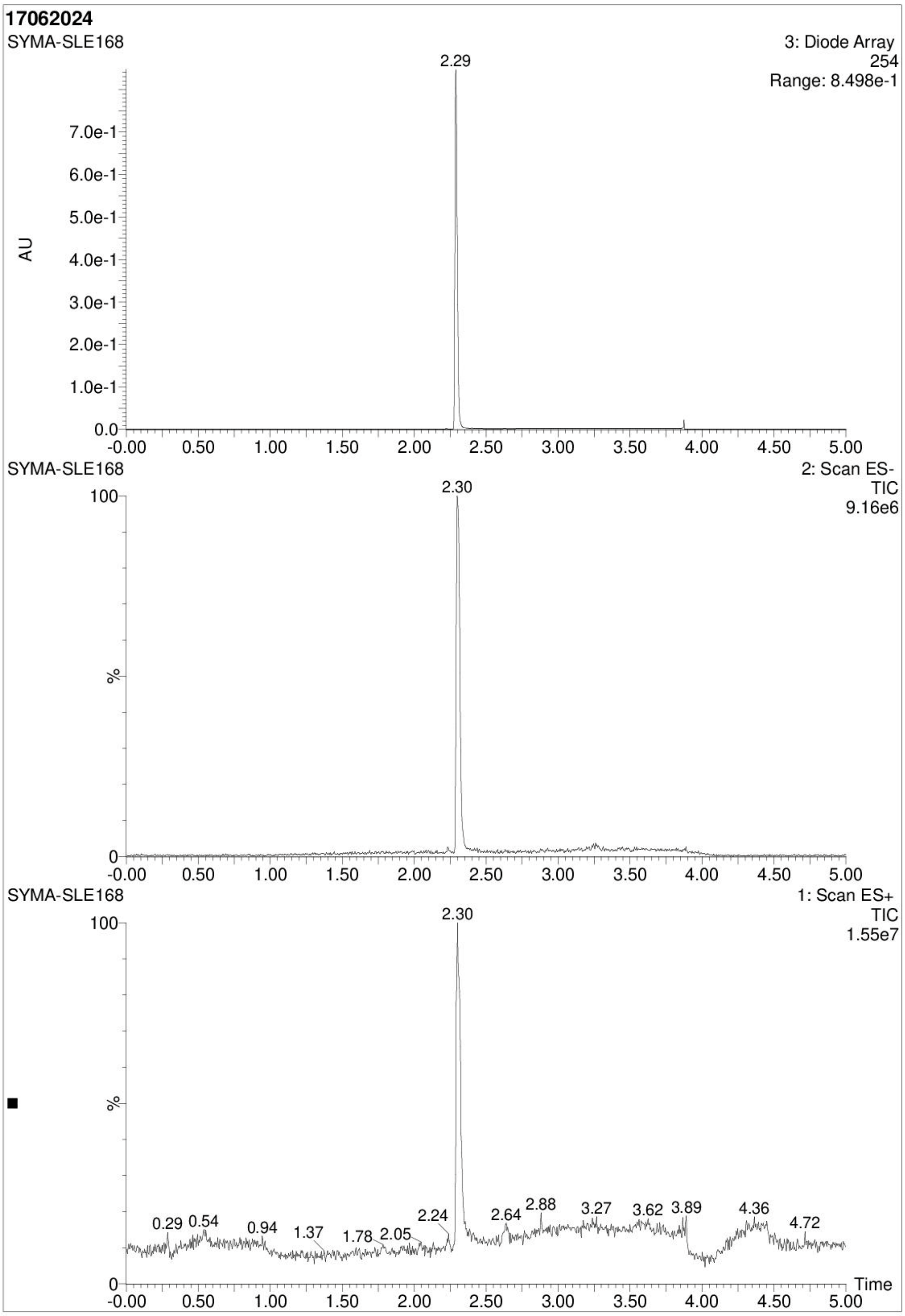

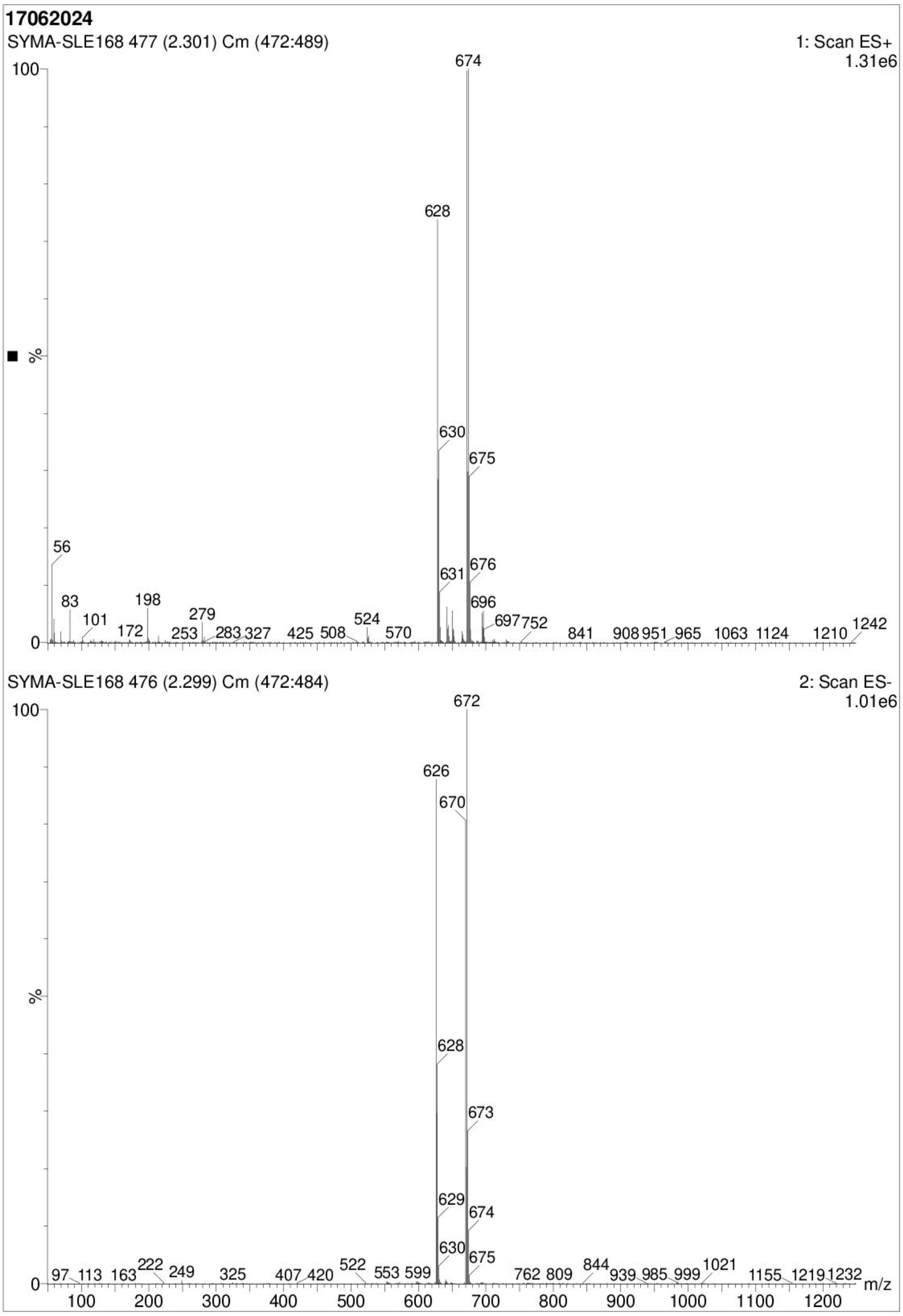

17062024
SYMA-SLE168 477 (2.301) Cm (472:489)
1: Scan ES+
1.31e6
674
628
630
675
56
676
631
83
198
696
101
279
697
752
172
253
283
327
425
508
524
570
841
908
951
965
1063
1124
1210
1242
%
SYMA-SLE168 476 (2.299) Cm (472:484)
2: Scan ES-
1.01e6
672
626
670
628
673
629
674
630
675
97
113
163
222
249
325
407
420
522
553
599
762
809
844
939
985
999
1021
1155
1219
1232
m/z

SLE168 1H

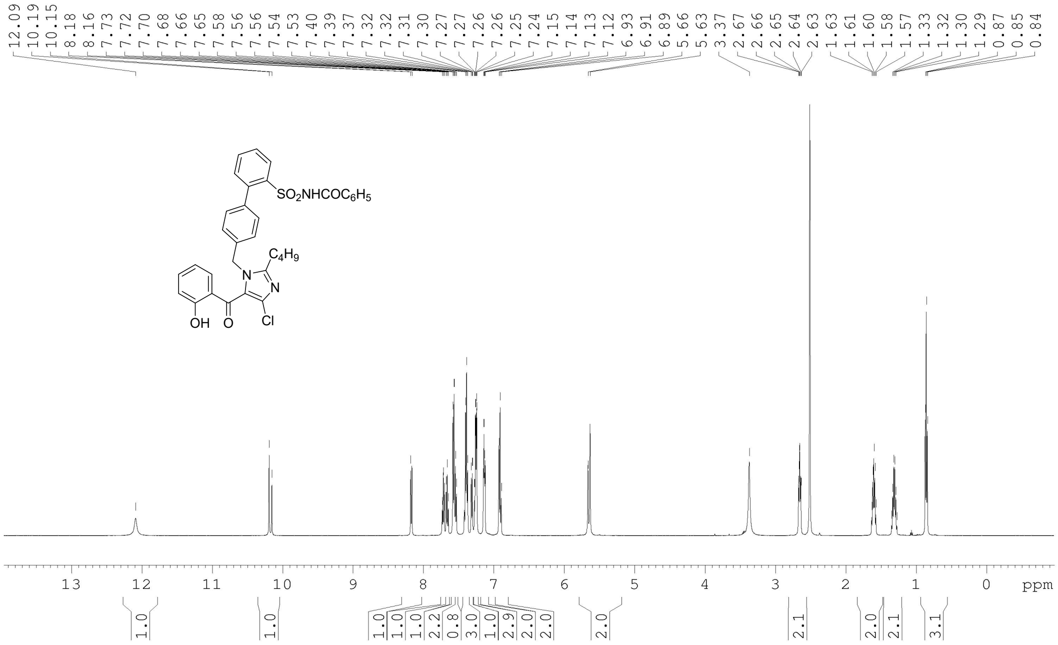

SLE168 DMSO 13C 126 MHz

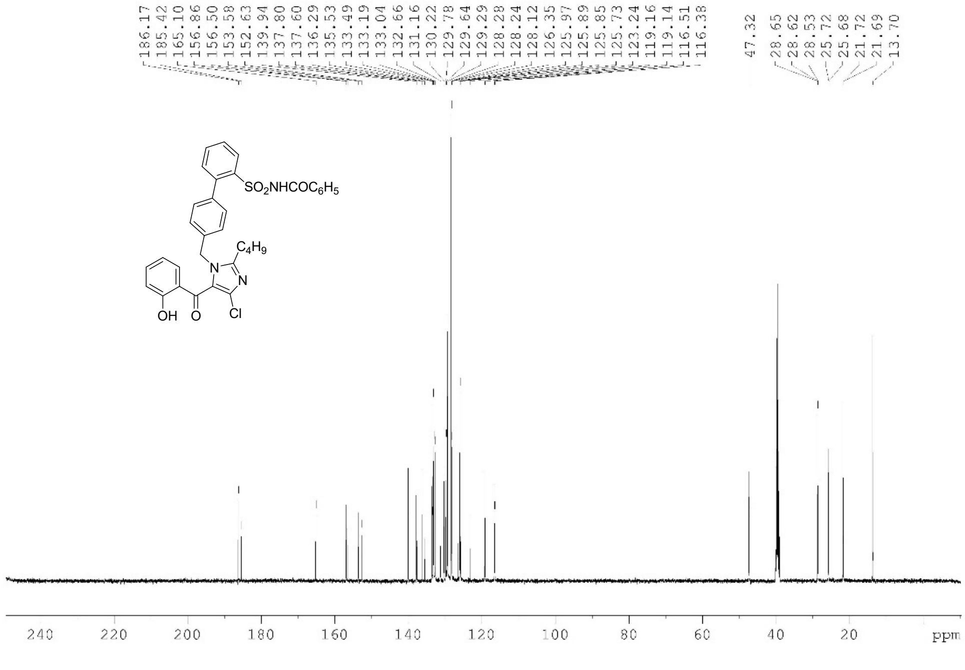

Single Mass Analysis
Tolerance = 5.0 mDa / DBE: min = -1.5, max = 50.0
Selected filters: None

Monoisotopic Mass, Even Electron Ions
154 formula(e) evaluated with 1 results within limits (up to 1000 best isotopic matches for each mass)
Elements Used:
C: 0-34 H: 0-31 N: 0-3 O: 0-5 F: 0-1 S: 0-1 Cl: 0-1
SYMA
SLE168-HRMS 114 (2.669)

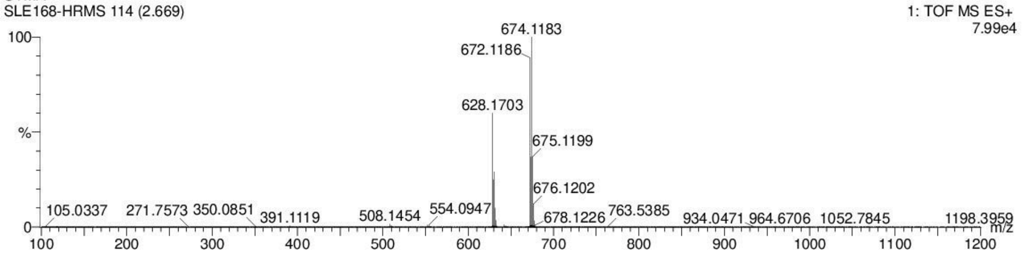

Minimum:				-1.5		
Maximum:		5.0	10.0	50.0		
Mass	Calc. Mass	mDa	PPM	DBE	i-FIT	Formula
628.1703	628.1673	3.0	4.8	20.5	35.7	C34 H31 N3 O5 S Cl

4'-((2-Butyl-4-chloro-5-(2-methoxybenzyl)-1H-imidazol-1-yl)methyl)-[1,1'-biphenyl]-2-sulfonamide (13).

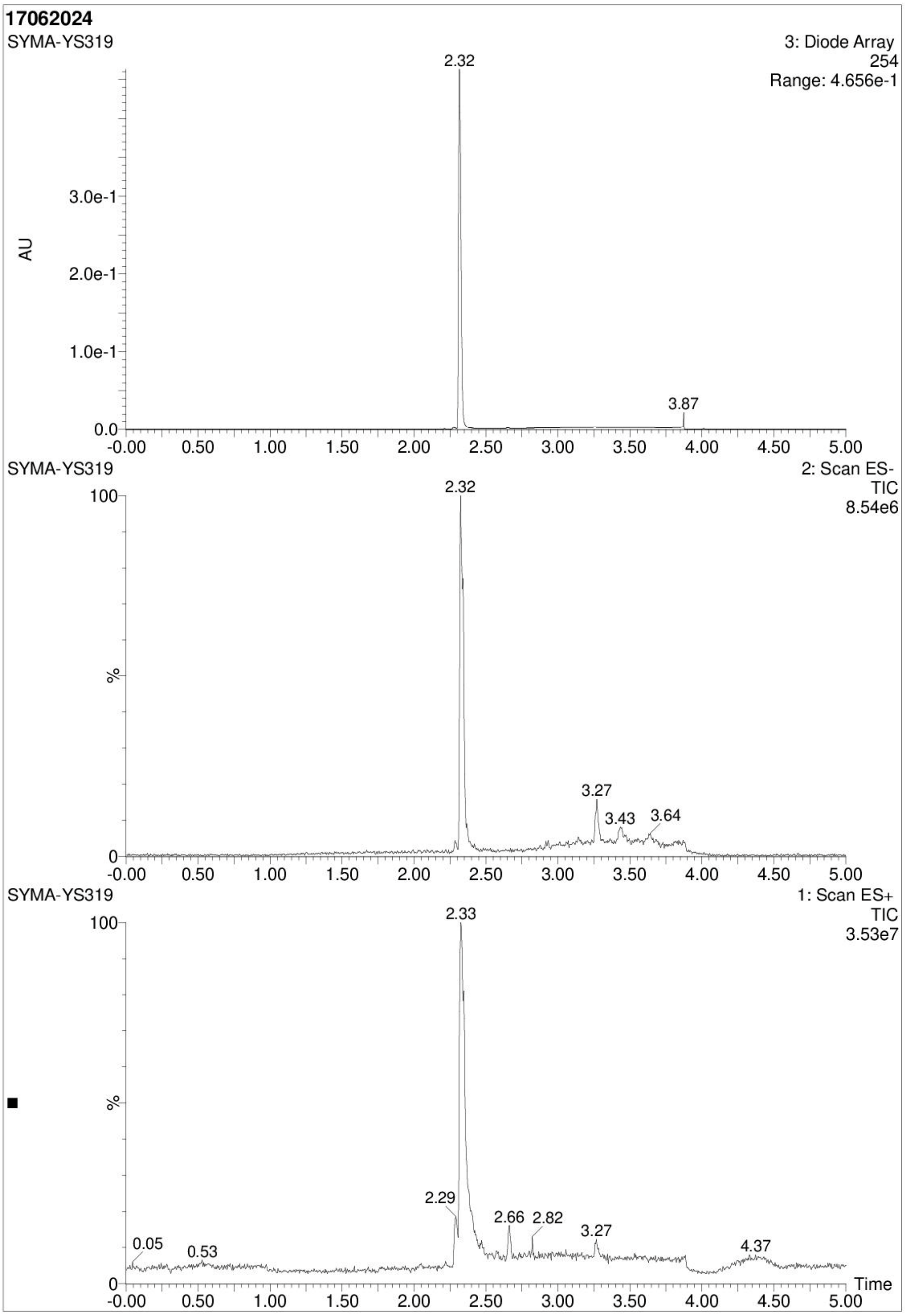

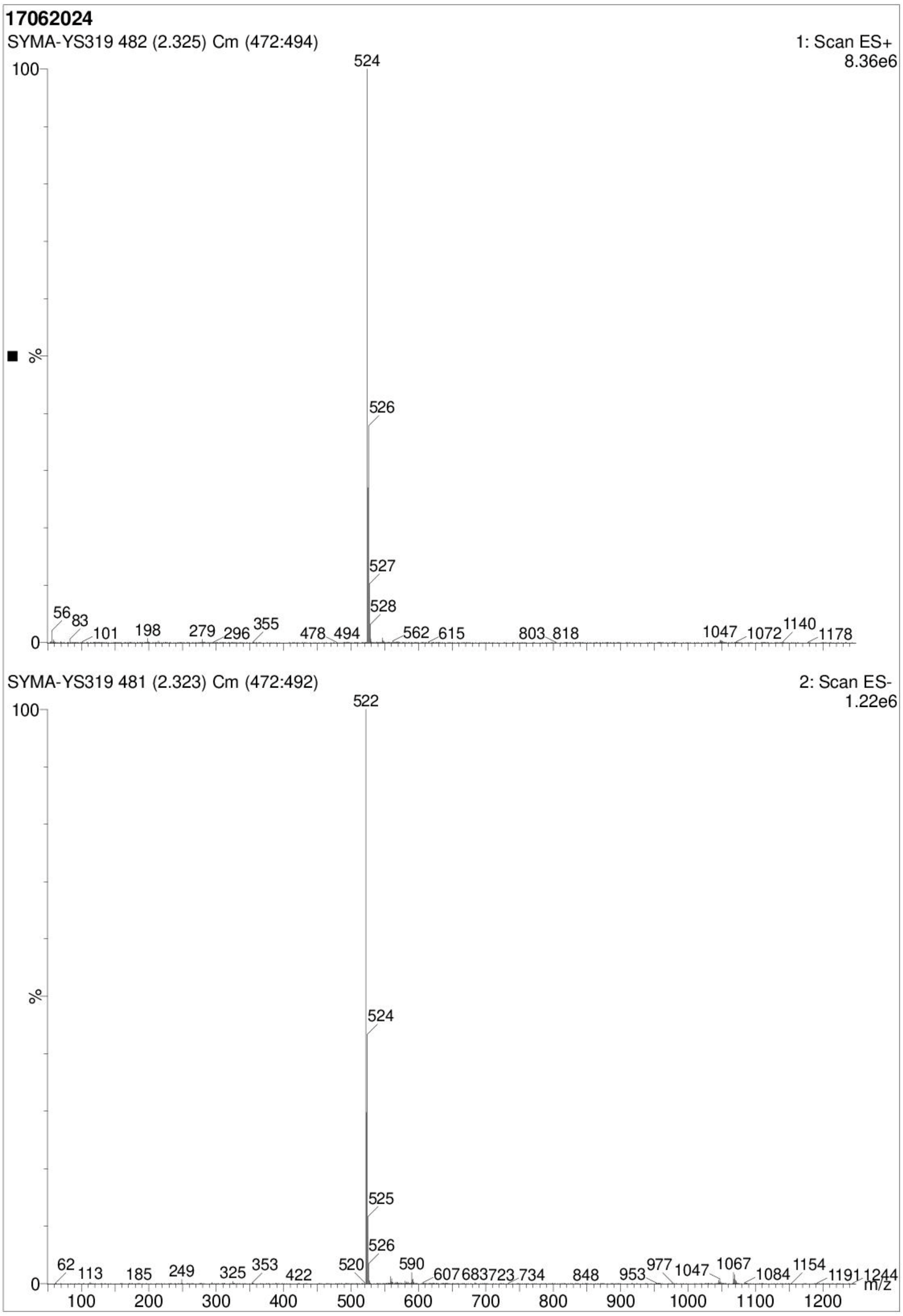
17062024
SYMA-YS319 482 (2.325) Cm (472:494)
1: Scan ES+
8.36e6
100
%
0
524
526
527
528
56
83
101
198
279
296
355
478
494
562
615
803
818
1047
1072
1140
1178
SYMA-YS319 481 (2.323) Cm (472:492)
2: Scan ES-
1.22e6
522
524
525
526
62
113
185
249
325
353
422
520
590
607
683
723
734
848
953
977
1047
1067
1084
1154
1191
1244
m/z
100
200
300
400
500
600
700
800
900
1000
1100
1200

YS319 DMSO 1H 500 MHz

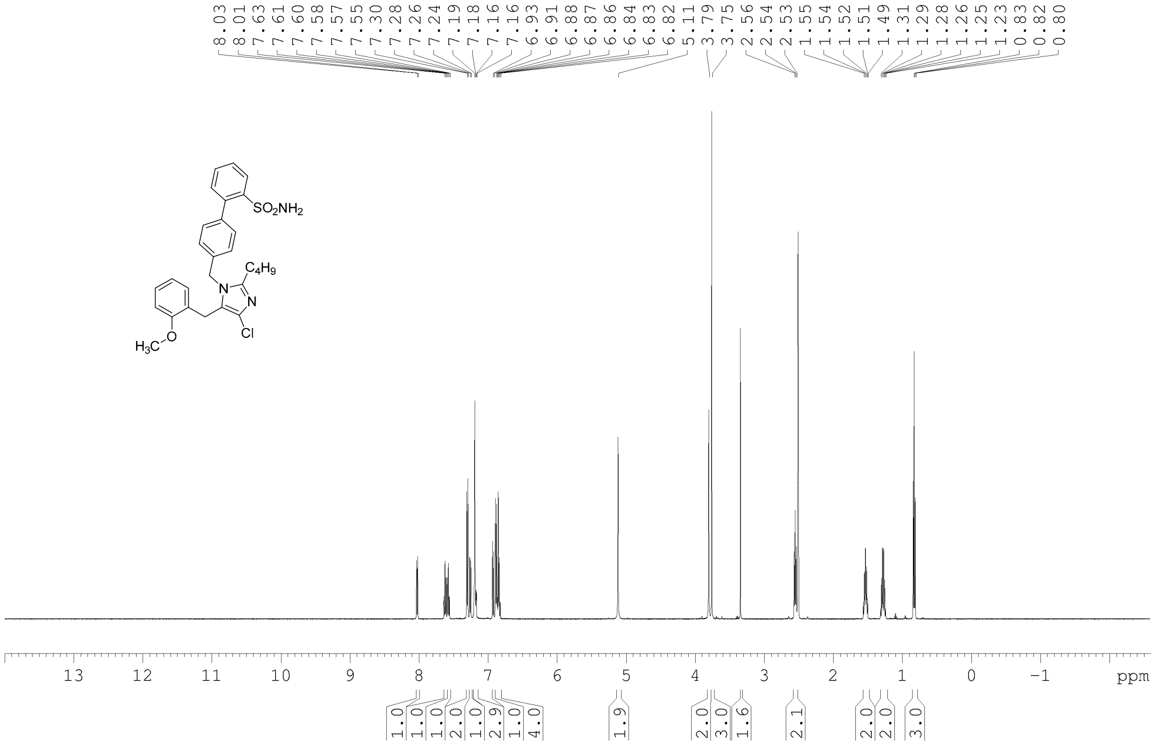

YS319 DMSO 13C 126 MHz

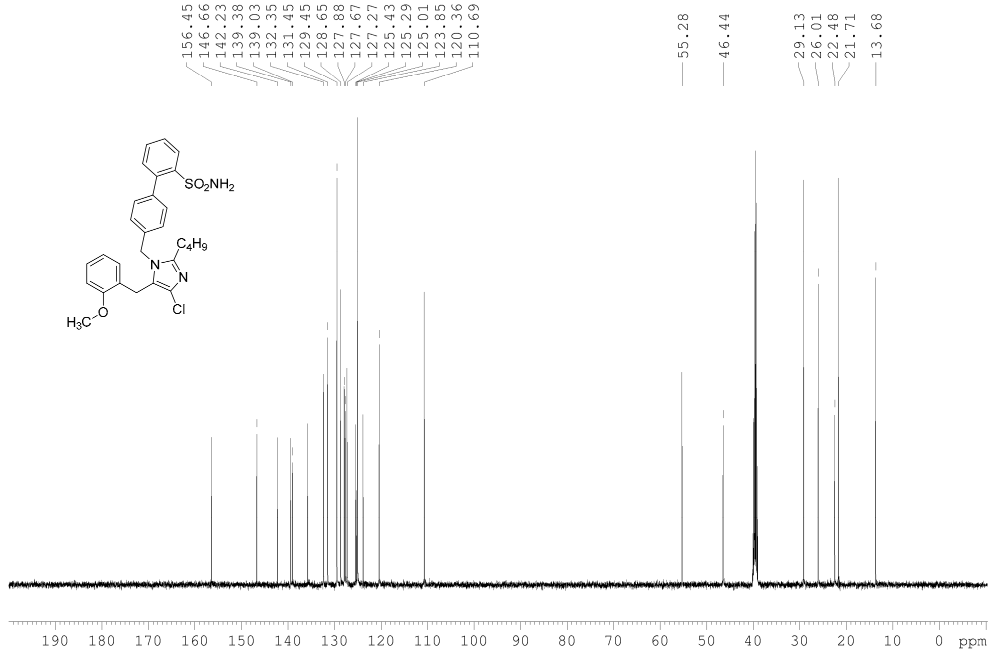

Single Mass Analysis
Tolerance = 5.0 mDa / DBE: min = -1.5, max = 50.0
Selected filters: None

Monoisotopic Mass, Even Electron Ions
97 formula(e) evaluated with 1 results within limits (up to 1000 best isotopic matches for each mass)
Elements Used:
C: 0-29 H: 0-31 N: 0-3 O: 0-3 F: 0-1 S: 0-1 Cl: 0-1

SYMA
YS319-HRMS 117 (2.730) 1: TOF MS ES+ 7.00e4

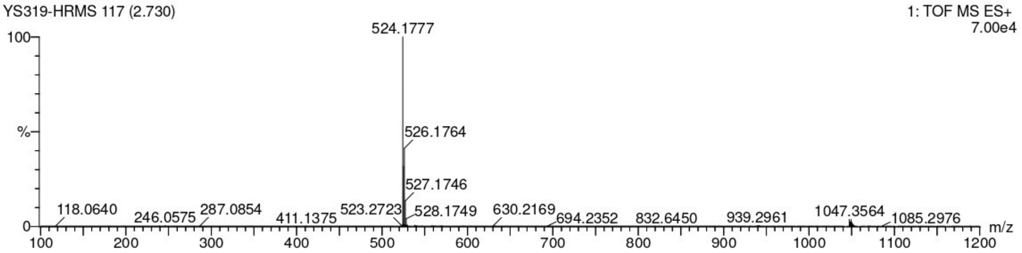

Minimum:				-1.5		
Maximum:		5.0	10.0	50.0		
Mass	Calc. Mass	mDa	PPM	DBE	i-FIT	Formula
524.1777	524.1775	0.2	0.4	14.5	34.2	C28 H31 N3 O3 S Cl

***N*-((4'-((2-Butyl-4-chloro-5-(2-methoxybenzyl)-1H-imidazol-1-yl)methyl)-[1,1'-biphenyl]-2-yl)sulfonyl) benzamide (14a).**

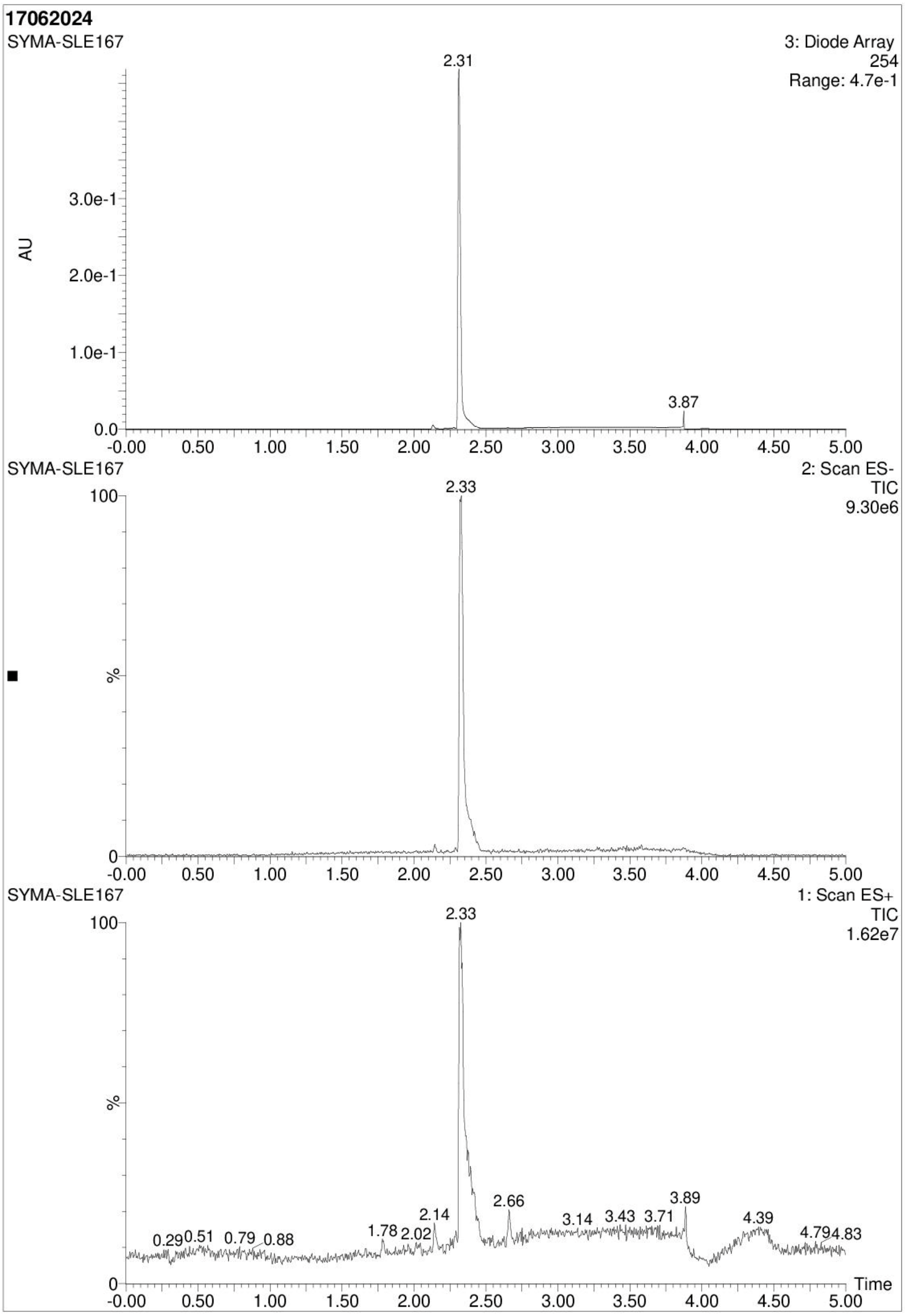

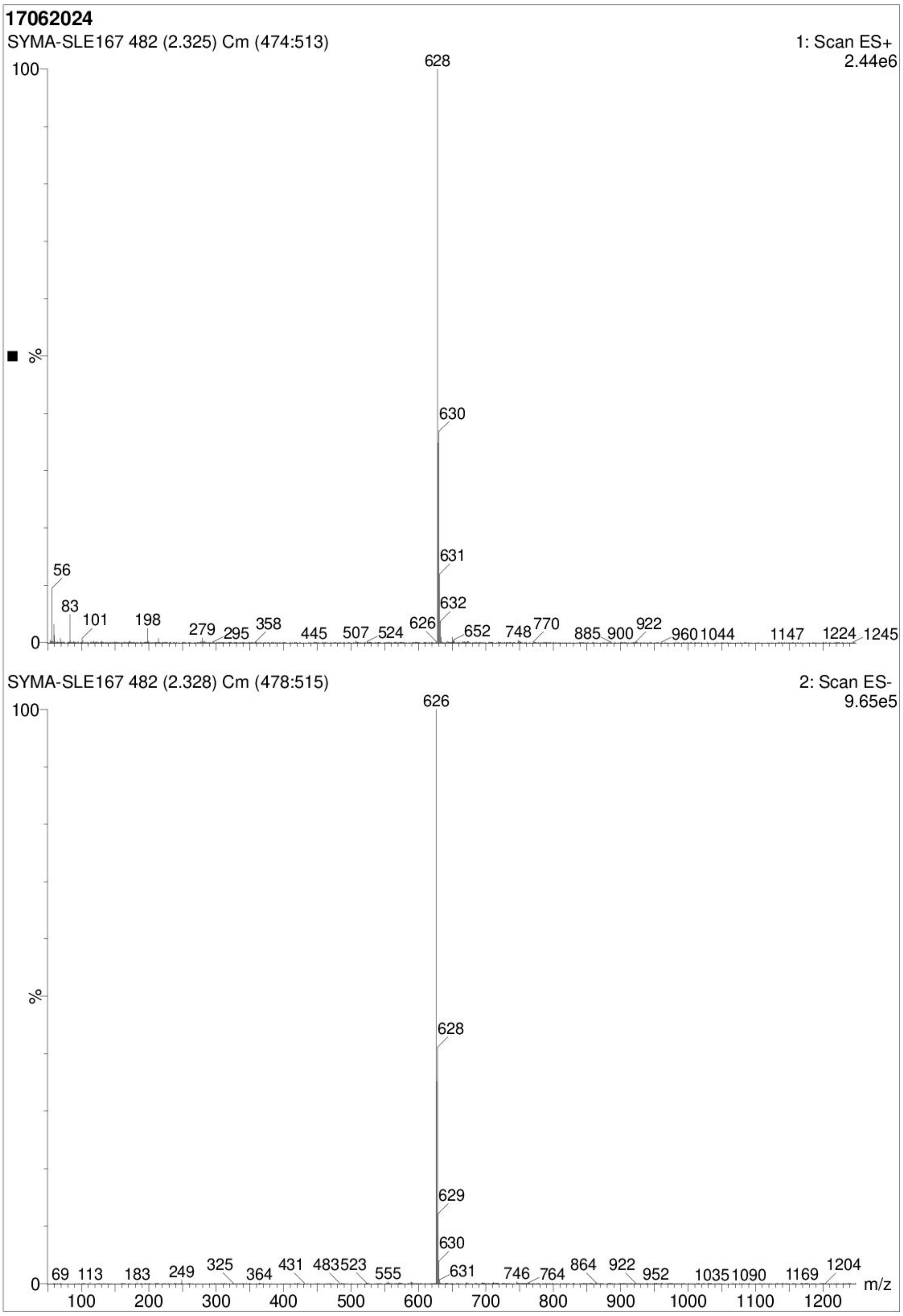
17062024
SYMA-SLE167 482 (2.325) Cm (474:513)
1: Scan ES+
2.44e6
628
630
631
632
56
83
101
198
279
295
358
445
507
524
626
652
748
770
885
900
922
960
1044
1147
1224
1245
SYMA-SLE167 482 (2.328) Cm (478:515)
2: Scan ES-
9.65e5
626
628
629
630
69
113
183
249
325
364
431
483
523
555
631
746
764
864
922
952
1035
1090
1169
1204
%
m/z
0
100
200
300
400
500
600
700
800
900
1000
1100
1200

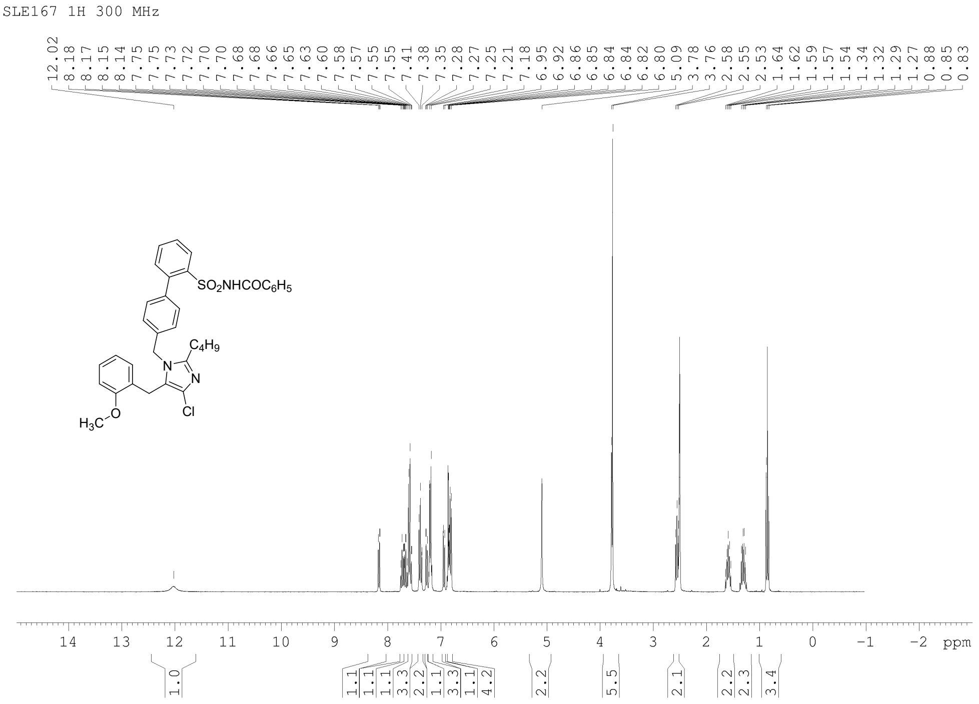
SLE167 1H 300 MHz
12.02 8.18 8.17 8.15 8.14 7.75 7.75 7.73 7.72 7.70 7.70 7.68 7.68 7.66 7.65 7.63 7.60 7.58 7.57 7.55 7.55 7.41 7.38 7.35 7.28 7.27 7.25 7.21 7.18 6.95 6.92 6.86 6.85 6.84 6.84 6.82 6.82 6.80 5.09 3.78 3.76 2.58 2.58 2.55 2.53 1.64 1.62 1.59 1.57 1.54 1.34 1.32 1.29 1.27 0.88 0.85 0.83
SO2NHCOC6H5
C4H9
N
N
Cl
H3C
O
14 13 12 11 10 9 8 7 6 5 4 3 2 1 0 −1 −2 ppm
1.0 1.1 1.1 1.1 3.3 2.2 1.1 3.3 1.1 4.2 2.2 5.5 2.1 2.2 2.3 3.4

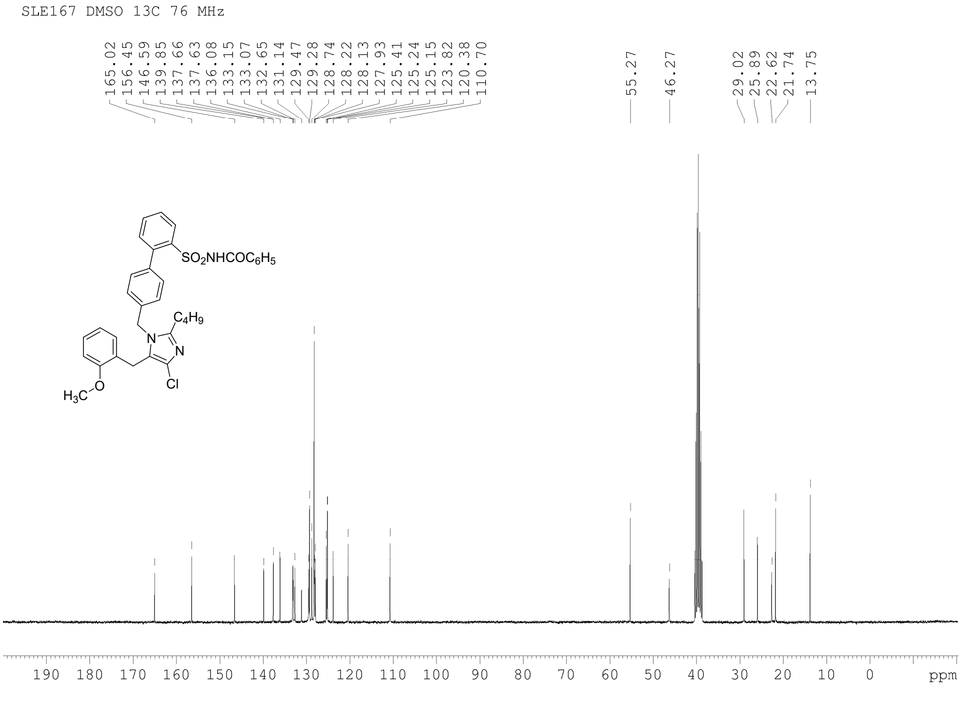
SLE167 DMSO 13C 76 MHz
165.02 156.45 146.59 139.85 137.66 137.63 136.08 133.15 133.07 132.65 131.14 129.47 129.28 128.74 128.22 128.13 127.93 125.41 125.24 125.15 123.82 120.38 110.70
55.27
46.27
29.02 25.89 22.62 21.74 13.75
SO2NHCOC6H5
C4H9
N
N
Cl
H3C
O
190 180 170 160 150 140 130 120 110 100 90 80 70 60 50 40 30 20 10 0 ppm

Elemental Composition Report

Single Mass Analysis

Tolerance = 5.0 mDa / DBE: min = -1.5, max = 50.0
Selected filters: None

Monoisotopic Mass, Even Electron Ions
123 formula(e) evaluated with 1 results within limits (up to 1000 best isotopic matches for each mass)
Elements Used:
C: 0-35 H: 0-35 N: 0-3 O: 0-4 F: 0-1 S: 0-1 Cl: 0-1

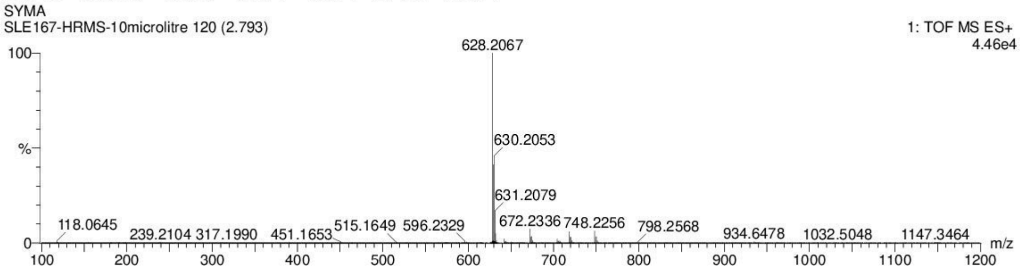

Minimum:				-1.5		
Maximum:		5.0	10.0	50.0		
Mass	Calc. Mass	mDa	PPM	DBE	i-FIT	Formula
628.2067	628.2037	3.0	4.8	19.5	7.2	C35 H35 N3 O4 S Cl

4'-((2-Butyl-4-chloro-5-(2-methoxybenzyl)-1H-imidazol-1-yl)methyl)-N-(cyclohexylcarbamoyl)-[1,1'-biphenyl]-2-sulfonamide (14b).

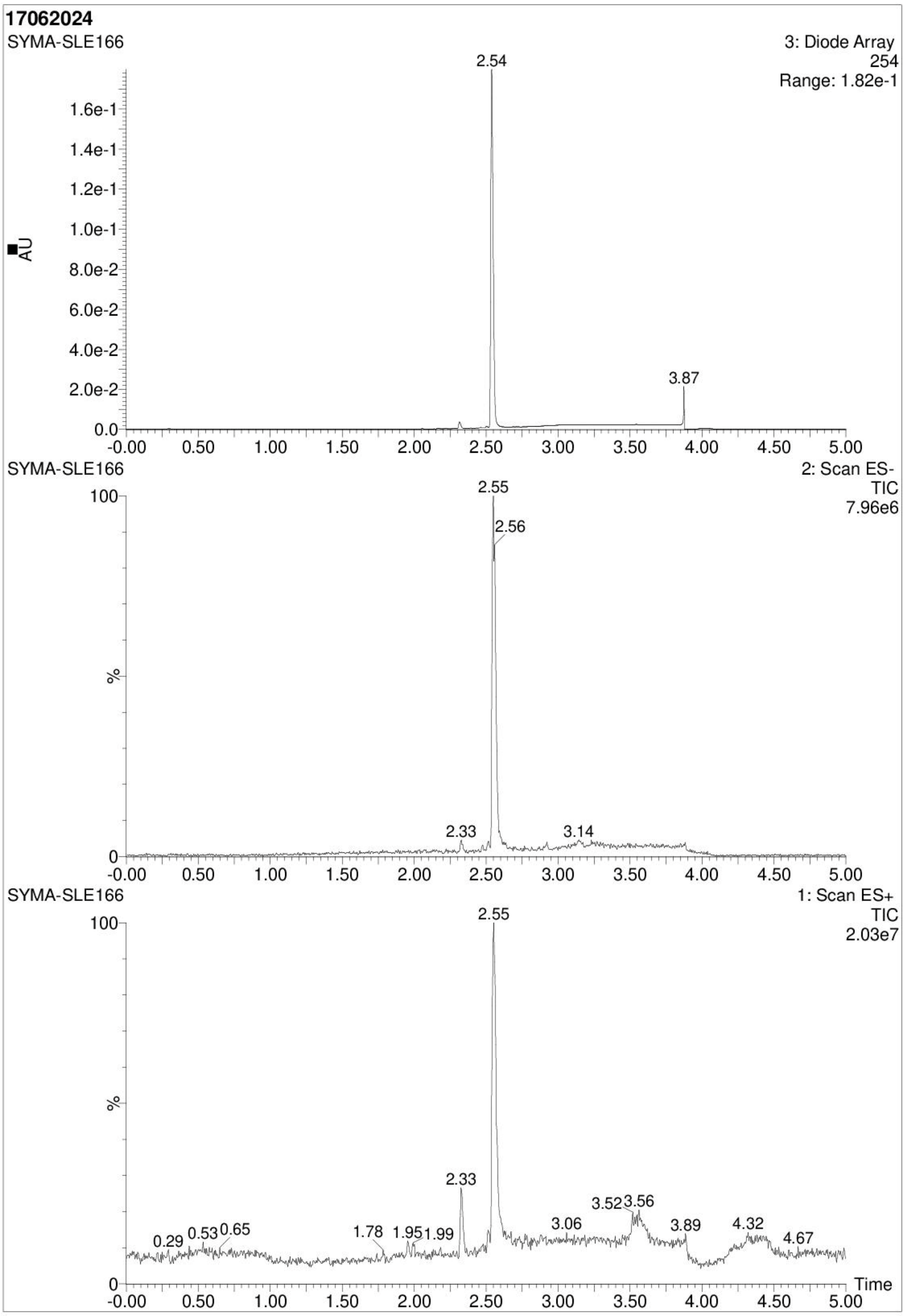

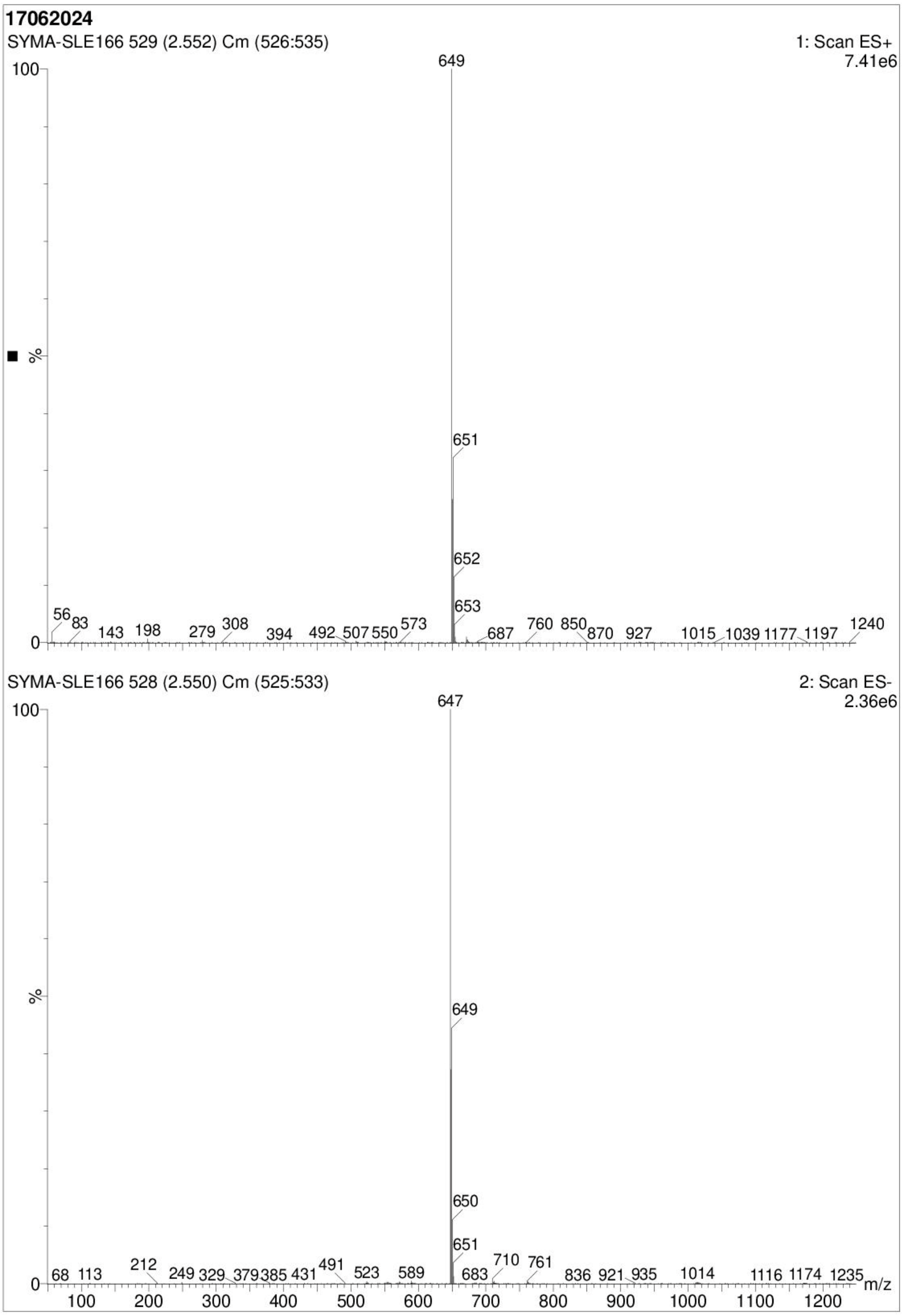
17062024
SYMA-SLE166 529 (2.552) Cm (526:535)
1: Scan ES+
7.41e6
100
%
0
649
651
652
653
56 83 143 198 279 308 394 492 507 550 573 687 760 850 870 927 1015 1039 1177 1197 1240
SYMA-SLE166 528 (2.550) Cm (525:533)
2: Scan ES-
2.36e6
647
649
650
651
68 113 212 249 329 379 385 431 491 523 589 683 710 761 836 921 935 1014 1116 1174 1235
100 200 300 400 500 600 700 800 900 1000 1100 1200
m/z

SLE166 1H 500MHz

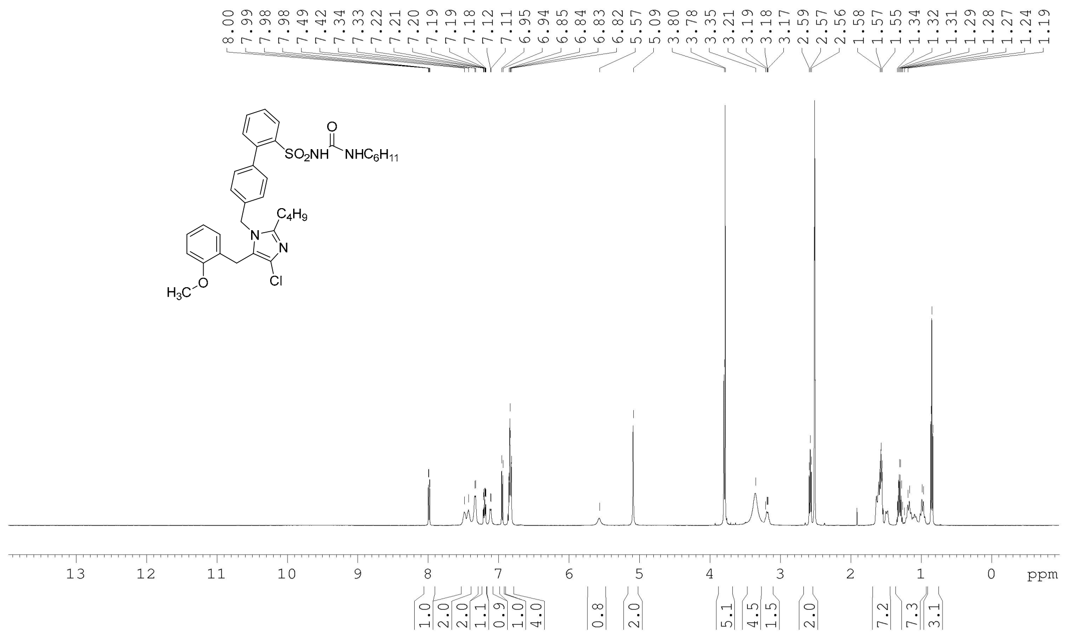

SLE166 13C 126MHz

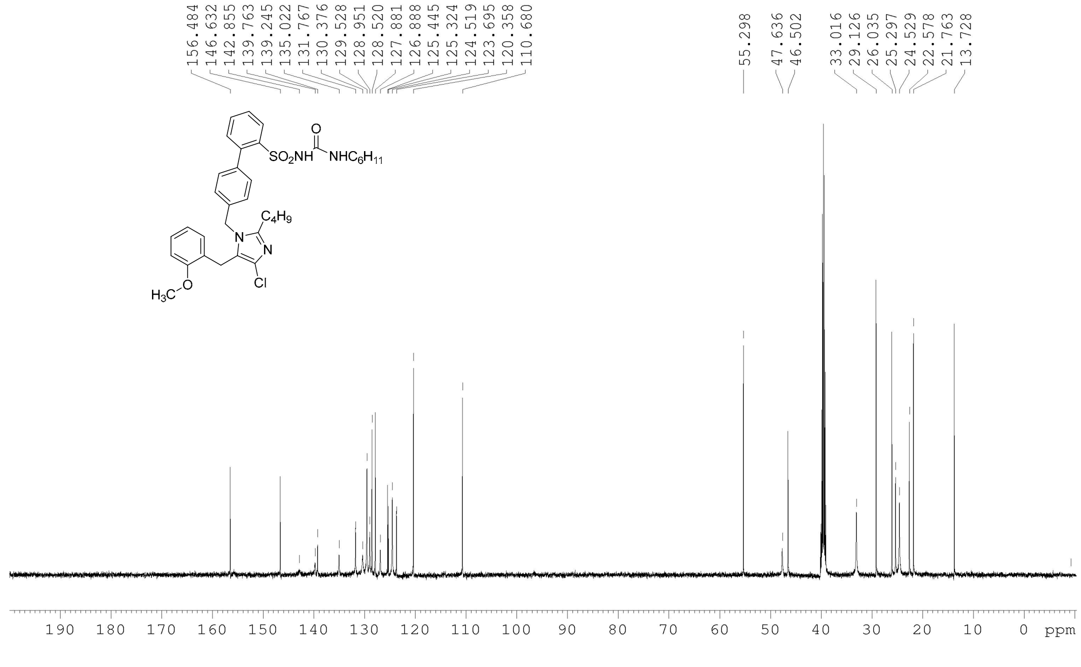

Elemental Composition Report

Single Mass Analysis
Tolerance = 5.0 mDa / DBE: min = -1.5, max = 50.0
Selected filters: None

Monoisotopic Mass, Even Electron Ions
179 formula(e) evaluated with 1 results within limits (up to 1000 best isotopic matches for each mass)
Elements Used:
C: 0-35 H: 0-42 N: 0-4 O: 0-4 F: 0-1 S: 0-1 Cl: 0-1

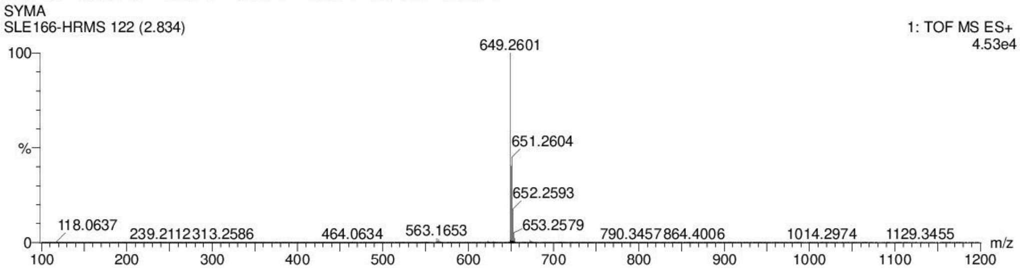

Minimum:				-1.5		
Maximum:		5.0	10.0	50.0		
Mass	Calc. Mass	mDa	PPM	DBE	i-FIT	Formula
649.2601	649.2615	-1.4	-2.2	16.5	12.3	C35 H42 N4 O4 S Cl

Methyl 2-(4-((2-butyl-4-chloro-5-(2-hydroxybenzyl)-1H-imidazol-1-yl)methyl)benzoyl)benzoate (15).

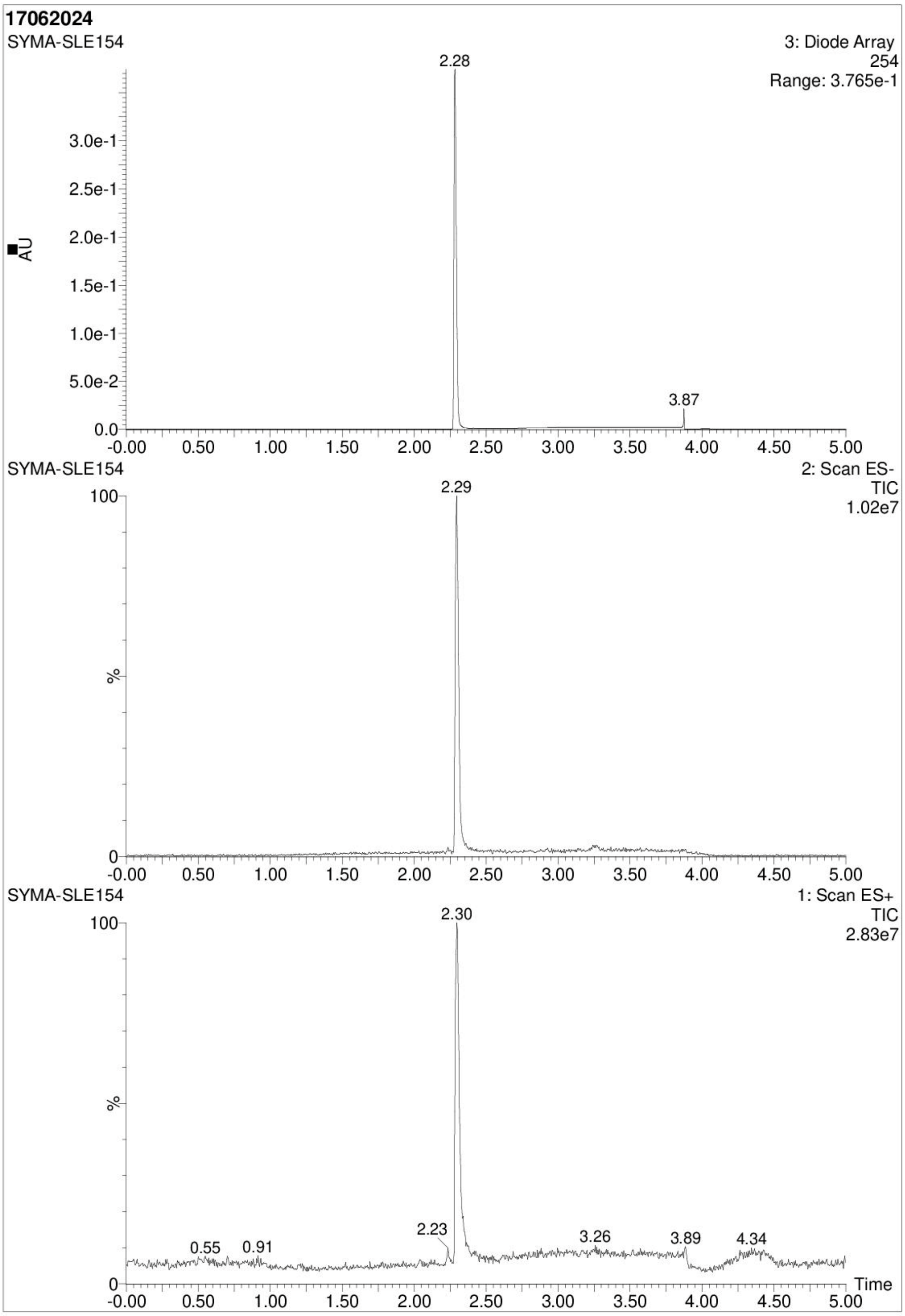

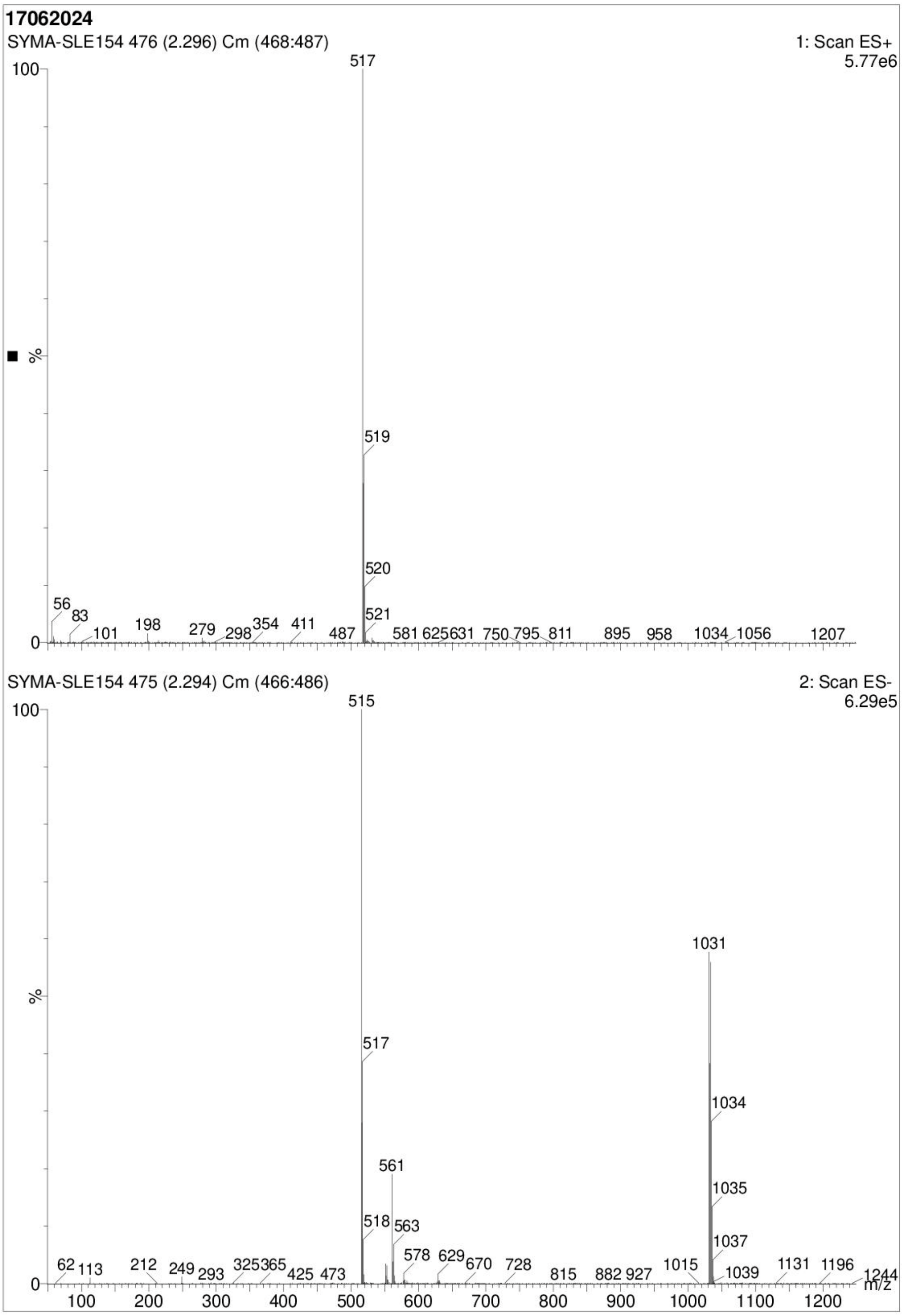
17062024
SYMA-SLE154 476 (2.296) Cm (468:487)
1: Scan ES+
5.77e6
517
519
520
521
56
83
101
198
279
298
354
411
487
581
625
631
750
795
811
895
958
1034
1056
1207
SYMA-SLE154 475 (2.294) Cm (466:486)
2: Scan ES-
6.29e5
515
1031
517
1034
561
1035
518
563
1037
578
629
670
728
62
113
212
249
293
325
365
425
473
815
882
927
1015
1039
1131
1196
1244
%
m/z
0
100
100
200
300
400
500
600
700
800
900
1000
1100
1200

SLE154 1H

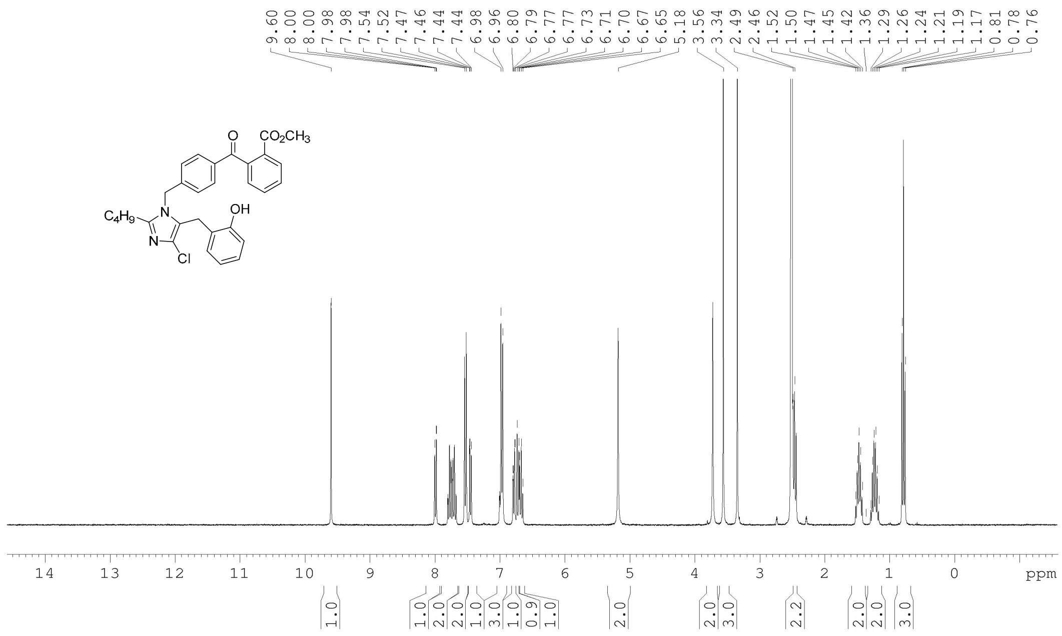

SLE154 DMSO 13C

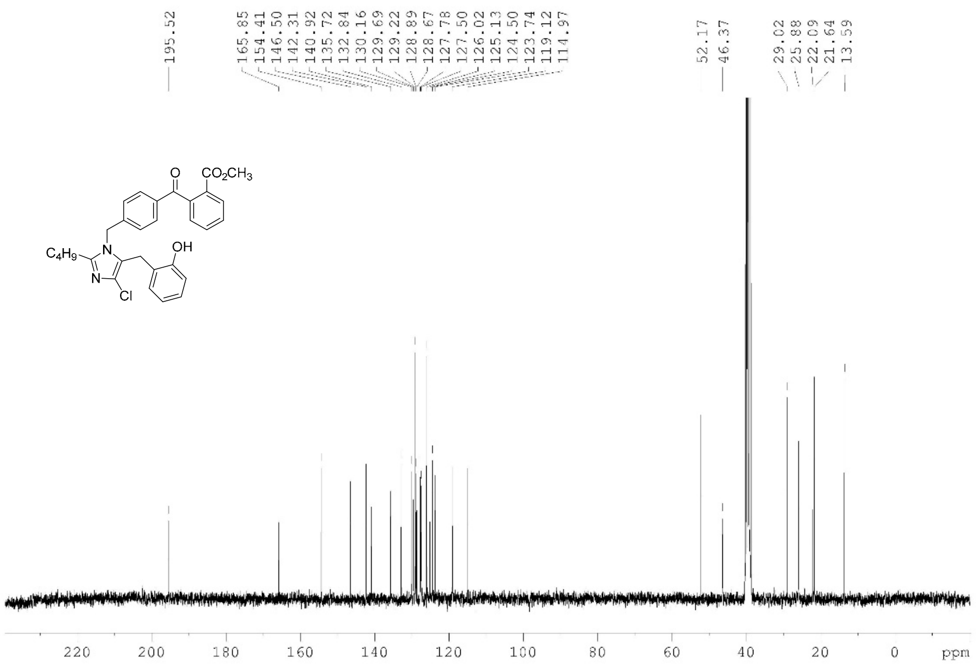

Elemental Composition Report

Single Mass Analysis

Tolerance = 5.0 mDa / DBE: min = -1.5, max = 50.0
Selected filters: None

Monoisotopic Mass, Even Electron Ions
46 formula(e) evaluated with 1 results within limits (up to 1000 best isotopic matches for each mass)
Elements Used:
C: 0-30 H: 0-30 N: 0-2 O: 0-4 F: 0-1 Cl: 0-1

SYMA
SLE154-HRMS 114 (2.668)

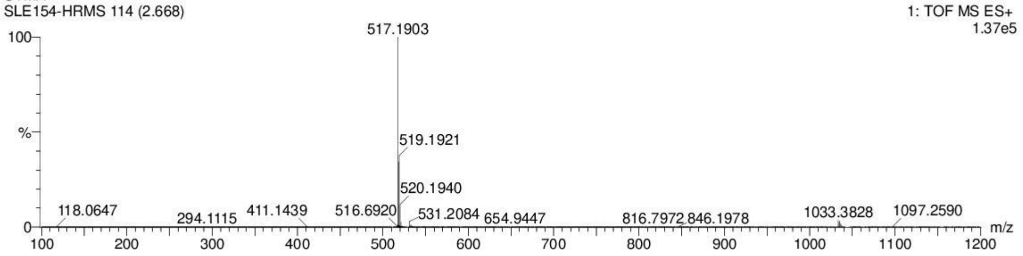

	Calc. Mass	mDa	PPM	DBE	i-FIT	Formula
Minimum:				-1.5		
Maximum:		5.0	10.0	50.0		

Mass	Calc. Mass	mDa	PPM	DBE	i-FIT	Formula
517.1903	517.1894	0.9	1.7	16.5	34.4	C30 H30 N2 O4 Cl

2-(4-((2-Butyl-4-chloro-5-(2-hydroxybenzyl)-1H-imidazol-1-yl)methyl)benzoyl)benzoic acid (16).

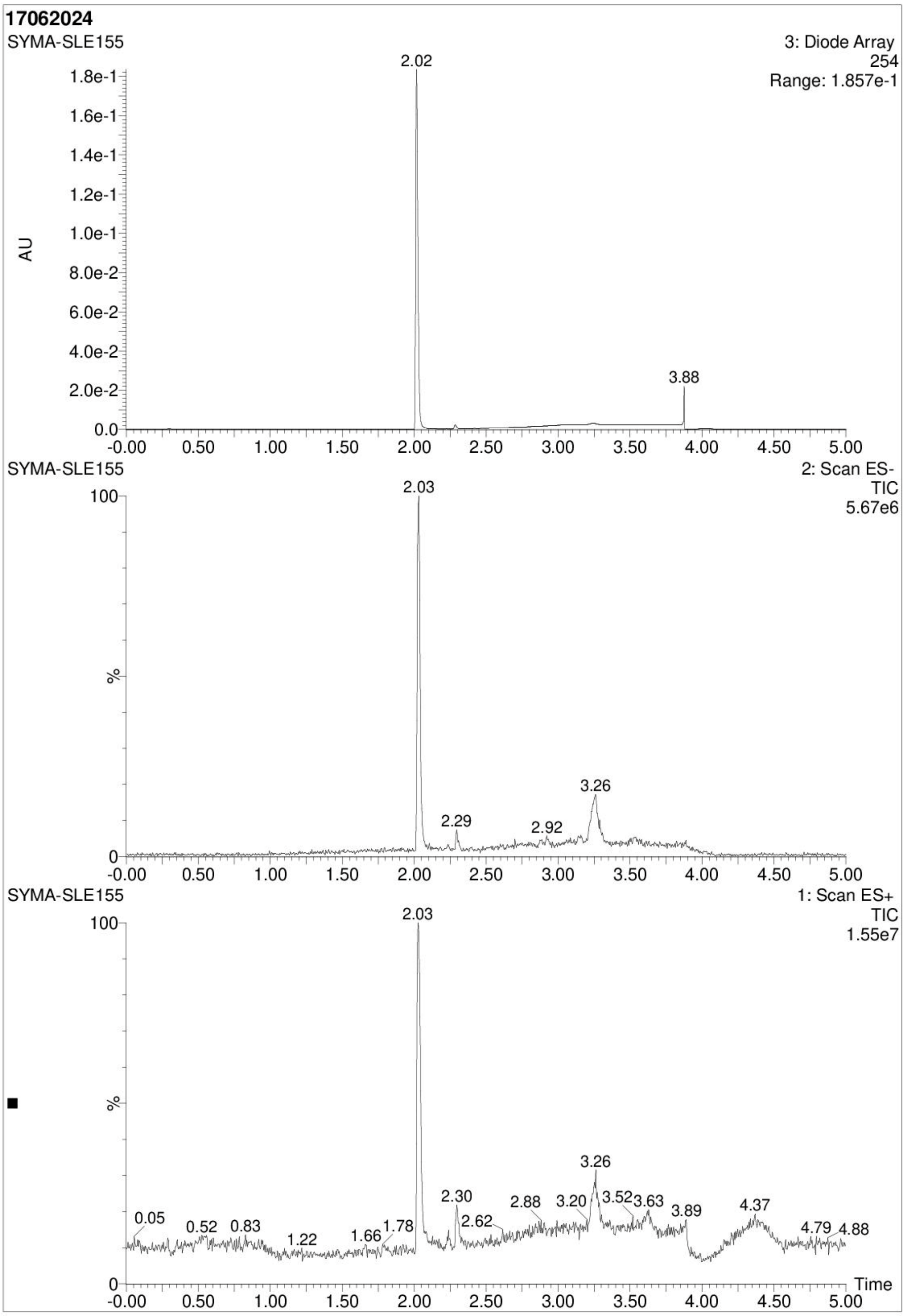

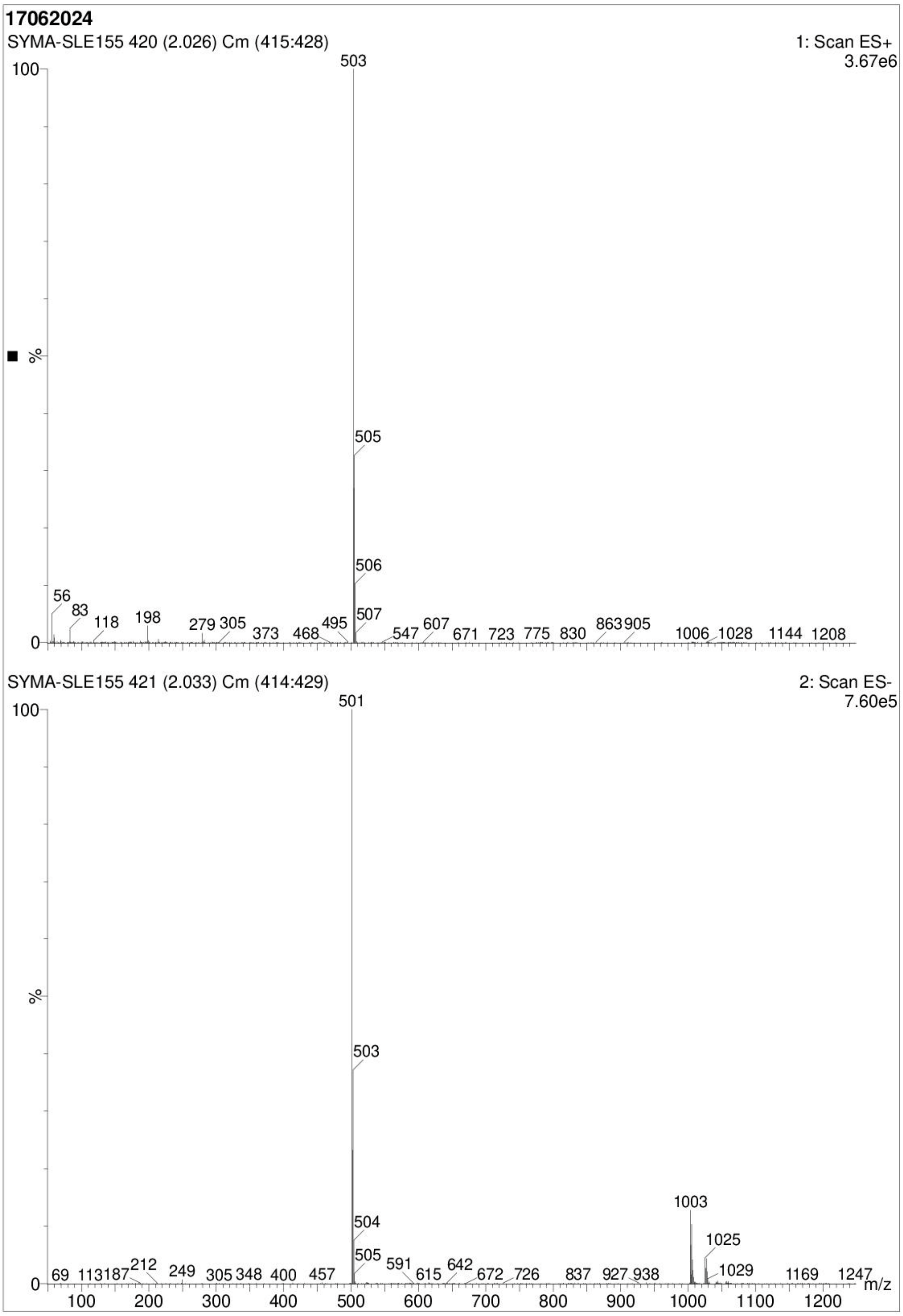
17062024
SYMA-SLE155 420 (2.026) Cm (415:428)
1: Scan ES+
3.67e6
SYMA-SLE155 421 (2.033) Cm (414:429)
2: Scan ES-
7.60e5
%
m/z

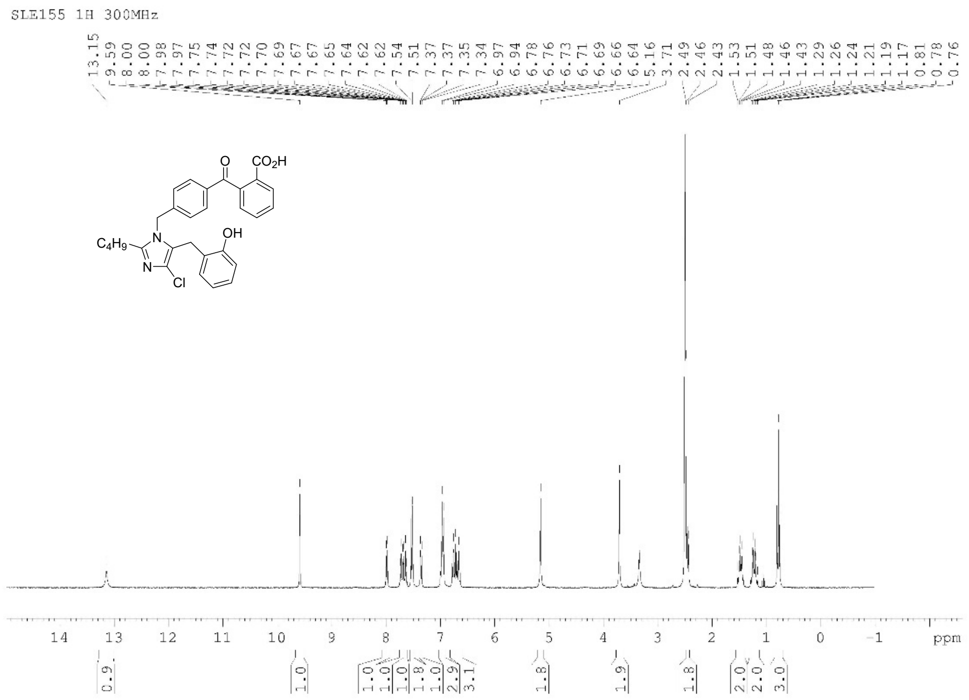
SLE155 1H 300MHz
13.15
9.59
8.00
8.00
7.98
7.97
7.75
7.74
7.72
7.72
7.70
7.69
7.67
7.67
7.65
7.64
7.62
7.62
7.54
7.51
7.37
7.37
7.35
7.34
6.97
6.94
6.78
6.76
6.73
6.71
6.69
6.66
6.64
5.16
3.71
2.49
2.46
2.43
1.53
1.51
1.48
1.46
1.43
1.29
1.26
1.24
1.21
1.19
1.17
0.81
0.78
0.76
CO2H
O
OH
C4H9
N
N
Cl
14
13
12
11
10
9
8
7
6
5
4
3
2
1
0
-1
ppm
0.9
1.0
1.0
1.0
1.0
1.8
1.0
2.9
3.1
1.8
1.9
1.8
2.0
2.0
3.0

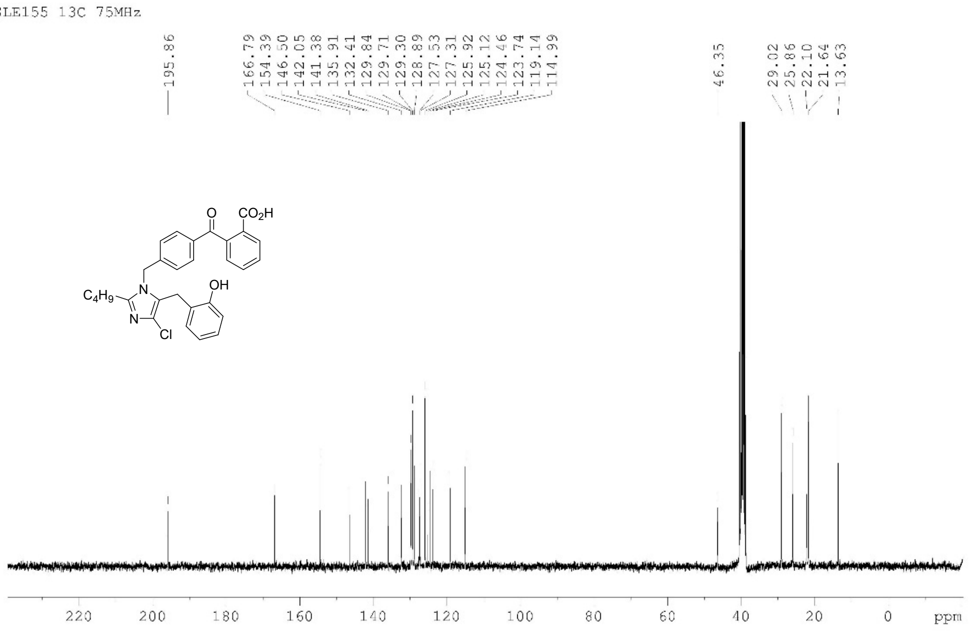
SLE155 13C 75MHz
195.86
166.79
154.39
146.50
142.05
141.38
135.91
132.41
129.84
129.71
129.30
128.89
127.53
127.31
125.92
125.12
124.46
123.74
119.14
114.99
46.35
29.02
25.86
22.10
21.64
13.63
CO2H
O
OH
C4H9
N
N
Cl
220
200
180
160
140
120
100
80
60
40
20
0
ppm

Elemental Composition Report **Page 1**

Single Mass Analysis
Tolerance = 5.0 mDa / DBE: min = -1.5, max = 50.0
Selected filters: None

Monoisotopic Mass, Even Electron Ions
46 formula(e) evaluated with 1 results within limits (up to 1000 best isotopic matches for each mass)
Elements Used:
C: 0-29 H: 0-28 N: 0-2 O: 0-4 F: 0-1 Cl: 0-1

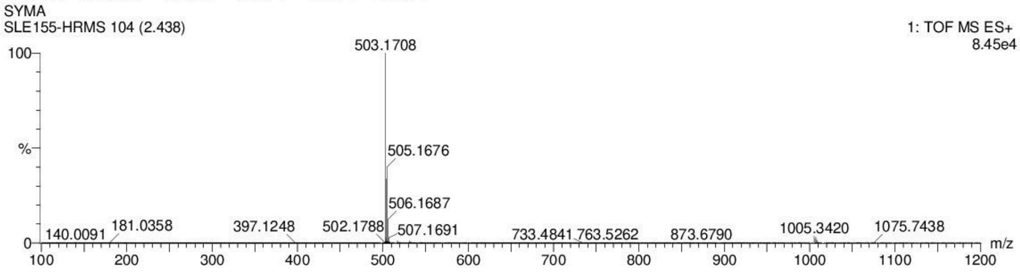

Minimum:				-1.5		
Maximum:		5.0	10.0	50.0		
Mass	Calc. Mass	mDa	PPM	DBE	i-FIT	Formula
503.1708	503.1738	-3.0	-6.0	16.5	40.9	C29 H28 N2 O4 Cl
